# Pentose sugars inhibit metabolism and increase expression of an AgrD-type cyclic pentapeptide in *Clostridium thermocellum*

**DOI:** 10.1038/srep43355

**Published:** 2017-02-23

**Authors:** Tobin J. Verbeke, Richard J. Giannone, Dawn M. Klingeman, Nancy L. Engle, Thomas Rydzak, Adam M. Guss, Timothy J. Tschaplinski, Steven D. Brown, Robert L. Hettich, James G. Elkins

**Affiliations:** 1 BioEnergy Science Center, Oak Ridge National Laboratory, Oak Ridge, TN, 37831, USA; 2 Biosciences Division, Oak Ridge National Laboratory, Oak Ridge, TN, 37831, USA; 3 Chemical Sciences Division, Oak Ridge National Laboratory, Oak Ridge, TN, 37831, USA.

## Abstract

*Clostridium thermocellum* could potentially be used as a microbial biocatalyst to produce renewable fuels directly from lignocellulosic biomass due to its ability to rapidly solubilize plant cell walls. While the organism readily ferments sugars derived from cellulose, pentose sugars from xylan are not metabolized. Here, we show that non-fermentable pentoses inhibit growth and end-product formation during fermentation of cellulose-derived sugars. Metabolomic experiments confirmed that xylose is transported intracellularly and reduced to the dead-end metabolite xylitol. Comparative RNA-seq analysis of xylose-inhibited cultures revealed several up-regulated genes potentially involved in pentose transport and metabolism, which were targeted for disruption. Deletion of the ATP-dependent transporter, CbpD partially alleviated xylose inhibition. A putative xylitol dehydrogenase, encoded by Clo1313_0076, was also deleted resulting in decreased total xylitol production and yield by 41% and 46%, respectively. Finally, xylose-induced inhibition corresponds with the up-regulation and biogenesis of a cyclical AgrD-type, pentapeptide. Medium supplementation with the mature cyclical pentapeptide also inhibits bacterial growth. Together, these findings provide new foundational insights needed for engineering improved pentose utilizing strains of *C. thermocellum* and reveal the first functional Agr-type cyclic peptide to be produced by a thermophilic member of the Firmicutes.

The cellulolytic capabilities of the thermophilic anaerobe, *Clostridium thermocellum*, have marked this bacterium as a potential biocatalyst for lignocellulosic biofuel production through consolidated bioprocessing (CBP)^1^,^2^. *C. thermocellum* colonizes plant cell walls where it utilizes a diverse suite of lignocellulose deconstructing enzymes to generate fermentable sugars^3^,^4^. Despite its extensive repertoire of hydrolytic enzymes, the microbe is limited to the products of cellulose hydrolysis (cellodextrins) to support metabolism and growth. As a result, other plant cell wall polymers, principally hemicellulose and lignin, and their depolymerization products increase in relative abundance during the course of cellodextrin fermentation.

Potential bioenergy crops, such as *Miscanthus*, switchgrass and *Populus*, contain xylan-based hemicelluloses^5^,^6^,^7^. Further, recent CBP developments have focused on minimally pretreated feedstocks, which retain hemicellulose polymers in the substrate^8^,^9^. Despite being limited to cellodextrins for fermentation, multiple reports show that *C. thermocellum* is highly capable of deconstructing and solubilizing hemicellulose polymers as a means of improving accessibility to cellulose^10^,^11^,^12^. It has also been observed that during fermentation, total xylan and total glucan solubilization strongly correlate with xylan solubilization efficiencies of 60–70% being reported^9^,^13^. At industrially relevant biomass loadings (i.e. >100 g/L total solids) it is therefore conceivable that hemicellulose hydrolysis products may accumulate in gram per litre or tens of grams per litre concentrations. Further, high solids loading fermentations are known to experience mass transfer issues leading to high localized concentrations of sugars that can both inhibit continued hydrolysis as well as fermentation rate^14^,^15^.

In nature, the products of *C. thermocellum* mediated hemicellulose deconstruction would typically be consumed by other saccharolytic, pentose-fermenting thermophiles such as *Thermoanaerobacter* and *Thermoanaerobacterium* spp^16^. In axenic culture, however, the removal of these saccharides while fermenting biomass would not occur. While *C. thermocellum* is able to hydrolyze hemicellulose polymers, but unable to catabolize pentoses, little effort has been given to understanding how the microorganism interacts with these residual saccharides. Despite the lack of catabolic activity, it is possible that: i) interactions occur; and ii) the nature of the interactions may be detrimental to the microbe. For example, despite its inability to ferment xylose, glucose uptake in *Saccharomyces cerevisiae* can be competitively inhibited by the pentose sugar^17^,^18^. Given the abundance of pentose sugars in lignocellulosic biomass, and the lack of xylose consumption during *C. thermocellum* pure culture fermentation on minimally pretreated biomass, the purpose of this study was to assess the impact of xylose and xylo-oligomers on the metabolism of the bacterium.

## Results

### Growth and inhibition analyses

**Figure 1:**
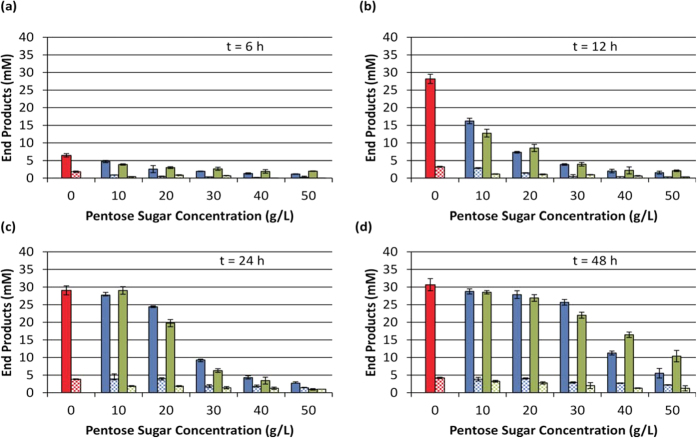
End-product formation in the presence of xylose and xylan by *C. thermocellum* M1570. Values represent average net production ± SD (n = 6) at 6 hours (**a**), 12 hours (**b**), 24 hours (**c**), or 48 hours (**d**) post-inoculation. Solid bars indicate ethanol concentration, while checkered bars indicate formate. Red = uninhibited control; blue = xylose addition; green = xylan addition. For clarity, lactate and acetate concentrations were omitted from the graph as concentrations were <1 mM after 48 hours for all conditions.

The model substrates xylose and Beechwood xylan were added at increasing concentrations to *C. thermocellum* M1570^19^ fermentation medium and their effect on end-product formation, as an indicator of metabolic limitation, was assessed (Fig. 1). Time course analyses revealed that both compounds significantly inhibited the rate of end-product accumulation (Fig. 1). While the level of inhibition was directly related to the concentration of xylosaccharide, under no condition was product formation completely inhibited. Rather, with extended time, end-product titres continued to increase and, at the lower concentrations tested, approached the levels observed in the uninhibited control. After 48 hours (Fig. 1D), reductions in titre across conditions correlated with reduced substrate consumption as residual cellobiose concentrations ranged from <1 mM (0 g/L, 10 g/L xylose, 10 g/L xylan) to ~9.5 mM (50 g/L xylose).

**Figure 2:**
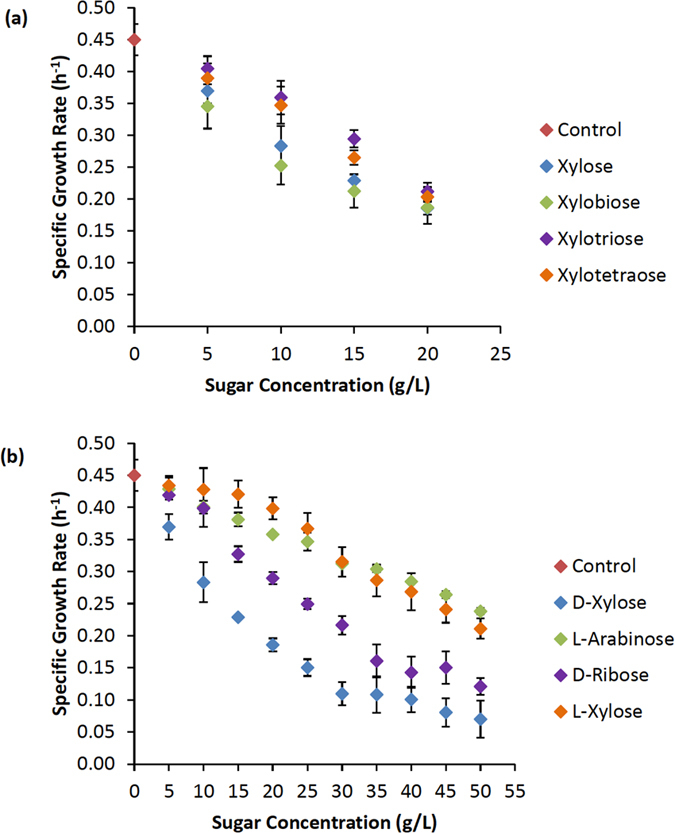
Effect of pentose sugars on *C. thermocellum* growth rate. (**a**) Effect of increasing concentrations of xylo-oligomers ranging from X_1_-X_4_. (**b**) Effect of various pentose monomers. Average values (n ≥ 9) ± SD are shown.

*C. thermocellum-*mediated xylan hydrolysis has been reported to yield xylobiose as the principal hydrolysis product followed by xylotriose and xylotetraose^10^,^11^,^20^. As such, xylo-oligomers ranging in chain length from X_1_-X_4_ were tested for their effect on growth rate (Fig. 2A; Supplementary Fig. S1). All compounds exerted a concentration dependent inhibitory effect, though the magnitude of the effect was slightly less for X_3_ and X_4_-oligomers than it was for either xylose or xylobiose. For both the mono- and disaccharide, 15 g/L was the inhibitory concentration needed to reduce the growth rate by ~45–50% (IC_50_ = 15 g/L) relative to the uninhibited control. Given the similarity in inhibition, the monomer xylose was chosen as a model substrate for continued investigation.

*C. thermocellum* was next challenged with various other D- and L-isomer pentose sugars (Fig. 2B). All of the pentose sugars tested exerted an inhibitory effect on growth, but the magnitude of inhibition was significantly greater with D-specific isomers relative to L-isomers. Further, while D-xylose was the most potent inhibitor tested, its toxic effects were primarily linked with the D-conformation, which was inhibitory to multiple *C. thermocellum* strains (Supplementary Fig. S1).

As the inhibition seemed both isomer and concentration dependent, we rationalized that xylose and other xylo-oligomers may act as competitive inhibitors for sugar binding proteins and cellobiose uptake. Supporting this hypothesis was the realization that the rate of cellobiose consumption, and not just growth, was inhibited in cultures containing 15 g/L xylose (Supplementary Fig. S1). To assess this potential, competition assays were constructed that varied the molar xylose to cellobiose ratio in the fermentation medium at levels ranging from 0.00–6.84 (Supplementary Table S1). Analyses of the corresponding growth rates offered little support that the basis for inhibition was competition for cellobiose uptake. Rather, growth rates were specific to, and consistent within, the concentration of xylose used independent of the cellobiose concentration. The basis for reduced cellobiose consumption (Supplementary Fig. S1) most likely results from reduced demand, coinciding with inhibited growth, rather than inhibition of cellobiose uptake directly.

### Xylose import and metabolism

Xylose uptake and potential biotransformation were evaluated by conducting metabolomic analyses of mid-exponential phase cultures grown in the absence of xylose or at its IC_50_ concentration using either 15 g/L xylose or 15 g/L D-[U-^13^C] xylose. Comparative analyses of the growth and fermentation end-product profiles between the D-[U-^13^C] xylose and ^12^C-xylose were identical suggesting use of the ^13^C-isotope had no observable effect relative to the light isotope. Very few changes in the metabolomic profiles of the uninhibited and inhibited cultures were observed. Further, the molar distribution of fermentation end-products for strain M1570^19^ also remained relatively unchanged (Supplementary Table S2). The metabolomic changes that were noted, however, involved the appearance of xylulose and xylitol in both light and heavy xylose-containing cultures indicating that *C. thermocellum* can both import and transform xylose (Table 1).

RNA-seq analyses were subsequently conducted to: i) provide insight into the basis for inhibition in strain M1570; and ii) help identify the genes/enzymes responsible for the observed transformations of xylose. From mid-exponential phase cultures, 23 genes were significantly (p-value < 0.05) down-regulated by a factor of two-fold or more, while 37 genes were significantly up-regulated two-fold or more in the xylose inhibited cultures relative to the uninhibited control (Supplementary Table S3). Genes associated with processes such as central metabolism, amino acid and nucleotide biosynthesis, as well as most ribosomal proteins were significantly down-regulated, which is consistent with the slower growth rate observed.

**Figure 3:**
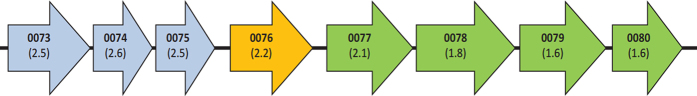
Highly up-regulated 8-gene cluster under xylose-inhibited conditions. Numbers in bold are the numerical suffix of locus tags in *C. thermocellum* DSM 1313 (e.g. 0073 = Clo1313_0073). Numbers in brackets indicate the log_2_ fold change. Annotations are as follows: 0073 – carbohydrate/glycerol kinase (EC 2.7.1.30); 0074 – transketolase subunit A (EC 2.2.1.1); 0075 – transketolase subunit B (EC 2.2.1.1); 0076 – xylitol dehydrogenase (*xdh*); 0077 – ribose binding ABC-transporter; 0078 – ABC-transporter, ATPase; 0079 – ABC-transporter, inner-membrane translocator; 0080 – phosphoglycerate mutase. Colours indicate BioCyc predicted operons^57^.

Eight genes at one locus (Clo1313_0073-0080), predicted to form three distinct operons, were in the top ten most highly up-regulated (3-6 fold) genes in the xylose inhibited cultures (Fig. 3, Supplementary Table S3). Two of these genes (Clo1313_0074-0075) are annotated to encode transketolase subunits. Previous *C. thermocellum* datasets have identified that these transketolase genes are expressed at low levels during uninhibited growth, while another annotated transketolase (Clo1313_0295-0296) is much more abundantly expressed^21^,^22^. We therefore hypothesized that the up-regulation of Clo1313_0074-0075 may be in response to xylose-induced competitive inhibition associated with either transketolase (Tkt) or phosphoribosyl pyrophosphate synthetase (Prs) activity. However, supplementation experiments designed to specifically alleviate Tkt^23^ or Prs^24^ inhibition had no observable effect (Supplementary Fig. S2). The inclusion of nutrient rich medium components, yeast extract, or peptone did stimulate growth rate, but the stimulatory effect was equally observed in both the uninhibited and inhibited cultures (Supplementary Fig. S2). The absence of anabolic inhibition by xylose was consistent with the metabolomic data.

**Figure 4:**
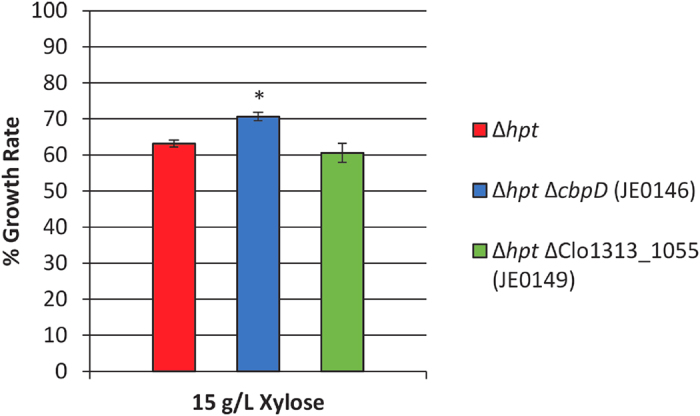
Relief of inhibition by transporter deletion strains. Percent growth rate is relative to the growth rate of that strain under 0 g/L xylose conditions. Error bars represent SE (n = 36). (*) denotes gene deletions that significantly reduced the level of inhibition observed (p-value < 0.01; unpaired two-tailed t-test).

While the ratio of xylose to cellobiose did not seem to affect the observed inhibition (Supplementary Table S1), we rationalized that the mechanism of xylose uptake, particularly if transport is through ATP-dependent mechanisms, might still influence the specific growth rate. Genomic analyses of all *C. thermocellum* genes within the Transporter Classification Database^25^ suggested two probable candidate transporters for xylose uptake. The first was an ABC-type transporter (Clo1313_0077-0079), whose substrate binding protein, Clo1313_0077, is 100% identical at the amino acid level to that of the CbpD transporter in *C. thermocellum* ATCC 27405, which has previously been characterized and its homology to pentose-binding proteins noted^26^. The second (Clo1313_1055) was a major facilitator superfamily protein annotated as an oligosaccharide/H^+^ symporter with homology to arabinose binding proteins. Deletion mutants (Supplementary Fig. S3) of each transporter were constructed in a parental strain lacking a hypoxanthine phosphoribosyltransferase (Δ*hpt*; genetic background used to construct gene deletions)^19^ and the relief of xylose induced inhibition was assessed. Both the parental strain and strain JE0149 (Δ*hpt* ΔClo1313_1055) showed comparable levels of inhibition when comparing their growth rates under 0 g/L and 15 g/L xylose conditions (Fig. 4) indicating that gene deletion had no obvious effect. Deletion of the *cbpD* genes in strain JE0146, however, showed a minor (~8%) but statistically significant relief of inhibition relative to the parental strain indicating that, at least in part, xylose uptake involves an ATP-dependent transporter.

Consistent with the formation of xylulose and xylitol in our metabolomics data was the significant up-regulation of an annotated xylitol dehydrogenase (XDH - Clo1313_0076) within the highly up-regulated 8-gene cluster (Fig. 3). 6X-His-tagged, as well as untagged, recombinant versions of the XDH protein were cloned and expressed in *Escherichia coli*. Despite successful overexpression of the protein, *in vitro* activity when tested with either xylose or xylulose was not observed when assayed aerobically or anaerobically at 55 °C using NADH or NADPH as co-factors (not shown).

Alternatively, a deletion mutant of Clo1313_0076, designated strain JE0148, was constructed (Supplementary Fig. S3). Deletion of the gene did not abolish xylitol formation, but resulted in a reduction in the total and molar yields (mol xylitol : mol cellobiose consumed) of ~41% and ~46% respectively (Table 2) confirming the involvement of the Clo1313_0076 enzyme in xylitol formation. Gene deletion also significantly affected the relative flux distribution of fermentation end-products when cultured in the presence of 15 g/L xylose (Supplementary Table S4). While the relative ethanol and acetate production capabilities were similar between the Δ*hpt* parental strain and JE0148, the latter produced significantly less formate and more lactate than the parental strain.

To determine if xylitol could explain the inhibited growth, it was added to basal growth medium at concentrations up to 20 g/L. Xylitol supplementation had no effect on *C. thermocellum* growth, however (Supplementary Fig. S1), suggesting that extracellular xylitol is not inhibitory.

### AgrD expression and growth inhibition

**Figure 5:**
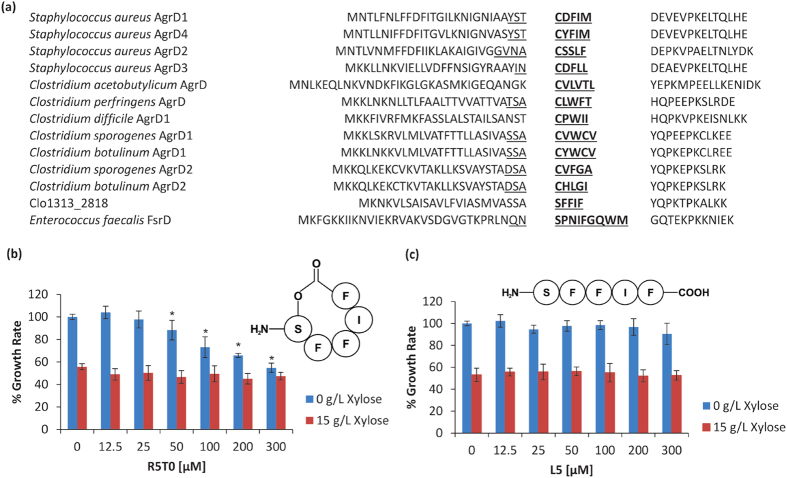
AgrD structure and effect on *C. thermocellum* growth. (**a**) Comparison of AgrD pre-peptide sequences between *C. thermocellum* (Clo1313_2818) and other Firmicutes^28^,^29^,^30^,^58^,^59^,^60^. Underlined residues identify the AIP sequence while those in bold identify the cyclic component. Effect on growth rate when synthetic R5T0 (**b**) and L5 (**c**) peptides are added to culture medium. Percent growth rate is calculated relative to the DMSO only control (0 μM) with 0 g/L xylose. Values are averages (n = 9) ± SD from independent experiments. (*) denotes conditions in which the addition of the synthetic peptide, and not xylose itself, significantly inhibited the growth rate observed (p-value < 0.05; unpaired two-tailed t-test). Figure inserts show the structure of the R5T0 (**b**) and L5 (**c**) peptides tested.

A putative autoinducing pre-peptide encoding gene Clo1313_2818, showing homology to AgrD signalling peptides in other Firmicutes (Fig. 5A), was among the most upregulated (2.3- fold) genes in the xylose inhibited cultures. Mature AgrD are post-translationally modified peptides involved in cell-to-cell signalling processes. While widespread across diverse lineages^27^, including multiple Clostridia^28^,^29^,^30^,^31^, the use of cyclic autoinducing peptides (AIPs) as signalling molecules in any thermophilic bacterium has yet to be reported.

Comparison of the Clo1313_2818 amino acid sequence against reported AgrDs (Fig. 5A) shows a highly conserved proline at position 35 proceeded by a Y30 residue, which is consistent with pre-peptide cleavage sites in other *Clostridium* spp.^28^. SignalP v4.1^32^ predicts cleavage of the leader peptide between A24 and S25, which would ultimately generate a pentapeptide (R5T0) putatively cyclized via a lactone bond (Fig. 5B) between the S25-hydroxyl group and the F29 carboxy-terminus. Targetted LC-MS/MS of the predicted AgrD-like molecule – as informed by the retention time and fragmentation profiles of a synthetic version of the peptide – confirmed both the presence of the proposed cyclic AIP in culture supernatants as well as its sequence and structure (Supplementary Fig. S4).

Synthetic AIP constructs were added to culture medium at various concentrations (Fig. 5B,C) to determine their effect on *C. thermocellum* growth. Addition of the R5T0 peptide reduced the growth rate of *C. thermocellum* cultures in basal medium without xylose supplementation (Fig. 5B). While the concentration of the AIP produced in culture supernatants is unknown, significant inhibition of growth was observed at 50 μM, which is a concentration consistent with previous assessments of cell-to-cell communication responses in other *Clostridium* spp.^28^,^31^,^33^. Under xylose-inhibiting conditions, in which Clo1313_2818 is already up-regulated, AIP supplementation had no additional inhibitory effect on the growth rate. As a control experiment, a linear version (L5) of the pentapeptide was also tested, but had no effect under either condition (Fig. 5C) confirming that the structure of the AIP, and not just the sequence, is essential for eliciting a physiological response.

## Discussion

With respect to industrial applications, the fermentation conditions optimized for an efficient bioprocess can be quite different than those encountered by a microbe within its natural niche. For *C. thermocellum,* which can solubilize xylan, but not ferment the hydrolysis products, the accumulation of pentoses in the absence of C5 co-fermenting strains presents a challenge. Given their abundance in lignocellulose, this study addressed how pentoses impact the organism’s metabolism. In doing so, we gained multiple novel insights into how these saccharides affect fermentation and growth.

We identified that various oligomers of xylose, most notably the principal hydrolysis product xylobiose^10^,^11^,^12^, can significantly inhibit the growth of the bacterium. Also, we confirmed that xylose transport occurs and that it contributes to inhibition. Transport mechanisms capable of importing cellodextrins of varying chain lengths, as well as laminaribiose, have been reported for *C. thermocellum*^26^,^34^,^35^, but xylose uptake has not. The relief of inhibition in the *cbpD* deletion strain (JE0146) suggests that xylose uptake also occurs, at least in part, by ATP-dependent mechanisms. Due to the inability to catabolize the sugar and generate ATP from its breakdown, xylose uptake could impose an energetic penalty on the cell equal to −1 mol of ATP per mol of xylose transported. Consistent with this hypothesis is the observation that strain M1570, whose acetate production capabilities have been reduced by deletion of its phosphotransacetylase gene (Δ*pta*)^19^ resulting in less ATP per acetate formed^21^,^35^, is more inhibited than several acetate-producing strains (Fig. 4, Supplementary Fig. S1).

Xylitol production has previously been observed in *C. thermocellum* fermentations and has been associated with catabolic overflow metabolism^36^,^37^. In those studies however, it was unknown if the formation of xylitol was due to microbially catalyzed processes. Here, we confirm that *C. thermocellum* can convert D-xylose to xylitol presumably at the expense of reduced nicotinamide cofactors. This reduction is important to consider in developing the bacterium into a robust biofuel producing microbe. Fuel molecules are electron-rich and any electron diversion away from the fuel is undesirable. Ethanol yields have been greatly improved in *C. thermocellum* through efforts to disrupt fermentation pathways that compete for electrons^38^,^39^,^40^. Deletion of *xdh* did not significantly affect ethanol production (Table S4); however, its deletion affected formate and lactate production, indicating that XDH can influence redox metabolism when xylose is present.

The transformation of xylose to xylitol may also contribute to the growth inhibition observed. Detoxification of inhibitors that are derived from lignocellulose by processes that consume reducing equivalents has been described^41^,^42^. In *C. thermocellum*, the effects of electron loss through detoxification may be particularly important in strains that lack the ability to synthesize acetate, such as M1570. Maintaining a redox balance during acetate production requires an electron sink, such as proton reduction to H_2_, formate production, or potentially xylose reduction. In the absence of acetate production, use of these sinks takes away electrons that are needed to reduce acetyl-CoA to ethanol or pyruvate to lactate, causing a redox imbalance. Therefore, while xylitol itself may not be inhibitory, its formation may pose a metabolic burden on the cell in terms of lost reducing equivalents.

We have also identified and confirmed that *C. thermocellum* produces an AgrD-type pentapeptide, which to the best of our knowledge, is the first report of mature AgrD production by a thermophile. A distinguishing feature of the mature pentapeptide is that the cyclization occurs via a lactone bond, whereas thiolactone cyclization is more commonly reported in other Clostridia (Fig. 5A). Unlike many other AgrD-like molecules^28^,^43^,^44^, Clo1313_2818 also does not appear to be co-localized within an apparent *agr* operon. Therefore, candidates for a cognate AgrB translocator protein, an AgrC receptor kinase or an AgrA response regulator have not been identified.

Up-regulation of *agrD* and biosynthesis of the AIP does not appear to be exclusively in response to a cell density or “quorum-sensing” effect. Rather, at the time of harvest there was less biomass in the inhibited cultures relative to the uninhibited control (OD_600_ values were 0.49 ± 0.03 and 0.32 ± 0.01 for the 0 g/L and 15 g/L xylose conditions respectively). The high expression level observed at the mid-point of inhibited culture growth is consistent with the high growth-phase expression levels previously reported during switchgrass and *Populus* fermentations^45^. Its increased expression also does not correspond with a transition into stationary phase, which would be consistent with a cell density effect. At the exclusion of these possibilities, induction of *agrD* would, by default, seemingly be linked to an unknown effector molecule or be possibly induced as part of a xylose-induced stress response.

The synthetic AIP clearly had a limiting effect on growth in the absence of pentose sugars (Fig. 5), yet its mode of action and the extent to which the mature pentapeptide explains the xylose-induced reduction in growth has not yet been determined. Small secreted peptides are known to have plurifunctional roles in addition to cell-to-cell communication^46^ and such a role for the Clo1313_2818 mature peptide cannot be discounted. However, we are currently not aware of a functional role for AgrD-type peptides apart from cell-to-cell communication in the Firmicutes. Further studies should provide more insights into the specific purpose of the ArgD system in *C. thermocellum*.

The focus of this research was to study the effect of xylo-oligomers on the fermentation of soluble substrates. The accumulation of non-metabolizable saccharides may also influence continued biomass hydrolysis as has been reported with commercial enzyme cocktails^14^,^15^. Removal of pentose sugars through feedstock pre-treatment^47^, engineering pentose catabolism into *C. thermocellum*, or through co-culturing with pentose-fermenting thermophiles^16^,^48^ would all potentially alleviate inhibition from hemicellulose derived saccharides; but, these strategies also present their own challenges with respect to biological strain and process engineering. It would, however, seemingly be advantageous for xylan removal to occur prior to *C. thermocellum* fermentation or simultaneously alongside hydrolysis and fermentation to avoid pentose accumulation. In any case, investigations into the effect of biomass derived compounds on *C. thermocellum* hydrolysis and metabolism are warranted.

A further complication is that xylose does not just inhibit growth, but also consumes valuable reducing equivalents. Construction of strain JE0148 significantly reduced electron loss to xylose, but led to an increase in lactate, rather than ethanol, formation. Combining this gene deletion with strains recently engineered to have reduced lactate, acetate, formate and hydrogen production capabilities^19^,^38^,^39^,^40^ may further improve the bacterium’s ethanol yields.

Finally, with the exception of *Clostridium acetobutylicum*^28^, the role of cell-to-cell communication in biofuel producers has received relatively little attention. Given the significant regulatory and physiological changes known to be induced by signalling peptides^27^,^49^, increased effort in this area is also warranted. This is particularly true as, like many other Clostridia^28^,^29^, *C. thermocellum* encodes multiple putative signalling peptides within its genome (others include Clo1313_0024 and Clo1313_0946). Elucidation of potential cellular cross-talk mechanisms in *C. thermocellum*, as well as how other axenic culture-induced limitations affect the bacterium’s physiology, will be important in the continued development of the microorganism.

## Materials & Methods

### Bacterial strains, media, and growth

Lab stocks of *C. thermocellum* ATCC 27405, *C. thermocellum* DSM 1313, and *C. thermocellum* M1570^19^ were used for this study (also see Supplementary Table S5). All experiments were performed with strain M1570 unless specifically noted otherwise. Cultures were grown at 55 °C in sealed bottles containing basal Medium for Thermophilic Clostridia (MTC) with 5 g/L cellobiose as previously described^50^ and 100% nitrogen headspace. Details regarding the preparation of amended MTC medium, for use in the inhibition and supplementation studies, can be found in Supplementary Materials & Methods. Inocula for all experiments were grown in basal MTC to an optical density (OD_600_) of 0.45–0.55.

For growth rate studies, 200 μl cultures were grown in 96-well pre-sterilized polystyrene plates in an anaerobic chamber with an inlet gas comprised of 5% H_2_ : 10% CO_2_ : 85% N_2_. OD_600_ measurements were taken every 15 minutes for 24 hours using a Biotek Eon spectrophotometer (BioTek Instruments Inc., Winooski, VT). Growth rates were calculated using the Growth Rates Made Easy software package^51^ and included a minimum of five independent determinations for every biological replicate.

### Fermentation analyses

Sugar consumption and fermentation end-products were measured as previously reported^52^ using a Waters Breeze 2 high-performance liquid chromatography (HPLC) system (Waters Corp., Milford, MA) equipped with an Aminex HPX-87H column (Bio-Rad Laboratories, Hercules, CA) and a refractive index detector. Headspace hydrogen was measured using an Agilent Technologies 6850 Series II gas chromatograph equipped with a Carboxen 1010 plot column and a thermal conductivity detector. Total H_2_ production calculations (headspace and liquid fractions) were done as previously described^53^.

### Transcriptomic analyses

Quadruplicate, 40 mL cultures of *C. thermocellum* were grown in MTC medium containing 15 g/L xylose or with no added xylose. Upon reaching the mid-point of growth as determined via OD_600_, the culture was pelleted by centrifugation at 8,000 x *g* for 5 minutes at 4 °C. Cell pellets was then frozen in liquid nitrogen and stored at −80 °C until RNA could be extracted. RNA isolation and library preparation were done essentially as described^45^ with details provided in Supplementary Materials & Methods.

Raw reads were mapped to the genome [GenBank:CP002416] using CLC Genomics Workbench version 8.0 (CLC bio, Aarhus, Denmark) and the default settings for prokaryote genomes. Counts of uniquely mapped reads were analyzed for differential gene expression by DESeq2^54^. Filtering was applied to identify genes with a false discovery rate <0.05 and a greater than log_2_ transformed differential gene expression of ±1.

### Metabolomics growth and analyses

Universally labelled ^13^C-xylose (D-[U-^13^C] xylose) was purchased from Omicron Biochemicals Inc. (South Bend, IN). For metabolomic analyses, strain M1570 was grown as 12 mL cultures in MTC medium containing 15 g/L xylose, 15 g/L D-[U-^13^C] xylose or in the absence of added xylose. At the mid-point of growth for each respective condition, entire cultures were sacrificed. As xylose uptake was a primary interest of the experiment, cell pellets were washed with ice cold phosphate buffered saline (PBS) to minimize the amount of contaminating xylose or D-[U-^13^C] xylose associated with the cell pellet. In brief, cultures taken from the incubator were pelleted at 8,000 x *g* for five minutes, washed twice with equal volumes of PBS and then snap frozen in liquid nitrogen. Cell pellets were stored at −80 °C until metabolomic analyses could be performed.

Intracellular metabolites were extracted from cell pellets (42–81 mg fresh weight) by sonication for 3 min at 20% amplitude, 10 s on and 10 s off, with the pellets kept on ice. Extracts were then centrifuged for 20 min at 4,500 rpm and stored at −20 °C until analysis. Extracts were analyzed as trimethylsilyl derivatives by gas chromatography-mass spectrometry (GC-MS), using electron impact ionization (70 eV), as described elsewhere^37^. Included modifications were the use of 500 μl of silylation reagents and that samples were injected two days after derivatization into an Agilent 5975 C gas chromatograph-mass spectrometer using 2.5 full spectrum scans per second (50–650 Da). Biological replicates (in quadruplicate) were used for each condition with the ^12^C- and ^13^C-xylose samples compared to the untreated control. GC-MS analyses of our ^12^C- and ^13^C-xylose stock solutions confirmed that they did not contain either xylulose or xylitol (not shown).

### Targetted LC-MS/MS for the detection of the putative cyclic AIP

Supernatant samples collected from *C. thermocellum* cultures grown with or without 15 g/L xylose were either concentrated or processed via solid-phase extraction (SPE) to enrich for the AIP. Regardless of the enrichment method, supernatant was first precleared via centrifugation (10 min at 4,500 × g) and filtered through a 5 kDa MWCO spin column (Vivaspin 20; GE Healthcare; Pittsburg, PA) to remove larger proteins and polypeptides. Two mL of precleared supernatant were concentrated to 150 μL by SpeedVac centrifugation and adjusted to 10% acetonitrile and 0.5% formic acid prior to sample loading. To enrich for the AIP via SPE, 5 mL of precleared supernatant was passed through an equilibrated C18 Sep-Pak Lite SPE cartridge (Waters Corp; as per their published protocol), washed twice with 10 mL solvent A (95% LC-MS-grade H_2_O, 5% LC-MS-grade acetonitrile, 0.1% formic acid) and eluted with 3 mL of 100% acetonitrile into three 1 mL aliquots. Enriched aliquots were then concentrated via SpeedVac centrifugation, combined (~100 μL total), and acidified to 0.1% formic acid prior to sample loading.

Sample loads representing 5% (SPE) to 20% (concentrate) of unadulterated supernatant were loaded onto a 150-micron ID back column with 5 cm C18 resin (5 micron Kinetex; Phenomenex, Torrance, CA). After washing the loaded back column with solvent A for 10 min, it was placed in-line with an in-house-pulled nanospray emitter packed with 15 cm C18 resin and analyzed by LC-MS/MS over a 30 min organic gradient to 100% solvent B (5% H_2_O, 95% acetonitrile, 0.1% formic acid). Targetted monitoring of the predicted cyclical AIP (642.3292 m/z) was performed using an Orbitrap Pro mass spectrometer (Thermo Fisher Scientific, West Palm Beach, FL). Briefly, a m/z range of 642.33 ± 1.1 was continuously isolated and fragmented by successive CID (35% energy) and HCD (30% energy) events over the course of the separation. Fragment ions resulting from either CID or HCD were passed to the Orbitrap mass analyzer for high-resolution measurements with parts-per-million (ppm) accuracy. Extracted-ion chromatograms (XIC; mass tolerance = 5 ppm) for specific fragment ions mapping to the predicted AIP, as gleaned from a preliminary CID and HCD measurements of the synthetic AIP, were monitored using Xcalibur Qual Browser v.2.2. XIC peaks at the same retention time indicate the presence of the AIP in culture supernatants.

### Synthetic AIP and cell response

The R5T0 cyclic synthetic peptide was ordered from Peptide Protein Research Ltd. (Fareham, United Kingdom), while the L5 linear peptide was supplied by LifeTein LLC (Somerset, NJ). The lyophilized peptides were reconstituted in filter sterilized DMSO, diluted to the desired concentrations and then added to MTC medium with or without 15 g/L xylose. The final concentration of DMSO for all concentrations was 0.8% (v/v). Growth assays were conducted as 200 μl cultures using 96-well plates and growth rates were determined as described above.

### Vector construction, strain engineering and mutant strain analyses

Standard methods for vector construction via Gibson’assembly^55^ and gene deletion in *C. thermocellum*^56^ were followed and are described in detail in Supplementary Materials & Methods (also see Supplementary Table S5). For strain JE0146 (Δ*hpt* Δ*cbpD*) and JE0149 (Δ*hpt* ΔClo1313_1055), the effect of gene deletion was assessed through growth rate analyses under 0 g/L or 15 g/L xylose conditions as described above. For the *xdh* gene deletion, comparative 72-hour fermentations were performed between the Δ*hpt* parental strain and strain JE0148 (Δ*hpt* ΔClo1313_0076) in medium supplemented with 15 g/L cellobiose, 15 g/L xylose and an additional 5 g/L MOPS buffer (final concentration is 10 g/L). Xylitol and end-product analyses were performed via HPLC as described above.

## Additional Information

**Accession codes:** Raw RNA-Seq data have been deposited in NCBI Sequence Read Archive under accession SRP070709 and gene expression data under NCBI GEO accession GSE78219.

**How to cite this article:** Verbeke, T. J. *et al*. Pentose sugars inhibit metabolism and increase expression of an AgrD-type cyclic pentapeptide in *Clostridium thermocellum. Sci. Rep.* **7**, 43355; doi: 10.1038/srep43355 (2017).

**Publisher's note:** Springer Nature remains neutral with regard to jurisdictional claims in published maps and institutional affiliations.

## Supplementary Material

Supplementary Information

## Figures and Tables

**Table 1:** Xylose derived metabolites in *C. thermocellum* M1570 cell pellets grown under varying conditions.

Metabolite	μg of metabolite/sorbitol equivalent ± SEM		
	0 g/L Xylose	15 g/L Xylose	15 g/L D-[U-^13^C]-Xylose
Xylose	ND^a^	87.12 ± 16.19	ND
Xylulose	ND	0.99 ± 0.15	ND
Xylitol	ND	0.36 ± 0.03	ND
^13^C-Xylose	ND	ND	127.73 ± 19.16
^13^C-Xylulose	ND	ND	0.63 ± 0.10
^13^C-Xylitol	ND	ND	0.10 ± 0.01

^a^ND denotes that the metabolite was not detected.

**Table 2:** Xylitol production in a Δ*hpt* parental strain and strain JE0148.

Strain	Xylitol Produced (mM)	Cellobiose Consumed (mM)	Xylitol(μM)/Cellobiose (mM)^a^
Δ*hpt*	2.74 ± 0.13	14.12 ± 2.21	230.77 ± 41.87
JE0148	1.62 ± 0.10	13.82 ± 2.19	123.81 ± 20.80

Average values ± SD from four independent experiments (n = 16). ^a^p-value < 0.05 (unpaired two-tailed t-test).
