# Corrigendum: Pentose sugars inhibit metabolism and increase expression of an AgrD-type cyclic pentapeptide in *Clostridium thermocellum*

**DOI:** 10.1038/srep46875

**Published:** 2017-07-27

**Authors:** Tobin J. Verbeke, Richard J. Giannone, Dawn M. Klingeman, Nancy L. Engle, Thomas Rydzak, Adam M. Guss, Timothy J. Tschaplinski, Steven D. Brown, Robert L. Hettich, James G. Elkins

Scientific Reports 7: Article number: 43355; 10.1038/srep43355 published online: 02 23 2017; updated: 07 27 2017.

This Article contains typographical errors in the Additional Information section under subheading ‘Accession codes’.

“Raw RNA-Seq data have been deposited in NCBI Sequence Read Archive under accession SRP070709 and gene expression data under NCBI GEO accession GSE78219”.

should read:

“Raw RNA-Seq data have been deposited in NCBI Sequence Read Archive under accession SRP066062 and gene expression data under NCBI GEO accession GSE74918”.

