# Supplementary material for: Pentose sugars inhibit metabolism and increase expression of an AgrD-type cyclic pentapeptide in Clostridium thermocellum
Source: Sci Rep. 2017 Feb 23;7:43355. doi: 10.1038/srep43355 (PMC5322536; doi:10.1038/srep43355)
Supplement: Supplementary Information [file srep43355-s1.pdf]

Supplementary Information

Pentose sugars inhibit metabolism and increase expression of an AgrD-type cyclic pentapeptide in *Clostridium thermocellum*

T. J. Verbeke,[1,2†] R. J. Giannone,[1,3] D. M. Klingeman,[1,2] N. L. Engle,[1,2] T. Rydzak,[1,2] A. M. Guss,[1,2] T. J. Tschaplinski,[1,2] S. D. Brown,[1,2] R. L. Hettich,[1,3] and J. G. Elkins[1,2*]

[1]BioEnergy Science Center, Oak Ridge National Laboratory, 1 Bethel Valley Road, Oak Ridge, TN 37831

[2]Biosciences Division, Oak Ridge National Laboratory, 1 Bethel Valley Road, Oak Ridge, TN 37831

[3]Chemical Sciences Division, Oak Ridge National Laboratory, 1 Bethel Valley Road, Oak Ridge, TN 37831

[†]Current address: Department of Biological Sciences, University of Calgary, Calgary, AB T2N 1N4, Canada

*email: elkinsjg@ornl.gov

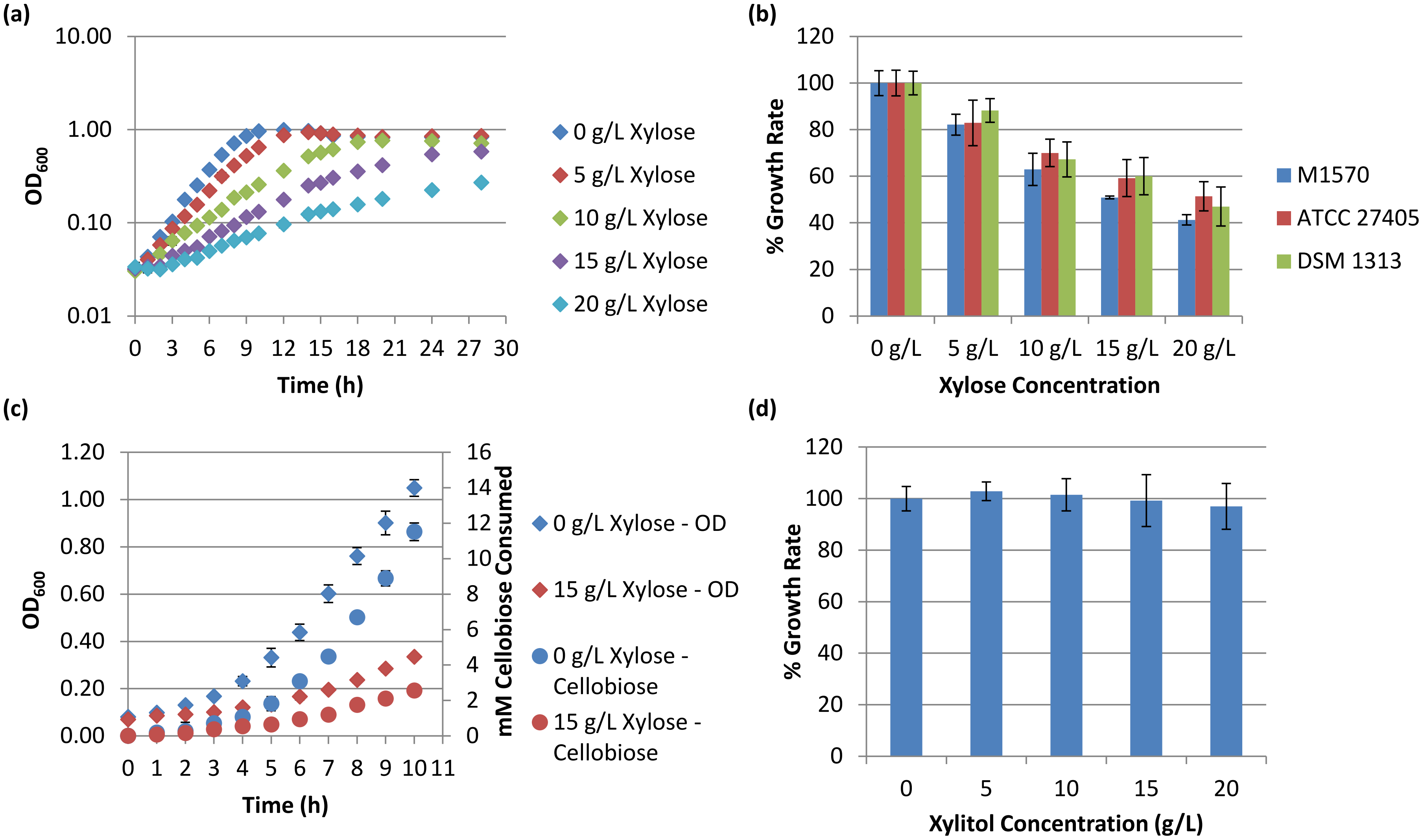

Figure S1. Inhibitory effect of pentoses on growth. (a) *C. thermocellum* M1570 growth at various xylose concentrations (n = 6). (b) Effect of xylose on the growth rate of various *C. thermocellum* strains. Percent growth rate is calculated relative to the same strain in the absence of added xylose (n = 9). (c) Cellobiose consumption and cell growth of *C. thermocellum* M1570 (n = 3). (d) Effect of extracellular xylitol on *C. thermocellum* M1570 growth rate (n = 6). For all figures, values are averages and error bars represent SD.

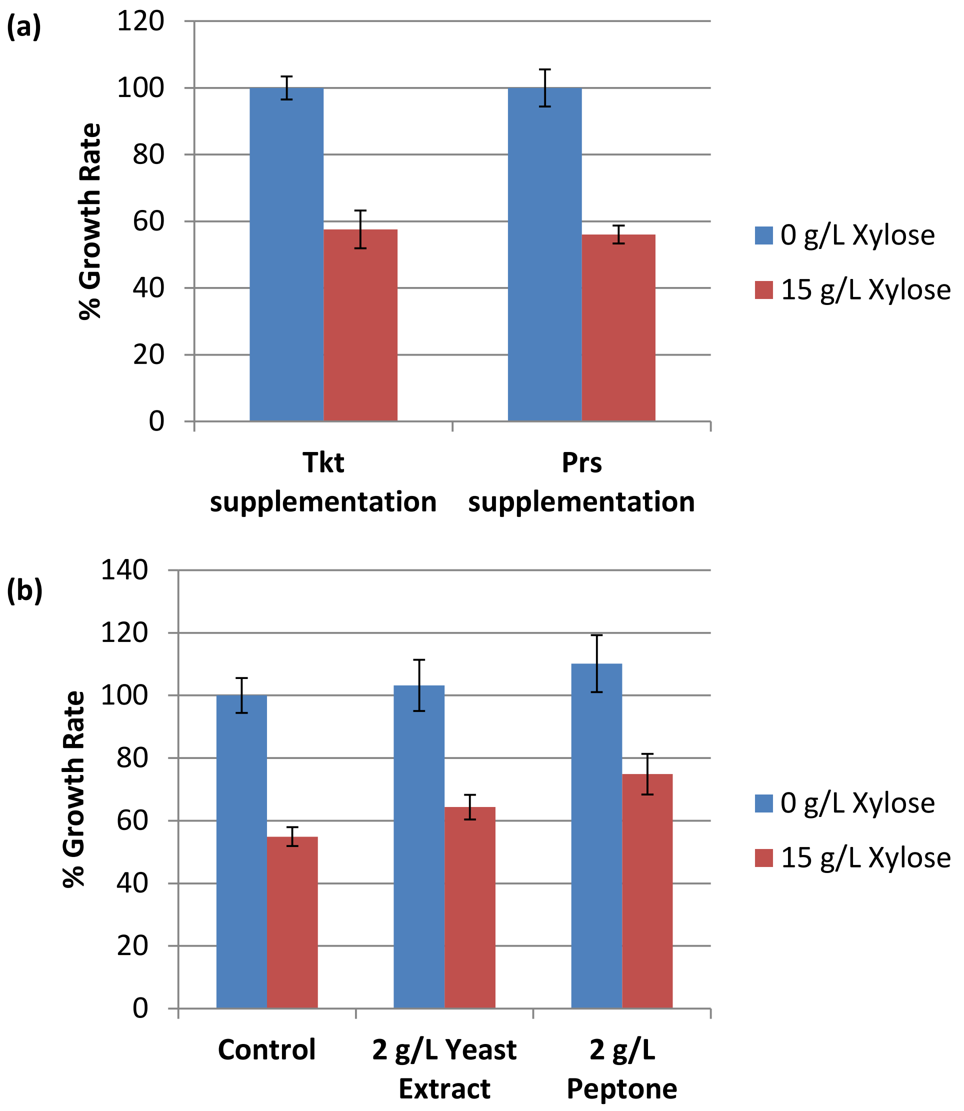

Figure S2. Effect of medium supplementation as a strategy to alleviate inhibition. (a) Alleviation of inhibition by Tkt or Prs supplementation. Percent inhibition is calculated relative to the observed growth rate for the strain with supplementation, but in the absence of xylose (n = 6). (b) Relief of inhibition by yeast extract or tryptone (n = 6). For both figures, error bars represent SD.

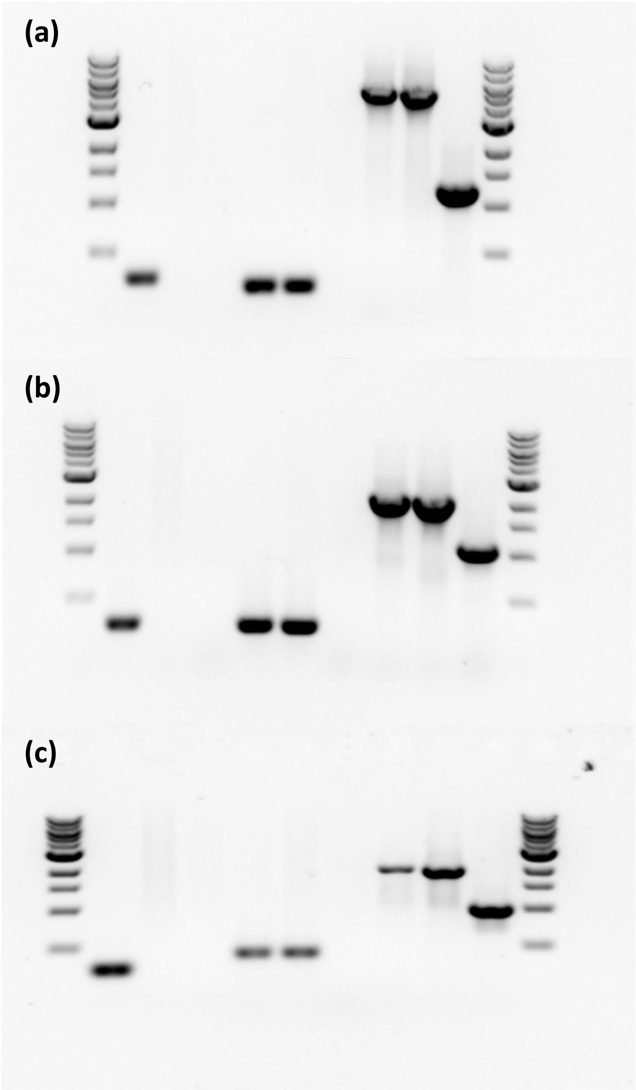

Figure S3. PCR confirmation of gene deletions in *C. thermocellum*. For all gels, PCR products were generated from three strains of *C. thermocellum* including: DSM 1313^{WT} (lanes 2, 5, 8), the Δ*hpt* parental strain (lanes 3, 6, 9) and the transformant (lanes 4, 7, 10). A 1-Kb ladder (New England Biolabs) is in lanes 1 and 11. (a) PCR analyses of JE0146 (Δ*hpt* Δ*cbpD*). Lanes 2-4: PCR targetting the *hpt* gene. Lanes 5-7: PCR targetting a region internal to Clo1313_0079. Lanes 8-10: PCR targetting regions flanking the Clo1313_0077-0079 region. (b) PCR analyses of JE0149 (Δ*hpt* ΔClo1313_1055). Lanes 2-4: PCR targetting the *hpt* gene. Lanes 5-7: PCR targetting a region internal to Clo1313_1055. Lanes 8-10: PCR targetting regions flanking the Clo1313_1055 region. (c) PCR analyses of JE0148 (Δ*hpt* ΔClo1313_0076). Lanes 2-4: PCR targetting the *hpt* gene. Lanes 5-7: PCR targetting a region internal to Clo1313_0076. Lanes 8-10: PCR targetting regions flanking the Clo1313_0076 region.

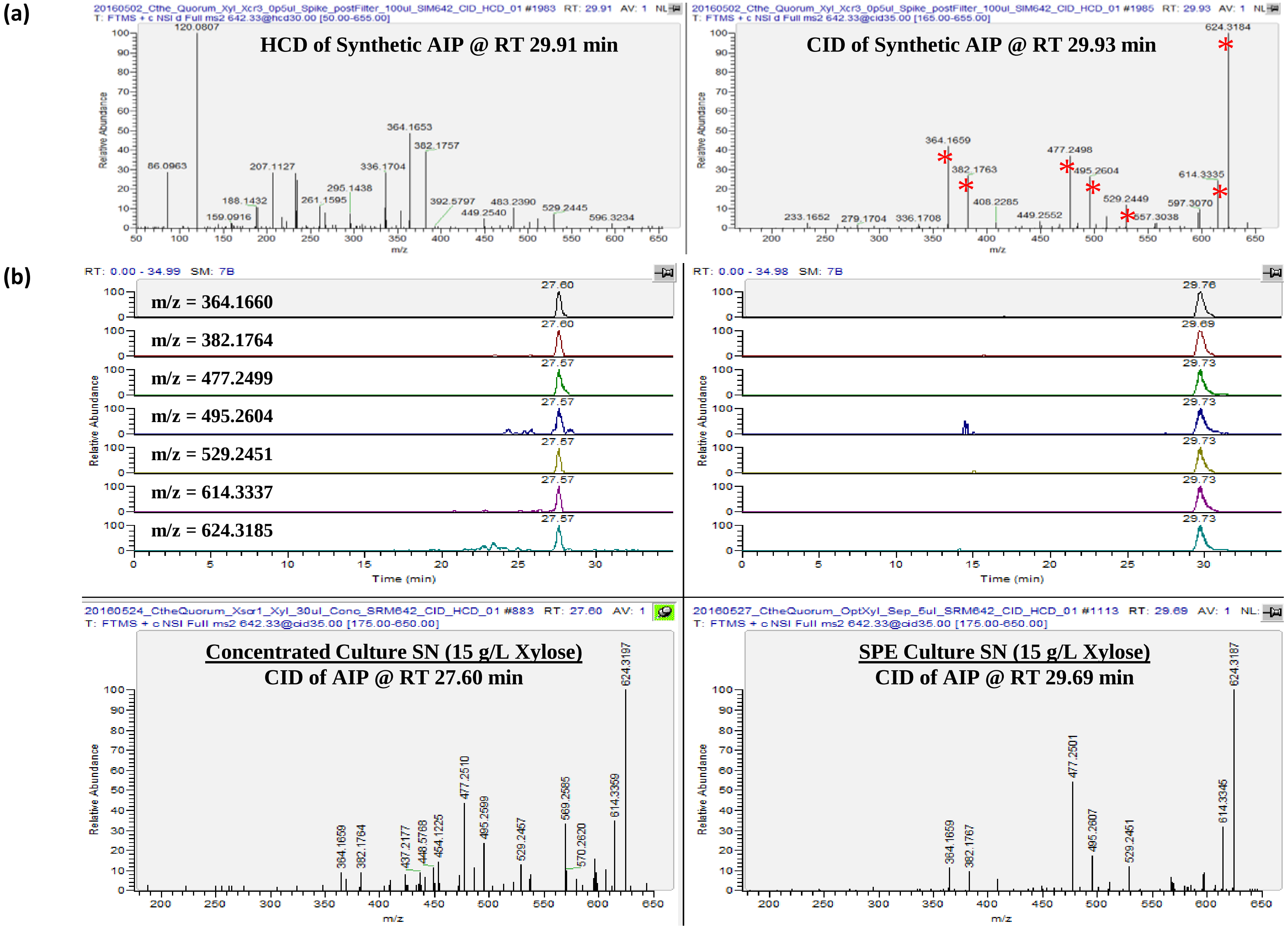

Figure S4. Targetted LC-MS of putative AIP confirms its presence in *C. thermocellum* culture supernatants supplemented with 15 g/L xylose. (a) Retention time and MS/MS profile of the synthetic R5T0, fragmented via HCD (left) or CID (right). Red asterisks denote specific CID fragment peaks used to identify the cyclic AIP in culture supernatants and correspond to the 7 XIC panels (5 ppm mass tolerance) presented in (b). Fragmentation profiles (CID only) at XIC apices match those gleaned from the synthetic AIP in both raw, concentrated supernatants (b; left), and SPE-processed supernatants (b; right), with the latter exhibiting a much cleaner profile, as expected. SN = supernatant.

TABLE S1. Specific growth rates (h^{-1}) of *C. thermocellum* M1570 cultures containing varying ratios of xylose to cellobiose.

Xylose Concentration (g/L)	Cellobiose Concentration (g/L)			
	5	10	15	20
0	0.45 ± 0.02	0.43 ± 0.01	0.43 ± 0.01	0.44 ± 0.03
5	0.37 ± 0.02	0.34 ± 0.02	0.36 ± 0.01	0.35 ± 0.01
10	0.28 ± 0.03	0.29 ± 0.00	0.30 ± 0.02	0.29 ± 0.01
15	0.23 ± 0.00	0.23 ± 0.00	0.23 ± 0.00	0.22 ± 0.00

Average values (n ≥ 6) ± SD from independent experiments.

TABLE S2. Major fermentation end-products in *C. thermocellum* M1570 cultures containing 0 g/L or 15 g/L xylose[a].

	Fermentation Products (mol mol^{-1} hexose consumed)								
Xylose	Glucose	Acetate	Formate	Lactate	Ethanol	H_2	CO_2[b]	O/R[c]	Carbon Recovery %
0 g/L	0.00 ± 0.00	0.01 ± 0.00	0.13 ± 0.01	0.00 ± 0.00	1.31 ± 0.09	0.04 ± 0.01	1.18 ± 0.07	0.95 ± 0.01	65.38 ± 5.29
15 g/L	0.00 ± 0.00	0.01 ± 0.00	0.12 ± 0.02	0.01 ± 0.01	1.25 ± 0.10	0.06 ± 0.01	1.14 ± 0.09	0.94 ± 0.00	64.24 ± 4.43

[a]Cultures grown in MTC medium with 5 g/L cellobiose. End-products measured from culture supernatants collected 24 hours post-inoculation.

[b]CO_2 calculated based on the formula: CO_2 = Ethanol + Acetate – Formate

[c]Ratio of oxidized end-products to reduced end-products

TABLE S3. Differential RNA-seq analyses of C. thermocellum M1570 cultures grown under 15 g/L xylose conditions vs. 0 g/L xylose conditions

*Table provided at the end of this file

TABLE S4. Molar end-product ratios for a Δ*hpt* parental strain and strain JE0148.

	Fermentation Products (mol mol^{-1} cellobiose consumed)				
Strain	Acetate	Ethanol	Formate	Lactate	Xylitol
Δ*hpt*	0.65 ± 0.11	0.84 ± 0.16	**0.52 ± 0.05**	**0.28 ± 0.04**	**0.23 ± 0.04**
JE0148	0.66 ± 0.10	0.86 ± 0.17	**0.41 ± 0.06**	**0.54 ± 0.06**	**0.12 ± 0.02**

Average values ± SD from four independent experiments. Values in **BOLD** indicate significant differences in end-product formation between the strains (p-value < 0.05; unpaired two-tailed t-test; n = 16).

Table S5 Strains, plasmids and primers used in this study.

Plasmid, Strain or Primer	Relevant characteristics/genotype	Source
Plasmid		
pNJ22	Backbone vector	Rydzak *et al.* (under review)
pNJ22::cbpD_del	Clo1313_0077-0079 deletion vector	This study
pNJ22:1055_del	Clo1313_1055 deletion vector	This study
pNJ22:0076_del	Clo1313_0076 deletion vector	This study
C. thermocellum Strain		
DSM 1313	Wild-type	DSMZ[a]
ATCC 27405	Wild-type	ATCC[b]
Δ*hpt* parental	DSM 1313; Δ*hpt*	(1)
JE0146	DSM 1313; Δ*hpt*; Δ*cbpD* (Clo1313_0077-0079)	This study
JE0148	DSM 1313; Δ*hpt*; ΔClo1313_0076	This study
JE0149	DSM 1313; Δ*hpt*; ΔClo1313_1055	This study
Primer	Sequence 5’ → 3’	Purpose
CBPD_UP F	agataaaatatttctagggccggccgGAATGTGCGGGAAGCCGT	Construction of pNJ22::cbpD_del
CBPD_UP R	ataaatcttcatgACTTTTATACACCTCACATAATTATTTTCCTG	Construction of pNJ22::cbpD_del
CBPD_DOWN F	ggtgtataaaagtCATGAAGATTTATTTAATCCG	Construction of pNJ22::cbpD_del
CBPD_DOWN R	attaatttttaaGCAGAGAAACAACTACGG	Construction of pNJ22::cbpD_del
CBPD_CAT/HPT F	gttgtttctctgcTTAAAAAATTAATTATTTTTATCTAAACTATTG	Construction of pNJ22::cbpD_del
CBPD_CAT/HPT R	acattgggaagtaTTATGAATACATTTCAGGTTTCAAAAC	Construction of pNJ22::cbpD_del
CBPD_INT F	aatgtattcataaTACTTCCCAATGTTGTTATTATC	Construction of pNJ22::cbpD_del
CBPD_INT R	acgttcttagtcacgactcctcgaggTTATTTCCTAAAGATTATCCCTTG	Construction of pNJ22::cbpD_del
CBPD_Vint F	AACCGGGCGCTATATTTAC	Verification of Δ*cbpD* (strain JE0146)
CBPD_Vint R	AGACCACCGATGATAGTACCTAC	Verification of Δ*cbpD* (strain JE0146)
CBPD_Vtar F	GTAATAGCTCTTCTTGTTACAACAATGC	Verification of Δ*cbpD* (strain JE0146)
CBPD_Vtar R	GGTCCATACCATTCATAATCTACTGTAA	Verification of Δ*cbpD* (strain JE0146)
CBPD_Vflank F	AGAGGGAAGACATTGTGGAGTTTG	Verification of Δ*cbpD* (strain JE0146)
CBPD_Vflank R	GTGGATATTCAAAACCCAAACAGC	Verification of Δ*cbpD* (strain JE0146)
Clo1313_0076_UP F	agataaaatatttctagggccggccgAATTGTGATGTAGAGATAGGAAAAG	Construction of pNJ22:0076_del

Clo1313_0076_UP R	tgccttcgggcatTCTTTTACCTCCGTCTGG	Construction of pNJ22:0076_del
Clo1313_0076_DOWN F	cggaggtaaaagaATGCCCGAAGGCAATACG	Construction of pNJ22:0076_del
Clo1313_0076_DOWN R	attaatttttaaCTCTTTTACAGCAGCATCAATACC	Construction of pNJ22:0076_del
Clo1313_0076_CAT/HPT F	tgctgtaaaagagTTAAAAAATTAATTATTTTTTATCTAAACTATTG	Construction of pNJ22:0076_del
Clo1313_0076_CAT/HPT R	actgtgcacaaacTTATGAATACATTTCAGGTTTCAAAAC	Construction of pNJ22:0076_del
Clo1313_0076_INT F	aatgtattcataaGTTTGTGCACAGTTATGGC	Construction of pNJ22:0076_del
Clo1313_0076_INT R	acgttcttagtcacgactcctcgaggTTAAAATTTAATAACAGATTTTATAACTTC	Construction of pNJ22:0076_del
Clo1313_0076_Vint F	GAGTAAGTGAAATTTATGTTACTGATG	Verification of Δ0076 (JE0148)
Clo1313_0076_Vint R	TTATAACTTCATCCTTGTGATTTG	Verification of Δ0076 (JE0148)
Clo1313_0076_Vtar F	TGGGTAGAACGCGATATTC	Verification of Δ0076 (JE0148)
Clo1313_0076_Vtar R	ACTATGAACGCAGTACTCGG	Verification of Δ0076 (JE0148)
Clo1313_0076_Vflank F	GTAATATACAATGAAAATTGTGATGTAG	Verification of Δ0076 (JE0148)
Clo1313_0076_Vflank R	CCATACCATTCATAATCTACTGTAAC	Verification of Δ0076 (JE0148)
Clo1313_1055_UP F	agataaaatatttctagggccggccgGGAAATGAATCTTTTTGAATATG	Construction of pNJ22:1055_del
Clo1313_1055_UP R	aaccggataaaaaCCTGCTCCTTAACCTTTTT	Construction of pNJ22:1055_del
Clo1313_1055_Down F	gttaaggagcaggTTTTTATCCGGTTTTTATATCTTTTTG	Construction of pNJ22:1055_del
Clo1313_1055_Down R	attaatttttaaTTGTGACAATAAGATTGTTTATTCC	Construction of pNJ22:1055_del
Clo1313_1055_CAT/HPT F	cttattgtcacaaTTAAAAAATTAATTATTTTTTATCTAAACTATTG	Construction of pNJ22:1055_del
Clo1313_1055_CAT/HPT R	atcggaaagaaagTTATGAATACATTTCAGGTTTCAAAAC	Construction of pNJ22:1055_del
Clo1313_1055_INT F	aatgtattcataaCTTTCTTTCCGATTTACTATAAAAAC	Construction of pNJ22:1055_del
Clo1313_1055_INT R	acgttcttagtcacgactcctcgaggCTATTGTCCTGTAATTTTTTGTC	Construction of pNJ22:1055_del
Clo1313_1055_Vint F	GCGAGTGAAATAATCTTCCTTGT	Verification of Δ1055 (strain JE0149)
Clo1313_1055_Vint R	CGATTAAATCACTTATCACACCG	Verification of Δ1055 (strain JE0149)
Clo1313_1055_Vtar F	TCTTAAACGTTCAGAAAGATACCC	Verification of Δ1055 (strain JE0149)
Clo1313_1055_Vtar R	CCAATGTAATCGCATCACCTAT	Verification of Δ1055 (strain JE0149)
Clo1313_1055_Vflank F	AAGTTGAAAGGGACCAGTCATTT	Verification of Δ1055 (strain JE0149)
Clo1313_1055_Vflank R	GAGCAAAGAAGCTTATGTGGTCTT	Verification of Δ1055 (strain JE0149)
hpt_F	ATTCCACCAAATCATCGGGG	Amplification of region internal to *hpt*
hpt_R	AAACAGACGAATCCAGCACG	Amplification of region internal to *hpt*

[a]Deutsche Sammlung von Mikroorganismen und Zellkulturen GmbH, Germany

[b]American Type Culture Collection, Rockville, MD

(1) Argyros, D.A. *et al*. High ethanol titers from cellulose by using metabolically engineered thermophilic anaerobic microbes. *Appl Environ Microbiol*. **77,** 8288-8294, (2011).

Note: Lower case letters indicate regions of overlap for Gibson assembly; Upper case letters indicate regions of homology for amplification

Supplemental Materials & Methods

Inhibition and supplementation studies. For the pentose inhibition studies, MTC medium was supplemented with one of D-xylose, D-ribose, L-arabinose, L-xylose, D-xylobiose, D-xylotriose or D-xylotetraose prior to pH adjustment. Xylobiose was purchased from TCI America (Portland, OR), while xylotriose and xylotetraose were purchased from Megazyme (Bray, Ireland). The chemical purity of each xylo-oligomer was >98%. Beechwood xylan (X4252 Sigma-Aldrich, St. Louis, MO) was used as a representative xylan for testing its inhibitory effect on *C. thermocellum* metabolism. To avoid xylan hydrolysis due to autoclaving and selection for only small xylo-oligomers using filter sterilization, Beechwood xylan was not sterilized prior to its inclusion in MTC medium. Rather, desired amounts of xylan were added to pre-sterilized Balch tubes and then sterile MTC medium was added prior to gassing : degassing. Changes in the medium pH due to the presence of acetate groups in xylan containing medium were accounted for by making moderately alkaline MTC medium such that, at the xylan concentrations tested, the starting pH for all fermentations ranged from 7.0 – 7.3. Since the xylan was not sterilized, culture purity was inferred if cellobiose consumption, production of fermentation end-products (lactate, acetate, formate, ethanol) and a change in medium pH was not observed in replicate uninoculated controls (6 controls per experiment). The amount of xylan used was adjusted to ensure equivalent pentose loadings between the xylan or xylose cultures assuming a composition of 90% xylose-equivalent residues in the xylan as listed by the vendor.

Supplementation experiments were conducted by making MTC medium as described with additional vitamins and amino acids. Medium for transketolase (Tkt) supplementation contained, in addition to basal MTC, 400 μM each of L-tryptophan, L-phenylalanine, and L-tyrosine alongside 100 μM each of vitamin B_6, *p*-aminobenzoic acid, *p*-hydroxybenzoate, and

2,3-dihydroxybenzoate. For phosphoribosyl-pyrophosphate synthetase (Prs) supplementation, the basal medium was provided with an additional 400 μM each of hypoxanthine, uracil, L-histidine, and L-tryptophan in addition to 100 μM each of nicotinic acid and thiamine. Yeast extract or peptone was additionally used at 2 g/L for separate supplementation experiments.

RNA isolation and library preparation. Pelleted cells were resuspended in 2 mL of TRIzol Reagent (Invitrogen, Carlsbad, CA) and 1 mL of the TRIzol/cell mixture was lysed via bead beating with 0.8 g of 0.1 mm glass beads (BioSpec Products, Bartlesville, OK, USA) for 3 x 20 seconds each at 6,500 rpm in a Precellys 24 high-throughput tissue homogenizer (Bertin Technologies, Montigny-le-Bretonneux, France). The RNA from each cell lysate was purified, DNaseI-treated, and quantity and quality assessed, as previously described[1]. RNA with an integrity number >8 was depleted of rRNA using Ribo-Zero rRNA Removal Kit for bacteria (Epicentre-Illumina, San Diego, USA) following the manufacturer's protocol. The depleted sample was then purified on a RNA Clean & Concentrator-5 (Zymo Research, Irvine, CA).

RNA-Seq library preparation was performed using the depleted RNA and the Epicentre ScriptSeq v2 RNA-Seq Library Preparation Kit (Epicentre-Illumina, San Diego, CA) following the manufacturer's protocol (EPILIT329 Rev.C). Agencount AMPure beads (Beckman Coulter, Indianapolis, IN) were used to purify the cDNA, and unique indexes were added during 13 cycles of library amplification. The final RNA-Seq libraries were purified with Agencount AMPure beads (Beckman Coulter) and quantified with a Qubit fluorometer (Life Technologies, Carlsbad, CA). The library quality was assessed on a Bioanalyzer DNA 7500 DNA Chip (Agilent, Santa Clara), and samples were pooled and diluted. Two paired end sequencing runs were completed (2 x 251) on an Illumina MiSeq Instrument (San Diego, CA).

Vector construction and strain engineering.

Deletion vectors were assembled using Gibson's method essentially as described[2] using pNJ22 as the backbone vector (Rydzak *et al*., under review). DpnI digested PCR fragments targeting the regions of homology used for gene deletion (500 bp upstream of CDS; 500 bp downstream of CDS; and the terminal 500 bp of CDS) were ligated alongside a 1388 bp fragment encoding a chloramphenicol acetyltransferase and a hypoxanthine phosphoribosyltransferase under control of a *C. thermocellum* glyceraldehyde-3-phosphate promoter into EcoRI digested pNJ22 plasmid for vector construction. Standard methods for gene deletion in *C. thermocellum* were followed[3] using plasmid isolated from *dcm*$^-$ *Escherichia coli* and electroporated into a Δ*hpt C. thermocellum* parental strain (Supplementary Table S5). After multiple rounds of selection[3], single colony isolates were picked and gene deletions confirmed via PCR using appropriate primers (Supplementary Table S5).

References for Supplemental Materials & Methods

1. Yang, S. *et al*. *Clostridium thermocellum* ATCC27405 transcriptomic, metabolomic and proteomic profiles after ethanol stress. *BMC Genomics*. **13,** 336; 10.1186/1471-2164-13-336 (2012).
2. Gibson, D.G. Enzymatic assembly of overlapping DNA fragments. *Methods Enzymol*. **498,** 349-361 (2011).
3. Olson, D.G. & Lynd, L.R. Transformation of *Clostridium thermocellum* by electroporation. *Methods Enzymol*. **510,** 317-330 (2012).

TABLE S3 Differential RNA-seq analyses of *C. thermocellum* M1570 cultures grown under 15 g/L xylose conditions vs. 0 g/L xylose conditions			
**Locus Tag**	**Gene Product Name**	**Log_2 transformed ratio (15 g/L Xylose -0 g/L Xylose)**	**Adusted P-Value**
Clo1313_0001	chromosomal replication initiator protein DnaA	-0.2	4.20E-02
Clo1313_0002	DNA polymerase III, beta subunit (EC 2.7.7.7)	-0.4	1.66E-04
Clo1313_0003	S4 domain protein YaaA	-0.4	1.19E-01
Clo1313_0004	DNA replication and repair protein RecF	-0.3	6.00E-02
Clo1313_0005	hypothetical protein	-0.2	1.11E-01
Clo1313_0006	DNA gyrase, B subunit	-0.5	1.12E-07
Clo1313_0007	chromosome segregation ATPase	-0.2	7.16E-02
Clo1313_0008	parB-like partition protein	-0.3	1.79E-02
Clo1313_0009	hypothetical protein	0.0	8.61E-01
Clo1313_0010	TPR repeat-containing protein	-0.2	1.31E-01
Clo1313_0011	seryl-tRNA synthetase (EC 6.1.1.11)	-0.6	2.77E-14
Clo1313_0012	major facilitator superfamily MFS_1	0.2	1.50E-01
Clo1313_0013	copper amine oxidase-like domain-containing protein	-0.4	9.42E-05
Clo1313_0014	S-layer domain-containing protein	0.7	7.85E-11
Clo1313_0015	hypothetical protein	0.1	8.54E-01
Clo1313_0016	VanW family protein	0.0	7.97E-01
Clo1313_0017	gamma-D-glutamyl-{L}-meso-diaminopimelate peptidase I (EC:3.4.19.11). Metallo peptidase. MEROPS family M14C	0.0	9.54E-01
Clo1313_0018	hypothetical protein	-0.1	8.49E-01
Clo1313_0019	pro-sigmaK processing inhibitor BofA	-0.2	5.29E-01
Clo1313_0020	pyruvate/ketoisovalerate oxidoreductase, gamma subunit	-0.9	4.53E-22
Clo1313_0021	pyruvate ferredoxin/flavodoxin oxidoreductase, delta subunit	-0.7	1.85E-10
Clo1313_0022	pyruvate flavodoxin/ferredoxin oxidoreductase domain protein	-0.8	2.97E-22
Clo1313_0023	thiamine pyrophosphate TPP-binding domain-containing protein	-1.0	1.88E-48
Clo1313_0024	putative lipoprotein	-0.1	5.92E-01
Clo1313_0025	hypothetical protein	-0.4	2.68E-01
Clo1313_0026	helix-turn-helix domain protein	0.0	9.22E-01
Clo1313_0027	hypothetical protein	0.2	5.80E-01
Clo1313_0028	glycosyl transferase family 39	0.1	8.97E-01
Clo1313_0029	spore coat protein, CotS family	-0.5	3.09E-02
Clo1313_0030	Formate-tetrahydrofolate ligase (EC 6.3.4.3)	-0.4	1.30E-03
Clo1313_0031	sporulation peptidase YabG	-0.5	1.72E-01
Clo1313_0032	protein of unknown function DUF1021	-0.6	4.95E-04
Clo1313_0033	Peptidoglycan-binding lysin domain	0.1	8.04E-01
Clo1313_0034	4-diphosphocytidyl-2-C-methyl-D-erythritol kinase (EC 2.7.1.148)	-0.2	6.34E-02
Clo1313_0035	transcriptional regulator, GntR family	-0.4	6.65E-04
Clo1313_0036	Heavy metal transport/detoxification protein	0.0	9.78E-01
Clo1313_0037	hypothetical protein	-0.7	5.38E-09
Clo1313_0038	AAA ATPase central domain protein	-0.4	3.89E-05
Clo1313_0039	phage shock protein A (PspA) family protein	-0.5	1.71E-13
Clo1313_0040	hypothetical protein	-0.4	3.10E-04
Clo1313_0041	protein of unknown function DUF342	-0.5	2.92E-06
Clo1313_0042	metallophosphoesterase	-0.2	2.48E-01
Clo1313_0043	SMC domain protein	-0.2	3.42E-01
Clo1313_0044	NLP/P60 protein	-0.3	6.27E-02
Clo1313_0045	Monogalactosyldiacylglycerol synthase (EC 2.4.1.46) -Pseudo gene	-0.3	3.06E-01
Clo1313_0046	spore cortex-lytic enzyme	-0.1	8.06E-01
Clo1313_0047	germination protein YpeB	-0.4	2.70E-01
Clo1313_0048	Abortive infection protein	0.0	8.54E-01
Clo1313_0049	AAA ATPase	-0.1	3.39E-01
Clo1313_0050	primosome, DnaD subunit	-0.4	2.58E-06
Clo1313_0051	metal dependent phosphohydrolase	0.0	7.97E-01
Clo1313_0052	hypothetical protein	0.3	1.61E-02
Clo1313_0053	hypothetical protein	0.3	1.06E-03
Clo1313_0054	type 3a cellulose-binding domain protein	0.1	6.57E-01
Clo1313_0055	copper amine oxidase-like domain-containing protein	-0.1	3.57E-01
Clo1313_0056	MotA/TolQ/ExbB proton channel	0.3	4.11E-02
Clo1313_0057	OmpA/MotB domain protein	1.0	6.82E-13
Clo1313_0058	protein of unknown function DUF1385	-0.5	2.36E-12
Clo1313_0059	protein-(glutamine-N5) methyltransferase, release factor-specific	-0.7	1.47E-10
Clo1313_0060	sporulation integral membrane protein YtvI	-0.3	2.14E-02
Clo1313_0061	electron transport complex, RnfABCDGE type, C subunit	-0.3	1.76E-03
Clo1313_0062	electron transport complex, RnfABCDGE type, D subunit	-0.5	3.08E-06
Clo1313_0063	electron transport complex, RnfABCDGE type, G subunit	-0.4	2.13E-03
Clo1313_0064	electron transport complex, RnfABCDGE type, E subunit	-0.2	2.66E-01
Clo1313_0065	electron transport complex, RnfABCDGE type, A subunit	-0.1	7.17E-01
Clo1313_0066	electron transport complex, RnfABCDGE type, B subunit	0.2	1.76E-01
Clo1313_0067	hypothetical protein	-0.3	3.65E-01
Clo1313_0068	shikimate kinase (EC 2.7.1.71)	-0.6	4.09E-15
Clo1313_0069	Pseudo gene	0.0	9.64E-01

Genes significantly up-regulated at least two-fold

Genes significantly down-regulated at least two-fold

Clo1313_0070	GCN5-related N-acetyltransferase	-0.4	3.70E-04
Clo1313_0071	hypothetical protein	-0.1	6.51E-01
Clo1313_0072	transcriptional regulator, DeoR family	0.8	2.32E-11
Clo1313_0073	glycerol kinase (EC 2.7.1.30)	2.5	3.40E-127
Clo1313_0074	transketolase subunit A (EC 2.2.1.1)	2.6	2.23E-71
Clo1313_0075	transketolase subunit B (EC 2.2.1.1)	2.5	3.63E-31
Clo1313_0076	Alcohol dehydrogenase GroES domain protein	2.2	4.30E-34
Clo1313_0077	hypothetical protein	2.1	2.87E-83
Clo1313_0078	ABC transporter related protein	1.8	1.21E-67
Clo1313_0079	inner-membrane translocator	1.6	2.52E-20
Clo1313_0080	Phosphoglycerate mutase	1.6	3.28E-17
Clo1313_0081	SNF2 helicase associated domain protein	-0.8	1.01E-22
Clo1313_0082	oligopeptidase F. Metallo peptidase. MEROPS family M03B	-0.4	1.08E-03
Clo1313_0083	MazG nucleotide pyrophosphohydrolase	-0.1	8.06E-01
Clo1313_0084	S-layer domain-containing protein	0.7	8.31E-07
Clo1313_0085	tRNA-adenosine deaminase (EC 3.5.4.-)	-0.5	8.51E-10
Clo1313_0086	Pseudo gene	0.3	4.45E-01
Clo1313_0087	Cupin 2 conserved barrel domain protein	0.2	6.36E-01
Clo1313_0088	3D domain-containing protein	-0.1	6.86E-01
Clo1313_0089	transcriptional regulator, LacI family	-0.6	5.38E-07
Clo1313_0090	S-layer domain-containing protein	0.3	2.70E-02
Clo1313_0091	peptidase U57 YabG	0.1	8.86E-01
Clo1313_0092	DNA topoisomerase type IA central domain protein	-0.6	1.38E-05
Clo1313_0093	protein of unknown function DUF368	-0.2	3.77E-02
Clo1313_0094	MscS Mechanosensitive ion channel	-0.1	6.74E-01
Clo1313_0095	protein of unknown function DUF951	-0.2	3.56E-01
Clo1313_0096	peptidase S1 and S6 chymotrypsin/Hap	-0.2	1.33E-01
Clo1313_0097	hypothetical protein	-0.7	1.78E-03
Clo1313_0098	CMP/dCMP deaminase zinc-binding protein	0.0	8.49E-01
Clo1313_0099	acetolactate synthase, large subunit (EC 2.2.1.6)	-0.5	6.67E-06
Clo1313_0100	acetolactate synthase, small subunit (EC 2.2.1.6)	-0.6	5.23E-07
Clo1313_0101	ketol-acid reductoisomerase (EC 1.1.1.86)	-0.8	1.47E-19
Clo1313_0102	2-isopropylmalate synthase/homocitrate synthase family protein	-0.8	5.64E-23
Clo1313_0103	hypothetical protein	-1.0	1.80E-14
Clo1313_0104	RNA polymerase, sigma 28 subunit, SigI	-0.6	1.06E-09
Clo1313_0105	hypothetical protein	-0.6	2.00E-17
Clo1313_0106	hypothetical protein	0.4	2.02E-01
Clo1313_0107	transcriptional regulator, BadM/Rrf2 family	-0.1	6.19E-01
Clo1313_0108	glutamyl-tRNA reductase (EC 1.2.1.70)	0.1	8.04E-01
Clo1313_0109	siroheme synthase	0.3	4.68E-01
Clo1313_0110	hydroxymethylbilane synthase (EC 2.5.1.61)	0.3	4.03E-01
Clo1313_0111	uroporphyrinogen-III synthase (EC 4.2.1.75)/uroporphyrinogen-III C-methyltransferase (EC 2.1.1.107)	0.4	3.68E-01
Clo1313_0112	Porphobilinogen synthase	0.3	5.55E-01
Clo1313_0113	glutamate-1-semialdehyde 2,1-aminomutase (EC 5.4.3.8)	0.4	3.98E-01
Clo1313_0114	sulfate ABC transporter, periplasmic sulfate-binding protein	-0.1	8.75E-01
Clo1313_0115	sulfate ABC transporter, inner membrane subunit CysT	0.0	9.63E-01
Clo1313_0116	sulfate ABC transporter, inner membrane subunit CysW	-0.1	7.54E-01
Clo1313_0117	sulfate ABC transporter, ATPase subunit	-0.2	6.48E-01
Clo1313_0118	phosphoadenylylsulfate reductase (thioredoxin) (EC 1.8.4.8)	-0.2	5.70E-01
Clo1313_0119	sulfate adenylyltransferase subunit 2 (EC 2.7.7.4)	0.1	8.36E-01
Clo1313_0120	adenylylsulfate kinase (EC 2.7.1.25)/sulfate adenylyltransferase subunit 1 (EC 2.7.7.4)	-0.1	7.64E-01
Clo1313_0121	thiamine biosynthesis protein ThiS	-0.1	8.73E-01
Clo1313_0122	UBA/THIF-type NAD/FAD binding protein	0.0	9.80E-01
Clo1313_0123	Mov34/MPN/PAD-1 family protein	-0.1	8.58E-01
Clo1313_0124	nitrite and sulphite reductase 4Fe-4S region	-0.1	7.71E-01
Clo1313_0125	SirA-like domain-containing protein	-0.3	4.79E-01
Clo1313_0126	Lipoprotein LpqB, GerMN domain	-0.2	2.64E-01
Clo1313_0127	RNA polymerase, sigma-24 subunit, ECF subfamily	-0.4	1.86E-06
Clo1313_0128	hypothetical protein	0.0	9.42E-01
Clo1313_0129	hypothetical protein	0.0	8.33E-01
Clo1313_0130	Pseudo gene	0.0	8.91E-01
Clo1313_0131	hypothetical protein	-0.4	9.25E-02
Clo1313_0132	hypothetical protein	-0.5	1.01E-04
Clo1313_0133	alpha-L-arabinofuranosidase domain protein	-0.6	1.04E-06
Clo1313_0134	hypothetical protein	-0.1	8.22E-01
Clo1313_0135	Dockerin type 1	0.1	8.31E-01
Clo1313_0136	glycosyltransferase sugar-binding region containing DXD motif	-0.1	8.38E-01
Clo1313_0137	Radical SAM domain protein	-0.5	1.09E-01
Clo1313_0138	Radical SAM domain protein	-0.2	5.59E-01
Clo1313_0139	Radical SAM domain protein	0.1	6.26E-01
Clo1313_0140	glycosyl transferase group 1	0.2	3.52E-01
Clo1313_0141	methyltransferase FkbM family	0.0	9.78E-01
Clo1313_0142	Radical SAM domain protein	-0.3	3.92E-02
Clo1313_0143	glycosyl transferase family 2	-0.3	7.00E-02
Clo1313_0144	NAD-dependent epimerase/dehydratase	1.0	1.38E-05
Clo1313_0145	DegT/DnrJ/EryC1/StrS aminotransferase	-0.2	1.75E-01

Clo1313_0146	dTDP-4-dehydrorhamnose 3,5-epimerase (EC 5.1.3.13)	0.1	8.22E-01
Clo1313_0147	CDP-glucose 4,6-dehydratase	0.3	3.21E-02
Clo1313_0148	glucose-1-phosphate cytidylyltransferase	0.5	1.17E-03
Clo1313_0149	hypothetical protein	-0.2	9.03E-02
Clo1313_0150	transposase mutator type	-1.0	7.90E-06
Clo1313_0151	hypothetical protein	-0.2	6.01E-01
Clo1313_0152	Radical SAM domain protein	-0.6	1.77E-02
Clo1313_0153	Cupin 2 conserved barrel domain protein	NA	NA
Clo1313_0154	hypothetical protein	0.3	4.68E-01
Clo1313_0155	hypothetical protein	0.2	5.40E-01
Clo1313_0156	hypothetical protein	0.1	5.40E-01
Clo1313_0157	hypothetical protein	-0.5	2.80E-03
Clo1313_0158	Pseudo gene	0.2	6.63E-01
Clo1313_0159	hypothetical protein	-0.4	6.34E-02
Clo1313_0160	ABC transporter related protein	-0.2	7.14E-01
Clo1313_0161	binding-protein-dependent transport systems inner membrane component	0.1	7.57E-01
Clo1313_0162	hypothetical protein	-0.5	2.10E-01
Clo1313_0163	hypothetical protein	-0.4	3.14E-06
Clo1313_0164	adenylate cyclase	0.0	9.54E-01
Clo1313_0165	Ppx/GppA phosphatase	-0.4	8.30E-04
Clo1313_0166	iron-containing alcohol dehydrogenase	0.1	6.30E-01
Clo1313_0167	phosphodiesterase, MJ0936 family	-0.3	8.88E-02
Clo1313_0168	Dihydropteroate synthase (EC 2.5.1.15)	-0.1	6.29E-01
Clo1313_0169	dihydroneopterin aldolase	-0.3	1.24E-01
Clo1313_0170	2-amino-4-hydroxy-6- hydroxymethyldihydropteridine pyrophosphokinase	0.1	7.43E-01
Clo1313_0171	hypothetical protein	-0.2	8.37E-02
Clo1313_0172	biotin/acetyl-CoA-carboxylase ligase	-0.3	1.35E-03
Clo1313_0173	amidohydrolase 2	0.0	7.97E-01
Clo1313_0174	hypothetical protein	0.0	8.63E-01
Clo1313_0175	pantothenate kinase (EC 2.7.1.33)	0.3	3.14E-03
Clo1313_0176	hypothetical protein	0.2	1.58E-01
Clo1313_0177	glycoside hydrolase family 10	-0.2	5.22E-01
Clo1313_0178	hypothetical protein	0.4	2.63E-05
Clo1313_0179	hypothetical protein	-0.4	6.34E-02
Clo1313_0180	bacterial peptide chain release factor 1 (bRF-1)	-0.3	1.33E-03
Clo1313_0181	zinc/iron permease	-0.2	2.37E-01
Clo1313_0182	translation factor SUA5	-0.2	4.52E-02
Clo1313_0183	protein tyrosine phosphatase	-0.2	6.55E-02
Clo1313_0184	ribose-5-phosphate isomerase (EC 5.3.1.6)	-0.4	1.48E-03
Clo1313_0185	uracil phosphoribosyltransferase (EC 2.4.2.9)	-0.6	3.53E-13
Clo1313_0186	CMP/dCMP deaminase zinc-binding protein	-0.2	2.11E-01
Clo1313_0187	Glycosyl transferase, family 4, conserved region	-0.2	6.08E-02
Clo1313_0188	UDP-N-acetylglucosamine 2-epimerase	-0.5	1.14E-08
Clo1313_0189	ATP synthase F0 subcomplex A subunit	0.6	3.45E-07
Clo1313_0190	ATP synthase F0 subcomplex C subunit	0.4	9.43E-03
Clo1313_0191	ATP synthase F0 subcomplex B subunit	0.2	2.01E-01
Clo1313_0192	ATP synthase F1 subcomplex delta subunit	0.2	1.97E-01
Clo1313_0193	ATP synthase F1 subcomplex alpha subunit	0.4	1.08E-04
Clo1313_0194	ATP synthase F1, gamma subunit	0.5	7.04E-05
Clo1313_0195	ATP synthase F1 subcomplex beta subunit	0.3	2.15E-03
Clo1313_0196	ATP synthase F1 subcomplex epsilon subunit	0.5	3.40E-04
Clo1313_0197	hypothetical protein	-0.8	1.35E-18
Clo1313_0198	S-layer domain-containing protein	0.1	4.74E-01
Clo1313_0199	fibronectin type III domain protein	0.3	1.01E-02
Clo1313_0200	S-layer domain-containing protein	-0.5	1.74E-05
Clo1313_0201	protein of unknown function DUF1779	-0.4	1.45E-06
Clo1313_0202	UDP-N-acetylglucosamine 1-carboxyvinyltransferase (EC 2.5.1.7)	-0.4	2.74E-05
Clo1313_0203	stage II sporulation protein D	-0.5	6.54E-02
Clo1313_0204	Peptidase M23	0.2	1.79E-01
Clo1313_0205	sporulation transcriptional regulator SpoIIID	-0.1	7.17E-01
Clo1313_0206	rod shape-determining protein MreB	-0.1	1.64E-01
Clo1313_0207	flagellar hook-basal body protein	0.9	3.53E-13
Clo1313_0208	flagellar hook-basal body protein	0.8	2.63E-09
Clo1313_0209	Flagellar protein FlgJ-like protein	0.1	6.95E-01
Clo1313_0210	exopolysaccharide biosynthesis protein	0.1	6.89E-01
Clo1313_0211	ABC transporter ATP-binding protein	0.2	5.93E-01
Clo1313_0212	Pseudo gene	0.1	5.08E-01
Clo1313_0213	3-hydroxyacyl-[acyl-carrier-protein] dehydratase (EC 4.2.1.-)	-0.4	9.75E-02
Clo1313_0214	UDP-N-acetylmuramate--L-alanine ligase (EC 6.3.2.8)	0.2	2.12E-01
Clo1313_0215	purine operon repressor, PurR	-0.5	4.02E-05
Clo1313_0216	SpoVG family protein	-0.2	6.05E-02
Clo1313_0217	UDP-N-acetylglucosamine pyrophosphorylase (EC 2.7.7.23)/glucosamine-1-phosphate N-acetyltransferase (EC 2.3.1.157)	-0.2	2.87E-01
Clo1313_0218	ribose-phosphate pyrophosphokinase	-0.2	6.85E-02
Clo1313_0219	peptidyl-tRNA hydrolase (EC 3.1.1.29)	-0.2	3.41E-01

Clo1313_0220	transcription-repair coupling factor	-0.3	1.12E-02
Clo1313_0221	PpiC-type peptidyl-prolyl cis-trans isomerase	0.4	4.10E-06
Clo1313_0222	transcriptional regulator, AraC family	-0.1	8.79E-01
Clo1313_0223	transcriptional regulator, AraC family	0.2	2.15E-01
Clo1313_0224	pyridoxamine 5'-phosphate oxidase-related FMN-binding protein	0.0	9.31E-01
Clo1313_0225	integral membrane protein MviN	0.2	6.79E-01
Clo1313_0226	glycosyl transferase group 1	-0.1	8.22E-01
Clo1313_0227	O-antigen polymerase	0.3	3.92E-01
Clo1313_0228	acylneuraminate cytidylyltransferase	0.2	7.27E-01
Clo1313_0229	UDP-N-acetyl-D-glucosamine 2-epimerase, UDP-hydrolysing	0.0	9.95E-01
Clo1313_0230	N-acetylneuraminate synthase (EC 2.5.1.56)	-0.4	2.40E-01
Clo1313_0231	sugar O-acyltransferase, sialic acid O-acetyltransferase NeuD family	0.0	9.30E-01
Clo1313_0232	Nucleotidyl transferase	0.2	5.30E-01
Clo1313_0233	DegT/DnrJ/EryC1/StrS aminotransferase	0.3	4.76E-01
Clo1313_0234	NAD-dependent epimerase/dehydratase	0.2	6.00E-01
Clo1313_0235	Capsule polysaccharide biosynthesis protein	0.2	7.04E-01
Clo1313_0236	ATP-dependent carboxylate-amine ligase domain protein ATP-grasp	-0.1	8.37E-01
Clo1313_0237	sugar transferase	0.1	7.83E-01
Clo1313_0238	HpcH/HpaI aldolase	-0.1	9.12E-01
Clo1313_0239	polysaccharide biosynthesis protein CapD	-0.2	7.53E-01
Clo1313_0240	hypothetical protein	0.2	4.89E-01
Clo1313_0241	lipopolysaccharide biosynthesis protein	-0.3	4.30E-01
Clo1313_0242	capsular exopolysaccharide family	-0.6	2.21E-02
Clo1313_0243	Protein-tyrosine-phosphatase	-0.3	5.16E-01
Clo1313_0244	stage V sporulation protein T	0.0	9.66E-01
Clo1313_0245	MazG family protein	-0.3	9.24E-03
Clo1313_0246	histone family protein DNA-binding protein	-0.2	1.58E-01
Clo1313_0247	RNA-binding S4 domain protein	0.1	7.33E-01
Clo1313_0248	sporulation protein YabP	0.2	5.69E-01
Clo1313_0249	spore cortex biosynthesis protein YabQ	-0.8	2.28E-02
Clo1313_0250	Septum formation initiator	-0.3	9.54E-02
Clo1313_0251	RNA binding S1 domain protein	-0.4	3.85E-03
Clo1313_0252	methyl-accepting chemotaxis sensory transducer	0.0	8.75E-01
Clo1313_0253	2-octaprenylphenol hydroxylase (EC 1.14.13.-)	-0.4	8.32E-04
Clo1313_0254	Zn-finger containing protein	0.3	3.85E-01
Clo1313_0255	ATPase, P-type (transporting), HAD superfamily, subfamily IC	0.2	2.78E-01
Clo1313_0256	transcriptional regulator, TetR family	0.2	5.85E-01
Clo1313_0257	Pseudo gene	-0.3	2.10E-02
Clo1313_0258	hypothetical protein	0.0	9.54E-01
Clo1313_0259	hypothetical protein	-0.3	4.63E-01
Clo1313_0260	hydrolase	0.4	3.01E-01
Clo1313_0261	hypothetical protein	0.2	3.06E-01
Clo1313_0262	Ribosomal protein L7/L12	0.3	3.42E-01
Clo1313_0263	Pseudo gene	0.3	5.61E-01
Clo1313_0264	hypothetical protein	0.1	6.87E-01
Clo1313_0265	hypothetical protein	0.2	3.23E-01
Clo1313_0266	GumN family protein	-0.9	5.59E-15
Clo1313_0267	Pseudo gene	-0.3	3.39E-01
Clo1313_0268	hypothetical protein	0.0	9.39E-01
Clo1313_0269	hypothetical protein	0.0	8.89E-01
Clo1313_0270	peptidase S41	-0.3	8.63E-02
Clo1313_0271	stage II sporulation protein E, protein serine/threonine phosphatase	-0.4	2.64E-03
Clo1313_0272	hypothetical protein	0.2	3.10E-01
Clo1313_0273	hypothetical protein	-0.1	6.84E-01
Clo1313_0274	type IV pilus assembly PilZ	-0.2	4.40E-02
Clo1313_0275	tRNA-U20-dihydrouridine synthase	0.3	7.38E-03
Clo1313_0276	type IV pilus assembly protein PilM	0.2	3.50E-02
Clo1313_0277	thioesterase superfamily protein	0.0	8.12E-01
Clo1313_0278	stage V sporulation protein B	0.6	1.80E-02
Clo1313_0279	conserved hypothetical protein	0.8	2.30E-04
Clo1313_0280	transcriptional regulator, GntR family	-0.2	1.19E-01
Clo1313_0281	UbiA prenyltransferase	0.4	2.95E-02
Clo1313_0282	galactoside O-acetyltransferase	0.2	3.21E-01
Clo1313_0283	glycosyl transferase group 1	0.2	1.28E-01
Clo1313_0284	hypothetical protein	-0.3	5.74E-03
Clo1313_0285	O-antigen polymerase	-0.3	1.04E-02
Clo1313_0286	hypothetical protein	-0.8	1.71E-16
Clo1313_0287	carbohydrate kinase, YjeF related protein	-0.4	4.01E-03
Clo1313_0288	alanine racemase (EC 5.1.1.1)	-0.3	7.92E-05
Clo1313_0289	putative transcriptional regulator, CopG family	0.0	9.94E-01
Clo1313_0290	transcriptional modulator of MazE/toxin, MazF	0.0	9.65E-01
Clo1313_0291	hypothetical protein	0.4	2.38E-04
Clo1313_0292	N-acetylmannosaminyltransferase (EC 2.4.1.187)	-0.1	7.18E-01
Clo1313_0293	polysaccharide pyruvyl transferase CsaB	-0.1	5.69E-01
Clo1313_0294	Protein of unknown function DUF2179	-0.6	7.52E-10

Clo1313_0295	transketolase subunit A (EC 2.2.1.1)	-0.1	2.48E-01
Clo1313_0296	transketolase subunit B (EC 2.2.1.1)	-0.1	4.75E-01
Clo1313_0297	ABC transporter related protein	-0.5	5.81E-09
Clo1313_0298	ABC-2 type transporter	-0.4	5.19E-03
Clo1313_0299	ABC-type uncharacterized transport system	-0.8	1.46E-24
Clo1313_0300	hypothetical protein	-0.7	1.53E-14
Clo1313_0301	hypothetical protein	-0.2	5.38E-01
Clo1313_0302	hypothetical protein	-0.6	5.35E-10
Clo1313_0303	Colicin V production protein	-0.3	7.31E-02
Clo1313_0304	dihydroxy-acid dehydratase	-0.2	2.60E-01
Clo1313_0305	acetolactate synthase, large subunit (EC 2.2.1.6)	-0.5	1.21E-07
Clo1313_0306	LSU ribosomal protein L33P	0.1	7.22E-01
Clo1313_0307	preprotein translocase, SecE subunit	0.1	7.04E-01
Clo1313_0308	transcription antitermination protein nusG	-0.4	3.00E-04
Clo1313_0309	LSU ribosomal protein L11P	-0.6	4.34E-16
Clo1313_0310	LSU ribosomal protein L1P	-0.4	1.21E-04
Clo1313_0311	LSU ribosomal protein L10P	-0.8	1.36E-28
Clo1313_0312	LSU ribosomal protein L12P	-0.8	1.16E-19
Clo1313_0313	DNA-directed RNA polymerase subunit beta (EC 2.7.7.6)	-0.4	6.51E-07
Clo1313_0314	DNA-directed RNA polymerase subunit beta' (EC 2.7.7.6)	-0.4	2.02E-05
Clo1313_0315	LSU ribosomal protein L7AE	-0.4	6.53E-02
Clo1313_0316	SSU ribosomal protein S12P	-0.7	1.67E-12
Clo1313_0317	SSU ribosomal protein S7P	-0.8	1.35E-13
Clo1313_0318	translation elongation factor G	-0.4	5.05E-09
Clo1313_0319	translation elongation factor Tu	-0.3	3.93E-05
Clo1313_0320	RNA polymerase, sigma-24 subunit, ECF subfamily	-0.8	1.82E-10
Clo1313_0321	hypothetical protein	-0.5	1.30E-03
Clo1313_0322	hypothetical protein	-0.7	5.13E-02
Clo1313_0323	VanW family protein	-0.6	3.43E-03
Clo1313_0324	Phosphotransferase system, phosphocarrier protein HPr	0.2	3.97E-01
Clo1313_0325	phosphoenolpyruvate--protein phosphotransferase (EC 2.7.3.9)	-1.0	1.66E-28
Clo1313_0326	Excinuclease ABC subunit C	-0.4	2.88E-03
Clo1313_0327	metallophosphoesterase	-0.1	6.33E-01
Clo1313_0328	trigger factor	0.0	8.85E-01
Clo1313_0329	ATP-dependent Clp protease, proteolytic subunit ClpP	-0.3	9.17E-04
Clo1313_0330	ATP-dependent Clp protease, ATP-binding subunit ClpX	-0.2	5.79E-03
Clo1313_0331	Sigma 54 interacting domain protein	0.2	5.42E-01
Clo1313_0332	O-sialoglycoprotein endopeptidase (EC 3.4.24.57)	-0.3	4.90E-02
Clo1313_0333	Lytic transglycosylase catalytic	0.6	3.38E-04
Clo1313_0334	hypothetical protein	0.3	4.89E-01
Clo1313_0335	protein of unknown function DUF402	-0.6	1.29E-04
Clo1313_0336	PHP domain protein	-0.6	5.40E-09
Clo1313_0337	SsrA-binding protein	-0.3	8.38E-03
Clo1313_0337	SsrA-binding protein	-0.3	3.48E-01
Clo1313_0338	hypothetical protein	0.8	3.71E-03
Clo1313_0339	hypothetical protein	0.4	1.95E-01
Clo1313_0340	hypothetical protein	0.5	6.89E-02
Clo1313_0341	hypothetical protein	0.1	4.97E-01
Clo1313_0342	hypothetical protein	0.3	3.56E-01
Clo1313_0343	beta-lactamase domain-containing protein	-0.3	4.97E-02
Clo1313_0344	ADP-ribosylation/Crystallin J1	-0.5	3.80E-04
Clo1313_0345	Pseudo gene	0.0	9.27E-01
Clo1313_0346	potassium/proton antiporter, CPA1 family (TC 2.A.36)	0.0	9.60E-01
Clo1313_0347	DNA polymerase beta domain protein region	0.1	7.01E-01
Clo1313_0348	transcriptional regulator, AraC family	-0.5	9.88E-04
Clo1313_0349	endoglucanase Cel9V	-0.3	1.24E-02
Clo1313_0350	glycoside hydrolase family 9	-0.2	8.59E-02
Clo1313_0351	transcriptional regulator-like protein	0.2	1.52E-01
Clo1313_0352	hypothetical protein	0.0	8.93E-01
Clo1313_0353	TROVE domain-containing protein	-0.2	5.20E-01
Clo1313_0354	hypothetical protein	-0.3	1.18E-01
Clo1313_0355	hypothetical protein	0.1	8.92E-01
Clo1313_0356	Methyltransferase type 12	-0.3	1.86E-01
Clo1313_0357	polynucleotide 3'-phosphatase/polynucleotide 5'-hydroxyl-kinase/polynucleotide 2',3'-cyclic phosphate phosphodiesterase	-0.4	6.43E-03
Clo1313_0358	iron-sulfur cluster repair di-iron protein	-0.3	3.39E-01
Clo1313_0359	flavodoxin/nitric oxide synthase	-0.3	3.16E-01
Clo1313_0360	transcriptional regulator, Crp/Fnr family	-0.4	4.39E-02
Clo1313_0361	hypothetical protein	-0.6	1.59E-06
Clo1313_0362	hypothetical protein	-0.1	8.44E-01
Clo1313_0363	hypothetical protein	0.2	6.24E-01
Clo1313_0364	nicotinamide mononucleotide transporter PnuC	0.1	5.34E-01
Clo1313_0365	cytidyltransferase-related domain protein	0.2	2.62E-01
Clo1313_0366	hypothetical protein	-0.3	9.25E-02
Clo1313_0367	hypothetical protein	0.0	9.78E-01
Clo1313_0368	Domain of unknown function DUF1801	0.3	1.97E-01
Clo1313_0369	hypothetical protein	0.1	6.63E-01
Clo1313_0370	N-acetylmuramoyl-L-alanine amidase family 2	0.4	2.27E-01

Clo1313_0371	Protein of unknown function DUF2292	-0.2	6.29E-01
Clo1313_0372	cobalamin B12-binding domain protein	0.3	4.72E-01
Clo1313_0373	methyltransferase MtaA/CmuA family	0.4	2.20E-01
Clo1313_0374	methyltransferase MtaA/CmuA family	0.0	9.66E-01
Clo1313_0375	ferredoxin	-0.2	5.73E-01
Clo1313_0376	protein of unknown function DUF1847	0.0	9.66E-01
Clo1313_0377	extracellular solute-binding protein family 3	0.3	3.91E-01
Clo1313_0378	ABC transporter related protein	0.3	4.64E-01
Clo1313_0379	binding-protein-dependent transport systems inner membrane component	0.2	6.17E-01
Clo1313_0380	Phenylacetate--CoA ligase	0.1	7.51E-01
Clo1313_0381	aminotransferase class I and II	-0.5	5.29E-04
Clo1313_0382	pyruvate ferredoxin oxidoreductase, gamma subunit (EC 1.2.7.1)	0.0	9.66E-01
Clo1313_0383	pyruvate ferredoxin/flavodoxin oxidoreductase, delta subunit	0.1	8.90E-01
Clo1313_0384	pyruvate flavodoxin/ferredoxin oxidoreductase domain protein	0.3	5.21E-01
Clo1313_0385	pyruvate ferredoxin oxidoreductase, beta subunit (EC 1.2.7.1)	-0.2	5.96E-01
Clo1313_0386	hypothetical protein	-0.1	8.32E-01
Clo1313_0387	endoribonuclease L-PSP	0.1	8.83E-01
Clo1313_0388	Cys/Met metabolism pyridoxal-phosphate-dependent protein	0.2	6.02E-01
Clo1313_0389	aminotransferase class I and II	0.2	4.85E-01
Clo1313_0390	carbon-monoxide dehydrogenase, catalytic subunit	0.3	4.11E-01
Clo1313_0391	extracellular solute-binding protein family 3	0.2	6.05E-01
Clo1313_0392	binding-protein-dependent transport systems inner membrane component	0.1	7.83E-01
Clo1313_0393	ABC transporter related protein	0.1	8.32E-01
Clo1313_0394	hypothetical protein	0.3	7.02E-02
Clo1313_0395	glycoside hydrolase family 5	0.9	1.32E-08
Clo1313_0396	transcriptional regulator, LacI family	0.5	3.40E-04
Clo1313_0397	Glucan endo-1,3-beta-D-glucosidase	0.7	3.24E-17
Clo1313_0398	Na/Pi-cotransporter II-related protein	-0.2	2.64E-01
Clo1313_0399	Dockerin type 1	1.4	3.77E-27
Clo1313_0400	glycoside hydrolase family 9	0.1	9.07E-02
Clo1313_0401	two component transcriptional regulator, winged helix family	-0.1	7.09E-01
Clo1313_0402	integral membrane sensor signal transduction histidine kinase	0.0	9.95E-01
Clo1313_0403	lysyl-tRNA synthetase, class I (EC 6.1.1.6)	-0.3	1.91E-05
Clo1313_0404	Pseudo gene	0.1	7.86E-01
Clo1313_0405	CheW protein	0.7	9.13E-03
Clo1313_0406	CheA signal transduction histidine kinase	0.6	1.57E-05
Clo1313_0407	methyl-accepting chemotaxis sensory transducer	0.6	9.07E-09
Clo1313_0408	MCP methyltransferase, CheR-type	0.5	1.18E-01
Clo1313_0409	response regulator receiver modulated CheB methylesterase	0.2	6.33E-01
Clo1313_0410	oxidoreductase domain protein	-0.1	4.10E-01
Clo1313_0411	protein of unknown function DUF21	-0.5	3.89E-07
Clo1313_0412	TipAS antibiotic-recognition domain-containing protein	-0.5	1.70E-02
Clo1313_0413	glycoside hydrolase family 5	-0.4	4.92E-05
Clo1313_0414	Protein of unknown function DUF3592	-0.2	2.94E-01
Clo1313_0415	Phosphoenolpyruvate carboxykinase (GTP)	-1.4	1.96E-62
Clo1313_0416	SSU ribosomal protein S30P/sigma 54 modulation protein	-0.4	2.37E-06
Clo1313_0417	ATP-dependent DNA helicase PcrA	-0.1	1.44E-01
Clo1313_0418	S-layer domain-containing protein	0.0	8.33E-01
Clo1313_0419	peptidase C39 bacteriocin processing	-0.2	2.14E-01
Clo1313_0420	Dockerin type 1	0.2	6.02E-01
Clo1313_0421	ATP phosphoribosyltransferase regulatory subunit (EC 2.4.2.17)	1.1	7.08E-14
Clo1313_0422	ATP phosphoribosyltransferase catalytic subunit (EC 2.4.2.17)	1.0	1.06E-07
Clo1313_0423	histidinol dehydrogenase	0.9	1.55E-09
Clo1313_0424	histidinol phosphate aminotransferase apoenzyme (EC 2.6.1.9)	0.6	2.39E-06
Clo1313_0425	imidazoleglycerol-phosphate dehydratase (EC 4.2.1.19)	0.7	9.40E-08
Clo1313_0426	phosphoribosylaminoimidazole-succinocarboxamide synthase (EC 6.3.2.6)	0.4	8.17E-04
Clo1313_0427	imidazole glycerol phosphate synthase subunit hisH (EC 2.4.2.-)	0.3	2.39E-02
Clo1313_0428	1-(5-phosphoribosyl)-5-[(5-phosphoribosylamino)methylideneamino] imidazole-4-carboxamide isomerase (EC 5.3.1.16)	0.5	3.82E-04
Clo1313_0429	imidazoleglycerol phosphate synthase, cyclase subunit	0.3	4.26E-02
Clo1313_0430	phosphoribosyl-AMP cyclohydrolase (EC 3.5.4.19)/phosphoribosyl-ATP pyrophosphatase (EC 3.6.1.31)	0.4	6.04E-03
Clo1313_0431	putative transcriptional regulator	0.5	3.74E-04
Clo1313_0432	Chaperonin Cpn10	-0.2	1.09E-01
Clo1313_0433	chaperonin GroEL	-0.2	3.92E-02
Clo1313_0434	type IV pilus assembly PilZ	0.1	6.67E-01
Clo1313_0435	hypothetical protein	0.0	8.34E-01
Clo1313_0436	glycoside hydrolase family 18	-0.3	3.89E-04
Clo1313_0437	hypothetical protein	-0.4	5.99E-03
Clo1313_0438	transcription elongation factor GreA	-0.4	1.30E-03
Clo1313_0439	anti-sigma-factor antagonist	0.3	3.85E-02
Clo1313_0440	putative anti-sigma regulatory factor, serine/threonine protein kinase	0.1	6.92E-01

Clo1313_0441	RNA polymerase, sigma 28 subunit, Sig B/F/G subfamily	-0.1	3.97E-01
Clo1313_0442	ATP-binding region ATPase domain protein	-0.4	1.41E-03
Clo1313_0443	hypothetical protein	0.2	4.11E-01
Clo1313_0444	SSU ribosomal protein S10P	-0.3	3.11E-02
Clo1313_0445	LSU ribosomal protein L3P	-0.4	2.89E-04
Clo1313_0446	LSU ribosomal protein L4P	-0.6	6.36E-08
Clo1313_0447	LSU ribosomal protein L23P	-0.4	2.86E-04
Clo1313_0448	LSU ribosomal protein L2P	-0.4	2.72E-03
Clo1313_0449	ribosomal protein S19	-0.5	3.91E-04
Clo1313_0450	LSU ribosomal protein L22P	-0.5	6.93E-06
Clo1313_0451	ribosomal protein S3	-0.7	6.85E-08
Clo1313_0452	ribosomal protein L16	-0.7	1.32E-06
Clo1313_0453	LSU ribosomal protein L29P	-0.6	1.96E-04
Clo1313_0454	SSU ribosomal protein S17P	-0.6	3.47E-05
Clo1313_0455	LSU ribosomal protein L14P	-0.6	1.51E-05
Clo1313_0456	ribosomal protein L24	-0.6	3.11E-07
Clo1313_0457	LSU ribosomal protein L5P	-0.8	1.32E-08
Clo1313_0458	ribosomal protein S14	-0.3	7.94E-02
Clo1313_0459	ribosomal protein S8	-0.7	7.37E-10
Clo1313_0460	LSU ribosomal protein L6P	-0.6	1.92E-06
Clo1313_0461	LSU ribosomal protein L18P	-0.5	1.80E-04
Clo1313_0462	SSU ribosomal protein S5P	-0.5	4.09E-06
Clo1313_0463	LSU ribosomal protein L30P	-0.4	1.17E-02
Clo1313_0464	LSU ribosomal protein L15P	-0.5	1.42E-03
Clo1313_0465	protein translocase subunit secY/sec61 alpha	-0.6	1.41E-06
Clo1313_0466	Adenylate kinase (EC 2.7.4.3)	-0.6	1.16E-08
Clo1313_0467	methionine aminopeptidase, type I (EC 3.4.11.18)	-0.4	1.08E-03
Clo1313_0468	hypothetical protein	-0.5	1.49E-03
Clo1313_0469	translation initiation factor IF-1	-0.1	6.42E-01
Clo1313_0470	ribosomal protein L36	-0.8	2.00E-05
Clo1313_0471	SSU ribosomal protein S13P	-0.6	1.63E-06
Clo1313_0472	SSU ribosomal protein S11P	-0.7	3.86E-09
Clo1313_0473	SSU ribosomal protein S4P	-0.5	6.25E-07
Clo1313_0474	DNA-directed RNA polymerase subunit alpha (EC 2.7.7.6)	-0.7	5.99E-16
Clo1313_0475	LSU ribosomal protein L17P	-0.6	1.33E-11
Clo1313_0476	Cobalt ATP-binding cassette-like protein	0.0	9.66E-01
Clo1313_0477	methyltransferase small	0.3	1.87E-01
Clo1313_0478	type III restriction protein res subunit	0.6	2.30E-04
Clo1313_0479	protein of unknown function DUF55	0.5	6.56E-02
Clo1313_0480	DNA methylase N-4/N-6 domain protein	0.8	3.54E-05
Clo1313_0481	hypothetical protein	0.2	6.39E-01
Clo1313_0482	regulatory protein DeoR	0.3	1.01E-01
Clo1313_0483	transcriptional regulator, LuxR family	0.1	5.47E-01
Clo1313_0484	hypothetical protein	-0.3	4.64E-01
Clo1313_0485	copper amine oxidase-like domain-containing protein	-0.3	5.81E-02
Clo1313_0486	Resolvase domain protein	-0.1	6.25E-01
Clo1313_0487	ABC transporter related protein	-0.2	2.13E-01
Clo1313_0488	cobalt transport protein	-0.2	5.10E-01
Clo1313_0489	glucokinase, ROK family	-0.3	1.48E-02
Clo1313_0490	tRNA pseudouridine synthase A	0.0	9.78E-01
Clo1313_0491	transcriptional regulator, CarD family	0.0	9.82E-01
Clo1313_0492	2-C-methyl-D-erythritol 4-phosphate cytidylyltransferase	0.1	8.39E-01
Clo1313_0493	ABC transporter related protein	0.0	9.03E-01
Clo1313_0494	ABC-2 type transporter	0.2	3.54E-01
Clo1313_0495	integral membrane sensor signal transduction histidine kinase	0.0	9.40E-01
Clo1313_0496	histidine kinase	-0.1	6.97E-01
Clo1313_0497	2-C-methyl-D-erythritol 2,4-cyclodiphosphate synthase (EC 4.6.1.12)	0.4	1.01E-02
Clo1313_0498	prolyl-tRNA synthetase (EC 6.1.1.15)	0.1	5.82E-01
Clo1313_0499	SCP-like extracellular	0.3	3.04E-01
Clo1313_0500	Pectinesterase	0.4	3.72E-01
Clo1313_0501	Pectate lyase/Amb allergen	0.2	4.55E-01
Clo1313_0502	hypothetical protein	-0.1	7.98E-01
Clo1313_0503	IstB domain protein ATP-binding protein	0.0	9.24E-01
Clo1313_0504	Pseudo gene	-0.3	4.40E-01
Clo1313_0505	hypothetical protein	-0.4	4.12E-03
Clo1313_0506	Pseudo gene	-0.4	4.10E-04
Clo1313_0507	Pseudo gene	NA	NA
Clo1313_0508	Pseudo gene	NA	NA
Clo1313_0509	hypothetical protein	-0.3	2.51E-01
Clo1313_0510	extracellular solute-binding protein family 5	-0.6	1.83E-12
Clo1313_0511	oligopeptide/dipeptide ABC transporter, ATPase subunit	-0.4	2.61E-06
Clo1313_0512	oligopeptide/dipeptide ABC transporter, ATPase subunit	-0.4	1.69E-04
Clo1313_0513	binding-protein-dependent transport systems inner membrane component	-0.6	7.18E-09
Clo1313_0514	binding-protein-dependent transport systems inner membrane component	-0.4	2.90E-04
Clo1313_0515	copper amine oxidase-like domain-containing protein	0.1	9.01E-01

Clo1313_0516	major facilitator superfamily MFS_1	0.1	7.46E-01
Clo1313_0517	polyferredoxin	-0.2	4.30E-01
Clo1313_0518	transcriptional regulator, GntR family	-0.2	6.62E-01
Clo1313_0519	ABC transporter related protein	-0.3	1.58E-01
Clo1313_0520	hypothetical protein	-0.1	7.82E-01
Clo1313_0521	glycoside hydrolase family 11	-0.2	2.66E-01
Clo1313_0522	glycoside hydrolase family 11	-0.3	2.07E-01
Clo1313_0523	sugar fermentation stimulation protein	-0.2	2.50E-02
Clo1313_0524	hypothetical protein	0.0	8.22E-01
Clo1313_0525	RNA polymerase, sigma 28 subunit, SigI	-0.2	6.64E-01
Clo1313_0526	hypothetical protein	-0.5	1.65E-01
Clo1313_0527	transcriptional regulator, Crp/Fnr family	0.1	6.15E-01
Clo1313_0528	4Fe-4S ferredoxin iron-sulfur binding domain-containing protein	0.2	4.01E-01
Clo1313_0529	hypothetical protein	0.0	9.54E-01
Clo1313_0530	hypothetical protein	-0.5	8.70E-02
Clo1313_0531	amino acid ABC transporter substrate-binding protein, PAAT family (TC 3.A.1.3.-)	0.5	2.32E-01
Clo1313_0532	methyl-accepting chemotaxis sensory transducer	0.3	3.82E-01
Clo1313_0533	Pseudo gene	-0.4	6.95E-02
Clo1313_0534	hypothetical protein	-0.3	5.46E-01
Clo1313_0535	hypothetical protein	-0.1	8.85E-01
Clo1313_0536	hypothetical protein	-0.1	7.82E-01
Clo1313_0537	hypothetical protein	-0.1	6.74E-01
Clo1313_0538	hypothetical protein	-0.1	8.57E-01
Clo1313_0539	glycosyltransferase 36	-0.3	1.99E-05
Clo1313_0540	hypothetical protein	0.1	8.44E-01
Clo1313_0541	peptide methionine sulfoxide reductase	0.2	8.07E-02
Clo1313_0542	NADH:flavin oxidoreductase/NADH oxidase	0.4	5.02E-04
Clo1313_0543	RNA polymerase, sigma-24 subunit, ECF subfamily	0.7	8.89E-07
Clo1313_0544	hypothetical protein	1.1	2.97E-22
Clo1313_0545	hypothetical protein	0.4	2.02E-05
Clo1313_0546	hypothetical protein	0.6	2.83E-04
Clo1313_0547	intracellular protease, PfpI family	0.2	3.32E-01
Clo1313_0548	hypothetical protein	0.3	4.07E-01
Clo1313_0549	ABC transporter related protein	0.1	4.88E-01
Clo1313_0550	hypothetical protein	0.0	9.48E-01
Clo1313_0551	phosphate transporter	-0.1	4.56E-01
Clo1313_0552	putative phosphate transport regulator	-0.4	9.87E-08
Clo1313_0553	hypothetical protein	-0.2	1.00E-01
Clo1313_0554	hydrogenase, Fe-only	-0.6	5.99E-07
Clo1313_0555	Peptidoglycan-binding lysin domain	-0.1	7.65E-01
Clo1313_0556	ErfK/YbiS/YcfS/YnhG family protein	0.2	5.49E-01
Clo1313_0557	Peptidoglycan-binding lysin domain	-0.1	8.49E-01
Clo1313_0558	Superoxide dismutase	0.2	5.37E-01
Clo1313_0559	hypothetical protein	0.2	5.03E-01
Clo1313_0560	Pseudo gene	0.2	7.32E-01
Clo1313_0561	Pseudo gene	0.1	7.53E-01
Clo1313_0562	hypothetical protein	0.0	9.79E-01
Clo1313_0563	Carbohydrate binding family 6	0.2	3.85E-01
Clo1313_0564	hydrogenase expression/formation protein HypE	0.1	7.91E-01
Clo1313_0565	hydrogenase expression/formation protein HypD	0.3	1.10E-01
Clo1313_0566	hydrogenase assembly chaperone hypC/hupF	0.4	3.40E-01
Clo1313_0567	(NiFe) hydrogenase maturation protein HypF	0.2	3.12E-01
Clo1313_0568	hydrogenase accessory protein HypB	-0.2	4.87E-01
Clo1313_0569	hydrogenase expression/synthesis HypA	0.1	6.97E-01
Clo1313_0570	4Fe-4S ferredoxin iron-sulfur binding domain-containing protein	0.0	9.66E-01
Clo1313_0571	ech hydrogenase subunit E	-0.3	6.38E-02
Clo1313_0572	ech hydrogenase subunit D	-0.2	1.76E-01
Clo1313_0573	NADH ubiquinone oxidoreductase 20 kDa subunit	-0.5	4.97E-02
Clo1313_0574	respiratory-chain NADH dehydrogenase subunit 1	-0.2	3.56E-01
Clo1313_0575	ech hydrogenase subunit A	-0.2	2.80E-01
Clo1313_0576	hypothetical protein	-0.3	2.24E-01
Clo1313_0577	transcription elongation factor GreA/GreB domain-containing protein	-0.1	7.56E-01
Clo1313_0578	Citrate synthase	-0.5	1.37E-06
Clo1313_0579	Pyridoxal-dependent decarboxylase	0.4	3.98E-02
Clo1313_0580	CheW protein	0.1	7.13E-01
Clo1313_0581	methyl-accepting chemotaxis sensory transducer with Cache sensor	-0.3	3.03E-02
Clo1313_0582	hypothetical protein	-0.1	7.71E-01
Clo1313_0583	hypothetical protein	0.3	5.29E-02
Clo1313_0584	hypothetical protein	0.2	1.14E-01
Clo1313_0585	hypothetical protein	0.4	1.04E-02
Clo1313_0586	D-isomer specific 2-hydroxyacid dehydrogenase NAD-binding protein	-0.8	4.50E-20
Clo1313_0587	methyl-accepting chemotaxis sensory transducer	0.0	8.93E-01
Clo1313_0588	nitroreductase	-0.2	3.43E-01
Clo1313_0589	copper amine oxidase-like domain-containing protein	0.6	3.64E-04

Clo1313_0590	cell division protein FtsK/SpoIIIE	-0.1	4.46E-01
Clo1313_0591	Protein of unknown function DUF2383	-0.6	2.17E-03
Clo1313_0592	UbiA prenyltransferase	-0.1	3.36E-01
Clo1313_0593	hypothetical protein	-0.2	4.16E-02
Clo1313_0594	hypothetical protein	0.5	2.00E-02
Clo1313_0595	protein of unknown function DUF975	0.2	1.29E-01
Clo1313_0596	FHA domain protein	0.1	8.51E-01
Clo1313_0597	cell cycle protein	0.0	9.54E-01
Clo1313_0598	Peptidoglycan glycosyltransferase	0.1	5.60E-01
Clo1313_0599	DNA repair protein RadC	0.5	3.99E-02
Clo1313_0600	Tetratricopeptide TPR_1 repeat-containing protein	0.0	9.54E-01
Clo1313_0601	Fibronectin type III domain protein	0.8	2.64E-04
Clo1313_0602	transposase mutator type	0.1	6.92E-01
Clo1313_0603	YD repeat protein	-0.4	2.30E-07
Clo1313_0604	hypothetical protein	0.0	8.90E-01
Clo1313_0605	hypothetical protein	0.1	7.95E-01
Clo1313_0606	hypothetical protein	-0.1	7.59E-01
Clo1313_0607	Pseudo gene	NA	NA
Clo1313_0608	Pseudo gene	0.3	2.19E-01
Clo1313_0609	hypothetical protein	0.6	5.42E-03
Clo1313_0610	phosphotransferase KptA/Tpt1	-0.1	7.95E-01
Clo1313_0611	hypothetical protein	-0.1	7.91E-01
Clo1313_0612	hypothetical protein	-0.8	3.75E-17
Clo1313_0613	Cephalosporin-C deacetylase	-0.1	6.48E-01
Clo1313_0614	polysaccharide biosynthesis protein	0.1	7.58E-01
Clo1313_0615	hypothetical protein	-0.4	1.04E-04
Clo1313_0616	ABC transporter related protein	-0.5	4.20E-08
Clo1313_0617	membrane spanning protein	-0.3	1.04E-02
Clo1313_0618	integral membrane sensor signal transduction histidine kinase	-0.3	1.17E-02
Clo1313_0619	two component transcriptional regulator, winged helix family	-0.1	7.34E-01
Clo1313_0620	sporulation protein YyaC	0.3	1.47E-01
Clo1313_0621	protein of unknown function DUF1540	-0.1	7.66E-01
Clo1313_0622	acyl-ACP thioesterase	0.2	2.85E-01
Clo1313_0623	HAD-superfamily hydrolase, subfamily IA, variant 3	0.2	4.63E-01
Clo1313_0624	Cof-like hydrolase	-0.2	1.28E-01
Clo1313_0625	Protein of unknown function DUF3520	-0.3	1.31E-02
Clo1313_0626	Radical SAM domain protein	0.0	7.42E-01
Clo1313_0627	cellulosome anchoring protein cohesin region	0.1	3.75E-01
Clo1313_0628	cellulosome anchoring protein cohesin region	0.3	3.15E-02
Clo1313_0629	cellulosome anchoring protein cohesin region	-0.2	2.20E-02
Clo1313_0630	cellulosome anchoring protein cohesin region	0.0	9.58E-01
Clo1313_0631	transglutaminase domain-containing protein	-0.5	1.19E-04
Clo1313_0632	EmrB/QacA family drug resistance transporter	0.4	3.05E-01
Clo1313_0633	hypothetical protein	0.4	3.57E-01
Clo1313_0634	rRNA (guanine-N(2)-)-methyltransferase	0.6	7.90E-05
Clo1313_0635	response regulator receiver protein	0.4	3.69E-02
Clo1313_0636	3-phosphoshikimate 1-carboxyvinyltransferase (EC 2.5.1.19)	-0.4	5.97E-04
Clo1313_0637	sporulation transcriptional activator Spo0A	-0.2	1.63E-01
Clo1313_0638	histone family protein DNA-binding protein	0.6	8.80E-02
Clo1313_0639	UspA domain-containing protein	0.1	7.22E-01
Clo1313_0640	hydro-lyase, Fe-S type, tartrate/fumarate subfamily, alpha subunit	0.1	8.06E-01
Clo1313_0641	hydro-lyase, Fe-S type, tartrate/fumarate subfamily, beta subunit	-0.7	1.44E-20
Clo1313_0642	Domain of unknown function DUF1858	0.1	7.51E-01
Clo1313_0643	Adenylosuccinate synthetase (EC 6.3.4.4)	-0.2	1.95E-02
Clo1313_0644	transposase IS200-family protein	0.0	9.31E-01
Clo1313_0645	glycosyl transferase family 2	-0.1	5.26E-01
Clo1313_0646	glycosyl transferase family 39	0.0	8.65E-01
Clo1313_0647	hypothetical protein	-0.1	3.65E-01
Clo1313_0648	glycosyl transferase family 2	0.2	5.14E-02
Clo1313_0649	hypothetical protein	0.2	6.98E-01
Clo1313_0650	glycosyl transferase family 2	0.1	8.62E-01
Clo1313_0651	diaminopimelate epimerase (EC 5.1.1.7)	-0.8	2.42E-03
Clo1313_0652	LL-diaminopimelate aminotransferase apoenzyme (EC 2.6.1.83)	-0.7	2.47E-02
Clo1313_0653	transcriptional attenuator, LytR family	-0.9	9.93E-05
Clo1313_0654	2-hydroxyglutaryl-CoA dehydratase D-component	-0.7	1.85E-10
Clo1313_0655	CoA-substrate-specific enzyme activase	-0.3	1.06E-03
Clo1313_0656	preQ(0) biosynthesis protein QueC	-0.1	6.63E-01
Clo1313_0657	6-pyruvoyl tetrahydropterin synthase and hypothetical protein	-0.2	2.64E-01
Clo1313_0658	Radical SAM domain protein	-0.1	4.98E-01
Clo1313_0659	phage SPO1 DNA polymerase-related protein	-0.1	6.77E-01
Clo1313_0660	Uracil-DNA glycosylase superfamily	0.0	9.01E-01
Clo1313_0661	UBA/THIF-type NAD/FAD binding protein	0.1	3.56E-01
Clo1313_0662	transposase IS200-family protein	-0.1	6.47E-01
Clo1313_0663	hypothetical protein	0.2	2.30E-01
Clo1313_0664	hypothetical protein	0.0	9.95E-01
Clo1313_0665	glycosidase related protein	0.1	7.10E-01
Clo1313_0666	nucleotidyltransferase	0.2	1.15E-01
Clo1313_0667	glycosyl transferase group 1	-0.2	1.06E-01

Clo1313_0668	hypothetical protein	0.0	9.83E-01
Clo1313_0669	mannose-6-phosphate isomerase, type 1 (EC 5.3.1.8)	0.0	9.66E-01
Clo1313_0670	zinc/iron permease	-0.2	1.12E-01
Clo1313_0671	hemerythrin-like metal-binding protein	0.1	5.73E-01
Clo1313_0672	flavin reductase domain protein FMN-binding protein	-0.2	7.49E-02
Clo1313_0673	pyruvate ferredoxin/flavodoxin oxidoreductase	-0.1	6.79E-01
Clo1313_0674	hypothetical protein	-0.2	3.05E-01
Clo1313_0675	S-layer domain-containing protein	-0.4	1.99E-06
Clo1313_0676	Peptidoglycan-binding domain 1 protein	-0.5	1.21E-11
Clo1313_0677	AMP-dependent synthetase and ligase	-0.1	1.07E-01
Clo1313_0678	heat shock protein Hsp20	0.2	4.64E-01
Clo1313_0679	NUDIX hydrolase	0.0	8.62E-01
Clo1313_0680	peptidase M56 BlaR1	0.1	5.59E-01
Clo1313_0681	hypothetical protein	0.2	2.90E-01
Clo1313_0682	transporter, YbiR family	0.4	9.75E-02
Clo1313_0683	hypothetical protein	0.5	8.43E-03
Clo1313_0684	von Willebrand factor type A	0.4	9.48E-04
Clo1313_0685	Dockerin type 1	0.4	2.53E-02
Clo1313_0686	alpha/beta hydrolase fold protein	0.5	4.79E-02
Clo1313_0687	Pseudo gene	0.6	9.08E-02
Clo1313_0688	hypothetical protein	0.1	8.93E-01
Clo1313_0689	peptidase S8 and S53 subtilisin kexin sedolisin	0.3	1.34E-03
Clo1313_0690	hypothetical protein	-0.1	7.14E-01
Clo1313_0691	hypothetical protein	0.1	8.51E-01
Clo1313_0692	transcriptional regulator, TetR family	0.0	9.91E-01
Clo1313_0693	Carbohydrate binding family 6	0.5	9.45E-02
Clo1313_0694	hypothetical protein	0.1	5.35E-01
Clo1313_0695	Pseudo gene	-0.1	8.58E-01
Clo1313_0696	Penicillinase repressor	-0.2	6.07E-01
Clo1313_0697	peptidase M56 BlaR1	-0.1	7.12E-01
Clo1313_0698	ABC transporter related protein	0.3	4.67E-03
Clo1313_0699	ABC transporter related protein	0.2	4.17E-02
Clo1313_0700	aminoacyl-histidine dipeptidase	0.4	5.38E-05
Clo1313_0701	cobalamin biosynthesis protein CobD	0.1	6.02E-01
Clo1313_0702	adenosylcobinamide kinase (EC 2.7.1.156)/adenosylcobinamide-phosphate guanylyltransferase (EC 2.7.7.62)	-0.2	3.99E-01
Clo1313_0703	cobalamin-5'-phosphate synthase (EC 2.7.8.26)	0.0	9.51E-01
Clo1313_0704	alpha-ribazole phosphatase	-0.2	3.14E-01
Clo1313_0705	hypothetical protein	-0.1	3.82E-01
Clo1313_0706	beta-lactamase superfamily hydrolase	0.2	4.98E-02
Clo1313_0707	methyl-accepting chemotaxis sensory transducer	0.0	9.76E-01
Clo1313_0708	pyruvate carboxyltransferase	-0.3	1.83E-02
Clo1313_0709	aconitase (EC 4.2.1.3)	-0.3	2.33E-02
Clo1313_0710	transcriptional regulator, GntR family	-0.3	1.23E-02
Clo1313_0711	RNA methylase, NOL1/NOP2/sun family	-0.3	1.78E-01
Clo1313_0712	ribosomal small subunit pseudouridine synthase A (EC 5.4.99.-)	-0.2	5.23E-01
Clo1313_0713	protein-L-isoaspartate(D-aspartate) O-methyltransferase	-0.2	2.86E-01
Clo1313_0714	Carbohydrate binding family 25	0.1	5.96E-01
Clo1313_0715	two component transcriptional regulator, AraC family	0.1	3.79E-01
Clo1313_0716	PpiC-type peptidyl-prolyl cis-trans isomerase	-0.2	2.10E-01
Clo1313_0717	glucose-1-phosphate adenylyltransferase	-0.1	2.21E-01
Clo1313_0718	glucose-1-phosphate adenylyltransferase, GlgD subunit	-0.4	7.43E-05
Clo1313_0719	diacylglycerol kinase catalytic region	-0.5	4.49E-06
Clo1313_0720	Enoyl-[acyl-carrier-protein] reductase [NADH] (EC 1.3.1.9)	-0.2	7.03E-02
Clo1313_0721	ABC transporter related protein	0.1	8.93E-01
Clo1313_0722	hypothetical protein	-0.2	6.51E-01
Clo1313_0723	protein of unknown function DUF214	-0.3	2.31E-01
Clo1313_0724	sodium/calcium exchanger membrane region	0.1	7.59E-01
Clo1313_0725	hypothetical protein	0.0	9.95E-01
Clo1313_0726	protein of unknown function DUF421	0.1	8.48E-01
Clo1313_0727	small acid-soluble spore protein alpha/beta type	0.5	3.58E-02
Clo1313_0728	Monogalactosyldiacylglycerol synthase	0.5	9.43E-08
Clo1313_0729	protein of unknown function DUF342	0.2	1.34E-01
Clo1313_0730	Serine-type D-Ala-D-Ala carboxypeptidase	0.0	9.95E-01
Clo1313_0731	hypothetical protein	0.3	6.14E-02
Clo1313_0732	TrkA-N domain protein	0.1	2.89E-01
Clo1313_0733	cation transporter	0.4	4.87E-03
Clo1313_0734	tRNA-guanine transglycosylase, various specificities	0.2	2.19E-01
Clo1313_0735	Radical SAM domain protein	0.0	7.95E-01
Clo1313_0736	putative anti-sigma regulatory factor, serine/threonine protein kinase	0.1	7.27E-01
Clo1313_0737	copper amine oxidase-like domain-containing protein	-0.2	2.03E-02
Clo1313_0738	hypothetical protein	-0.3	6.74E-04
Clo1313_0739	helicase, RecD/TraA family	-0.5	1.74E-05
Clo1313_0740	hypothetical protein	-0.4	6.33E-08
Clo1313_0741	hypothetical protein	0.6	1.05E-02
Clo1313_0742	phospholipase C zinc-binding protein	0.2	6.10E-01
Clo1313_0743	Sporulation lipoprotein YhcN/YlaJ-like protein	0.3	4.14E-01

Clo1313_0744	hypothetical protein	-0.2	6.99E-01
Clo1313_0745	Uncharacterized conserved protein UCP033563	-0.8	1.57E-14
Clo1313_0746	PRC-barrel domain protein	-0.4	3.18E-01
Clo1313_0747	hypothetical protein	-0.3	3.74E-01
Clo1313_0748	protein of unknown function UPF0118	0.1	8.90E-01
Clo1313_0749	alanyl-tRNA synthetase (EC 6.1.1.7)	-0.4	7.00E-04
Clo1313_0750	CRISPR-associated protein TM1802-like protein	0.4	8.89E-02
Clo1313_0751	hypothetical protein	0.1	7.54E-01
Clo1313_0752	hypothetical protein	0.1	7.76E-01
Clo1313_0753	hypothetical protein	-0.1	7.28E-01
Clo1313_0754	hypothetical protein	-0.1	8.62E-01
Clo1313_0755	hypothetical protein	-0.5	1.65E-03
Clo1313_0756	protein of unknown function DUF324	-0.5	1.82E-02
Clo1313_0757	hypothetical protein	-0.3	3.81E-02
Clo1313_0758	protein of unknown function DUF324	0.0	9.95E-01
Clo1313_0759	hypothetical protein	-0.4	1.04E-01
Clo1313_0760	protein of unknown function DUF324	-0.5	6.57E-10
Clo1313_0761	hypothetical protein	-0.8	8.84E-05
Clo1313_0762	CRISPR-associated protein, TIGR02710 family	-0.3	3.60E-02
Clo1313_0763	Pseudo gene	0.2	2.40E-01
Clo1313_0764	CRISPR-associated protein, Cas2 family	-0.2	6.11E-01
Clo1313_0765	CRISPR-associated exonuclease, Cas4 family	0.1	7.00E-01
Clo1313_0766	transposase mutator type	-0.1	5.73E-01
Clo1313_0767	hypothetical protein	0.1	8.49E-01
Clo1313_0768	Pseudo gene	-0.2	5.73E-01
Clo1313_0769	hypothetical protein	-0.2	7.28E-01
Clo1313_0770	hypothetical protein	-0.6	1.33E-02
Clo1313_0771	hypothetical protein	-0.2	5.73E-01
Clo1313_0772	hypothetical protein	-0.2	3.93E-01
Clo1313_0773	Integrase catalytic region	NA	NA
Clo1313_0774	Pseudo gene	-0.2	4.97E-01
Clo1313_0775	Pseudo gene	-0.6	1.67E-03
Clo1313_0776	Pseudo gene	-0.1	6.69E-01
Clo1313_0777	hypothetical protein	-0.5	1.02E-03
Clo1313_0778	copper amine oxidase-like domain-containing protein	0.0	9.54E-01
Clo1313_0779	copper amine oxidase-like domain-containing protein	0.2	1.76E-01
Clo1313_0780	copper amine oxidase-like domain-containing protein	-0.1	6.39E-01
Clo1313_0781	hypothetical protein	-0.1	1.18E-01
Clo1313_0782	hypothetical protein	0.1	7.80E-01
Clo1313_0783	YD repeat protein	0.0	9.54E-01
Clo1313_0784	Ankyrin	0.0	9.24E-01
Clo1313_0785	transposase mutator type	0.0	9.68E-01
Clo1313_0786	YD repeat protein	-0.6	3.88E-04
Clo1313_0787	hypothetical protein	-0.2	6.29E-01
Clo1313_0788	hypothetical protein	0.0	9.54E-01
Clo1313_0789	hypothetical protein	-0.8	5.54E-03
Clo1313_0790	transposase mutator type	-0.1	6.27E-01
Clo1313_0791	FAD dependent oxidoreductase	0.1	7.86E-01
Clo1313_0792	hypothetical protein	0.0	9.62E-01
Clo1313_0793	amino acid ABC transporter substrate-binding protein, PAAT family (TC 3.A.1.3.-)	-0.4	8.84E-09
Clo1313_0794	polar amino acid ABC transporter, inner membrane subunit	-0.1	1.76E-01
Clo1313_0795	amino acid ABC transporter ATP-binding protein, PAAT family (TC 3.A.1.3.-)	-0.4	1.67E-03
Clo1313_0796	hypothetical protein	-0.2	2.04E-01
Clo1313_0797	hypothetical protein	0.2	4.66E-01
Clo1313_0798	Pseudo gene	-0.1	7.38E-01
Clo1313_0799	hypothetical protein	-0.3	1.72E-03
Clo1313_0800	GCN5-related N-acetyltransferase	-0.1	6.71E-01
Clo1313_0801	hypothetical protein	-0.3	1.85E-01
Clo1313_0802	regulatory protein MarR	-1.0	1.09E-10
Clo1313_0803	MATE efflux family protein	-0.8	6.48E-05
Clo1313_0804	hypothetical protein	-0.8	8.54E-10
Clo1313_0805	TIR protein	-0.4	1.14E-04
Clo1313_0806	Pseudo gene	-0.1	8.99E-01
Clo1313_0807	hypothetical protein	0.7	8.69E-06
Clo1313_0808	Pseudo gene	0.4	1.17E-02
Clo1313_0809	hypothetical protein	0.0	9.31E-01
Clo1313_0810	Pseudo gene	0.0	9.68E-01
Clo1313_0811	RNA polymerase, sigma subunit, SigV	-0.1	8.75E-01
Clo1313_0812	Domain of unknown function DUF3298	-0.3	3.20E-01
Clo1313_0813	Phosphoglycerate mutase	-0.1	7.97E-01
Clo1313_0814	hypothetical protein	-0.1	8.05E-01
Clo1313_0815	short-chain dehydrogenase/reductase SDR	-0.9	1.76E-16
Clo1313_0816	transposase IS200-family protein	-0.6	4.97E-02
Clo1313_0817	hypothetical protein	0.2	1.38E-02
Clo1313_0818	hypothetical protein	0.6	4.55E-02
Clo1313_0819	hypothetical protein	-0.1	8.06E-01

Clo1313_0820	glycosyl hydrolase family 1	0.2	4.76E-01
Clo1313_0821	branched-chain amino acid transport	0.3	3.73E-01
Clo1313_0822	AzlC family protein	0.4	1.09E-01
Clo1313_0823	Inorganic diphosphatase	0.3	1.34E-02
Clo1313_0824	hypothetical protein	0.1	3.27E-01
Clo1313_0825	protein of unknown function UPF0118	0.1	2.64E-01
Clo1313_0826	hypothetical protein	NA	NA
Clo1313_0827	transposase IS200-family protein	NA	NA
Clo1313_0828	RDD domain containing protein	-0.1	7.17E-01
Clo1313_0829	signal peptide peptidase SppA, 36K type	-0.2	4.72E-02
Clo1313_0830	protein of unknown function DUF204	0.2	2.25E-01
Clo1313_0831	hypothetical protein	0.2	6.08E-01
Clo1313_0832	hypothetical protein	0.5	4.27E-03
Clo1313_0833	ABC transporter related protein	-0.1	8.04E-01
Clo1313_0834	hypothetical protein	-0.1	8.43E-01
Clo1313_0835	hypothetical protein	0.0	9.86E-01
Clo1313_0836	tryptophan synthase, beta chain (EC 4.2.1.20)	-0.5	2.07E-03
Clo1313_0837	tryptophan synthase, alpha chain (EC 4.2.1.20)	-0.5	3.27E-06
Clo1313_0838	cation diffusion facilitator family transporter	-0.5	1.39E-03
Clo1313_0839	integral membrane sensor signal transduction histidine kinase	0.4	2.16E-01
Clo1313_0840	G protein-coupled receptor 119	0.2	3.65E-01
Clo1313_0841	two component transcriptional regulator, winged helix family	0.1	8.60E-01
Clo1313_0842	VTC domain	-0.1	8.98E-01
Clo1313_0843	hypothetical protein	-0.3	4.09E-01
Clo1313_0844	Spore coat protein CotH	-0.4	8.69E-03
Clo1313_0845	Ferrous iron transport protein B domain-containing protein	-0.1	6.99E-01
Clo1313_0846	FeoA family protein	0.2	4.19E-01
Clo1313_0847	iron (metal) dependent repressor, DtxR family	0.3	1.83E-01
Clo1313_0848	hypothetical protein	-0.1	4.63E-01
Clo1313_0849	glycosyl hydrolase 53 domain protein	0.1	6.63E-01
Clo1313_0850	C_GCAxxG_C_C family protein	0.5	3.15E-04
Clo1313_0851	Dockerin type 1	-0.7	1.52E-06
Clo1313_0852	glycosyl transferase family 8	0.1	5.05E-01
Clo1313_0853	phospholipase D/Transphosphatidylase	0.2	3.26E-01
Clo1313_0854	Pseudo gene	0.1	7.30E-01
Clo1313_0855	two component transcriptional regulator, winged helix family	-0.2	3.34E-01
Clo1313_0856	multi-sensor signal transduction histidine kinase	0.0	8.94E-01
Clo1313_0857	2-isopropylmalate synthase (EC 2.3.3.13)	-0.6	3.09E-10
Clo1313_0858	alpha/beta hydrolase fold protein	-0.1	6.74E-01
Clo1313_0859	polynucleotide adenylyltransferase/metal dependent phosphohydrolase	-0.2	2.48E-01
Clo1313_0860	Trp operon repressor family	0.2	3.71E-01
Clo1313_0861	hypothetical protein	0.0	9.45E-01
Clo1313_0862	hypothetical protein	0.0	9.98E-01
Clo1313_0863	protein translocase subunit secA	-0.2	5.83E-02
Clo1313_0864	FolC bifunctional protein	-0.1	5.34E-01
Clo1313_0865	Tetratricopeptide TPR_1 repeat-containing protein	-0.1	3.02E-01
Clo1313_0866	Patatin	-0.4	3.26E-04
Clo1313_0867	L-threonine synthase (EC 4.2.3.1)	-0.1	1.36E-01
Clo1313_0868	response regulator receiver protein	0.1	5.21E-01
Clo1313_0869	oxidoreductase domain protein	-0.1	7.01E-01
Clo1313_0870	response regulator receiver modulated metal dependent phosphohydrolase	0.5	1.73E-04
Clo1313_0871	amino acid-binding ACT domain protein	-0.2	2.61E-01
Clo1313_0872	homoserine dehydrogenase (EC 1.1.1.3)	-0.7	1.85E-10
Clo1313_0873	aspartate kinase (EC 2.7.2.4)	-0.6	2.25E-09
Clo1313_0874	copper amine oxidase-like domain-containing protein	1.2	1.41E-06
Clo1313_0875	YD repeat protein	0.5	4.90E-06
Clo1313_0876	hypothetical protein	0.1	6.79E-01
Clo1313_0877	hypothetical protein	0.3	4.71E-02
Clo1313_0878	hypothetical protein	0.2	6.36E-01
Clo1313_0879	hypothetical protein	0.0	9.38E-01
Clo1313_0880	YD repeat protein	-0.2	9.34E-02
Clo1313_0881	hypothetical protein	-0.6	4.03E-06
Clo1313_0882	hypothetical protein	-0.2	6.04E-01
Clo1313_0883	histidine triad (HIT) protein	0.3	4.45E-01
Clo1313_0884	S-layer domain-containing protein	0.2	1.55E-01
Clo1313_0885	transposase IS200-family protein	-0.4	1.90E-01
Clo1313_0886	Protein-tyrosine-phosphatase	0.4	1.05E-02
Clo1313_0887	dTDP-4-dehydrorhamnose 3,5-epimerase	0.7	3.84E-08
Clo1313_0888	hypothetical protein	0.3	2.21E-02
Clo1313_0889	hypothetical protein	-0.2	9.78E-02
Clo1313_0890	capsular exopolysaccharide family	-0.3	2.16E-03
Clo1313_0891	NusG antitermination factor	-0.2	6.25E-02
Clo1313_0892	NAD-dependent epimerase/dehydratase	-0.1	4.95E-01
Clo1313_0893	Oligosaccharide biosynthesis protein Alg14 like protein	-0.3	5.87E-03
Clo1313_0894	Glycosyltransferase 28 domain	-0.4	2.98E-05
Clo1313_0895	glycosyltransferase	-0.3	1.69E-04

Clo1313_0896	glycosyl transferase group 1	-0.5	1.84E-06
Clo1313_0897	glycosyltransferase	-0.2	7.53E-03
Clo1313_0898	glycosyl transferase family 2	-0.2	5.89E-02
Clo1313_0899	O-antigen polymerase	-0.2	2.22E-01
Clo1313_0900	polysaccharide biosynthesis protein	0.0	9.56E-01
Clo1313_0901	UDP-glucose dehydrogenase	-0.2	2.30E-03
Clo1313_0902	VanZ family protein	-0.2	2.74E-01
Clo1313_0903	single-strand binding protein	0.0	7.67E-01
Clo1313_0904	exopolysaccharide biosynthesis polyprenyl glycosylphosphotransferase	0.2	2.95E-02
Clo1313_0905	SSU ribosomal protein S21P	-0.4	6.30E-03
Clo1313_0906	GatB/YqeY domain protein	-0.7	7.20E-22
Clo1313_0907	exonuclease RecJ (EC 3.1.-.-)	-0.1	4.53E-01
Clo1313_0908	adenine phosphoribosyltransferase (EC 2.4.2.7)	-0.2	1.56E-01
Clo1313_0909	(p)ppGpp synthetase I, SpoT/RelA	-0.3	2.71E-03
Clo1313_0910	D-tyrosyl-tRNA(Tyr) deacylase	-0.3	2.68E-01
Clo1313_0911	beta-lactamase-like protein	-0.1	4.10E-01
Clo1313_0912	Coproporphyrinogen dehydrogenase	-0.2	1.05E-01
Clo1313_0913	hypothetical protein	0.6	7.60E-09
Clo1313_0914	type II secretion system protein E	0.7	6.59E-05
Clo1313_0915	Type II secretion system F domain	0.6	7.51E-04
Clo1313_0916	Type II secretion system F domain	0.4	7.57E-02
Clo1313_0917	hypothetical protein	1.1	1.23E-11
Clo1313_0918	hypothetical protein	0.6	9.19E-09
Clo1313_0919	peptidase A24A prepilin type IV	0.7	3.53E-06
Clo1313_0920	Forkhead-associated protein	0.4	1.35E-02
Clo1313_0921	hypothetical protein	-0.5	4.04E-04
Clo1313_0922	histidyl-tRNA synthetase (EC 6.1.1.21)	0.1	4.89E-01
Clo1313_0923	aspartyl-tRNA synthetase (EC 6.1.1.12)	0.0	8.75E-01
Clo1313_0924	signal peptidase I	-0.1	3.00E-01
Clo1313_0925	CoA-substrate-specific enzyme activase	0.1	5.40E-01
Clo1313_0926	hypothetical protein	0.3	4.87E-01
Clo1313_0927	stage II sporulation protein P	0.8	2.24E-02
Clo1313_0928	conserved hypothetical protein	-0.2	6.92E-01
Clo1313_0929	GTP-binding protein LepA	-0.1	6.89E-01
Clo1313_0930	oxygen-independent coproporphyrinogen III oxidase	-0.1	7.71E-01
Clo1313_0931	heat-inducible transcription repressor HrcA	-0.4	1.76E-04
Clo1313_0932	GrpE protein	-0.1	7.95E-01
Clo1313_0933	chaperone protein DnaK	-0.2	1.91E-01
Clo1313_0934	chaperone protein DnaJ	0.3	1.46E-02
Clo1313_0935	[LSU ribosomal protein L11P]-lysine N-methyltransferase (EC 2.1.1.-)	0.1	5.79E-01
Clo1313_0936	protein of unknown function DUF558	0.2	2.85E-01
Clo1313_0937	response regulator receiver protein	-0.1	7.35E-01
Clo1313_0938	hypothetical protein	0.2	5.70E-01
Clo1313_0939	YicC-like domain-containing protein	-0.1	6.70E-01
Clo1313_0940	protein of unknown function DUF370	-0.1	3.21E-01
Clo1313_0941	guanylate kinase (EC 2.7.4.8)	0.0	9.10E-01
Clo1313_0942	DNA-directed RNA polymerase subunit omega (EC 2.7.7.6)	0.0	8.58E-01
Clo1313_0943	Phosphopantothenate-cysteine ligase (EC 6.3.2.5)/Phosphopantothenoylcysteine decarboxylase (EC 4.1.1.36)	0.0	7.92E-01
Clo1313_0944	glycyl-tRNA synthetase (EC 6.1.1.14)	-0.2	3.21E-02
Clo1313_0945	signal transduction histidine kinase regulating citrate/malate metabolism	-0.3	6.64E-04
Clo1313_0946	hypothetical protein	0.3	4.03E-01
Clo1313_0947	Accessory gene regulator B	0.2	6.18E-01
Clo1313_0948	Radical SAM domain protein	0.6	3.44E-02
Clo1313_0949	pyruvate phosphate dikinase (EC 2.7.9.1)	0.3	7.06E-04
Clo1313_0950	cellulosome anchoring protein cohesin region	-0.2	2.98E-03
Clo1313_0951	hypothetical protein	0.9	2.50E-11
Clo1313_0952	Cupin 2 conserved barrel domain protein	0.4	1.84E-02
Clo1313_0953	PhoH family protein	-0.1	7.46E-01
Clo1313_0954	glycosyl transferase group 1	-0.1	5.34E-01
Clo1313_0955	RNA-metabolising metallo-beta-lactamase	-0.3	1.23E-03
Clo1313_0956	protein of unknown function DUF795	0.2	5.84E-01
Clo1313_0957	hypothetical protein	0.0	9.44E-01
Clo1313_0958	hypothetical protein	0.0	9.78E-01
Clo1313_0959	Ribosomal protein L25-like protein	0.0	9.66E-01
Clo1313_0960	nicotinate-nucleotide-dimethylbenzimidazole phosphoribosyltransferase (EC 2.4.2.21)	-0.3	1.73E-01
Clo1313_0961	hypothetical protein	0.1	5.33E-01
Clo1313_0962	5-formyltetrahydrofolate cyclo-ligase	0.2	2.70E-01
Clo1313_0963	hypothetical protein	-0.1	5.92E-01
Clo1313_0964	Recombinase	-0.6	1.37E-04
Clo1313_0965	metal dependent phosphohydrolase	0.3	3.77E-02
Clo1313_0966	phosphoglycerate mutase (EC 5.4.2.1)	-0.3	2.89E-03

Clo1313_0967	amidohydrolase 2	-0.1	5.89E-01
Clo1313_0968	hypothetical protein	0.1	6.61E-01
Clo1313_0969	hypothetical protein	0.4	7.47E-03
Clo1313_0970	two component transcriptional regulator, winged helix family	0.0	9.24E-01
Clo1313_0971	integral membrane sensor signal transduction histidine kinase	0.2	2.00E-01
Clo1313_0972	peptidase S1 and S6 chymotrypsin/Hap	0.1	5.73E-01
Clo1313_0973	metal dependent phosphohydrolase	0.5	2.05E-02
Clo1313_0974	glycogen synthase (ADP-glucose)	-0.3	4.79E-03
Clo1313_0975	DNA-(apurinic or apyrimidinic site) lyase (EC 4.2.99.18)/endonuclease III (EC 3.2.2.-)	0.0	7.97E-01
Clo1313_0976	diguanylate cyclase with GAF sensor	0.5	4.04E-03
Clo1313_0977	hypothetical protein	0.9	9.95E-04
Clo1313_0978	transglycosylase-associated protein	0.5	1.07E-01
Clo1313_0979	LSU ribosomal protein L28P	0.2	5.81E-02
Clo1313_0980	ATP-dependent DNA helicase RecG (EC 3.6.1.-)	-0.3	8.69E-02
Clo1313_0981	methyltransferase	-0.4	6.64E-04
Clo1313_0982	Phosphopantetheine adenylyltransferase (EC 2.7.7.3)	0.3	2.55E-02
Clo1313_0983	H+-ATPase subunit H	0.2	2.70E-02
Clo1313_0984	sporulation integral membrane protein YlbJ	0.6	8.15E-07
Clo1313_0985	alpha-L-arabinofuranosidase B	0.3	1.18E-01
Clo1313_0986	RNA polymerase, sigma 28 subunit, FliA/WhiG subfamily	0.3	1.97E-01
Clo1313_0987	Carbohydrate binding family 6	0.1	4.61E-01
Clo1313_0988	proteinase inhibitor I4 serpin	0.0	9.31E-01
Clo1313_0989	iron-sulfur cluster-binding protein	0.1	5.24E-01
Clo1313_0990	DegS sensor signal transduction histidine kinase	0.5	5.55E-03
Clo1313_0991	two component transcriptional regulator, LuxR family	0.5	9.09E-02
Clo1313_0992	methyl-accepting chemotaxis sensory transducer with Cache sensor	-0.2	2.41E-01
Clo1313_0993	alpha-phosphoglucomutase (EC 5.4.2.2)	-0.1	3.56E-01
Clo1313_0994	DNA polymerase III catalytic subunit, DnaE type	0.0	7.57E-01
Clo1313_0995	tryptophan RNA-binding attenuator protein	0.6	7.85E-04
Clo1313_0996	hypothetical protein	0.3	3.90E-03
Clo1313_0997	6-phosphofructokinase (EC 2.7.1.11)	0.0	8.58E-01
Clo1313_0998	thioesterase superfamily protein	0.1	6.04E-01
Clo1313_0999	CDP-alcohol phosphatidyltransferase	0.8	4.13E-09
Clo1313_1000	copper amine oxidase-like domain-containing protein	0.8	1.06E-09
Clo1313_1001	Carbohydrate-binding CenC domain protein	0.8	1.44E-20
Clo1313_1002	glycoside hydrolase family 3 domain protein	0.7	3.70E-08
Clo1313_1003	hypothetical protein	0.4	2.62E-01
Clo1313_1004	hypothetical protein	-0.1	8.65E-01
Clo1313_1005	pyruvate phosphate dikinase PEP/pyruvate-binding protein	0.2	2.36E-01
Clo1313_1006	Auxin Efflux Carrier	-0.3	7.05E-02
Clo1313_1007	Xanthine/uracil/vitamin C permease	-0.5	4.49E-08
Clo1313_1008	5-(carboxyamino)imidazole ribonucleotide mutase	-0.7	1.19E-05
Clo1313_1009	amidophosphoribosyltransferase (EC 2.4.2.14)	-0.6	1.33E-05
Clo1313_1010	phosphoribosylformylglycinamidine cyclo-ligase (EC 6.3.3.1)	-0.4	9.60E-03
Clo1313_1011	formyltetrahydrofolate-dependent phosphoribosylglycinamide formyltransferase (EC 2.1.2.2)	-0.4	1.79E-02
Clo1313_1012	phosphoribosylaminoimidazolecarboxamide formyltransferase/IMP cyclohydrolase	-0.4	2.80E-03
Clo1313_1013	phosphoribosylamine--glycine ligase (EC 6.3.4.13)	-0.3	3.68E-03
Clo1313_1014	glycosyl transferase family 2	0.3	8.19E-02
Clo1313_1015	GCN5-related N-acetyltransferase	-0.1	9.08E-01
Clo1313_1016	nicotinate-nucleotide adenylyltransferase (EC 2.7.7.18)	-0.4	2.60E-03
Clo1313_1017	metal dependent phosphohydrolase	-0.5	9.13E-04
Clo1313_1018	hypothetical protein	-0.6	6.21E-06
Clo1313_1019	iojap-like protein	0.3	1.34E-01
Clo1313_1020	leucyl-tRNA synthetase (EC 6.1.1.4)	-0.1	2.94E-01
Clo1313_1021	PKD domain containing protein	-0.1	4.28E-01
Clo1313_1022	methyl-accepting chemotaxis sensory transducer	0.4	3.10E-01
Clo1313_1023	hypothetical protein	0.8	1.50E-02
Clo1313_1024	AMP-dependent synthetase and ligase	0.2	6.05E-02
Clo1313_1025	peptidase S11 D-alanyl-D-alanine carboxypeptidase 1	0.5	2.43E-07
Clo1313_1026	competence protein ComEA helix-hairpin-helix repeat protein	0.0	9.15E-01
Clo1313_1027	Lipoprotein LpqB, GerMN domain	0.1	4.88E-01
Clo1313_1028	hypothetical protein	0.2	6.02E-01
Clo1313_1029	threonyl-tRNA synthetase (EC 6.1.1.3)	0.0	8.34E-01
Clo1313_1030	thymidylate synthase (EC 2.1.1.45)	0.1	6.30E-01
Clo1313_1031	dihydrofolate reductase region	-0.1	6.65E-01
Clo1313_1032	bacterial translation initiation factor 3 (bIF-3)	-0.6	3.82E-10
Clo1313_1033	LSU ribosomal protein L35P	-0.5	2.80E-03
Clo1313_1034	LSU ribosomal protein L20P	-0.3	2.32E-03
Clo1313_1035	RNA methyltransferase, TrmH family, group 3	-0.1	7.66E-01
Clo1313_1036	glycosyltransferase 36	-0.1	4.46E-01
Clo1313_1037	hypothetical protein	0.3	1.48E-02
Clo1313_1038	hypothetical protein	0.3	4.29E-02
Clo1313_1039	leucyl/phenylalanyl-tRNA/protein transferase	0.5	3.08E-02

Clo1313_1040	ATP-dependent Clp protease ATP-binding subunit ClpA (EC 3.4.21.92)	0.0	7.31E-01
Clo1313_1041	ATP-dependent Clp protease adaptor protein ClpS	-0.3	2.00E-01
Clo1313_1042	protein of unknown function DUF81	-0.1	8.62E-01
Clo1313_1043	protein of unknown function DUF81	0.1	7.56E-01
Clo1313_1044	hypothetical protein	0.3	4.87E-01
Clo1313_1045	hypothetical protein	0.2	9.61E-02
Clo1313_1046	pyridoxal-phosphate dependent TrpB-like enzyme	0.3	7.25E-05
Clo1313_1047	hypothetical protein	0.0	9.13E-01
Clo1313_1048	hypothetical protein	0.3	2.71E-01
Clo1313_1049	GCN5-related N-acetyltransferase	0.4	1.87E-01
Clo1313_1050	hypothetical protein	0.1	8.04E-01
Clo1313_1051	hypothetical protein	0.2	6.62E-02
Clo1313_1052	putative serine protein kinase, PrkA	-0.3	3.75E-04
Clo1313_1053	sporulation protein YhbH	0.2	1.92E-01
Clo1313_1054	SpoVR family protein	0.6	1.56E-02
Clo1313_1055	major facilitator superfamily MFS_1	0.7	3.51E-11
Clo1313_1056	purine nucleoside phosphorylase I, inosine and guanosine-specific	0.4	1.18E-03
Clo1313_1057	adenosylhomocysteinase (EC 3.3.1.1)	-0.1	6.08E-01
Clo1313_1058	amidohydrolase	0.0	7.35E-01
Clo1313_1059	hypothetical protein	0.8	2.57E-04
Clo1313_1060	Hedgehog/intein hint domain protein	0.4	4.55E-02
Clo1313_1061	Pseudo gene	0.3	3.44E-02
Clo1313_1062	Pseudo gene	0.4	3.66E-02
Clo1313_1063	Pseudo gene	0.1	7.23E-01
Clo1313_1064	Pseudo gene	-0.1	6.90E-01
Clo1313_1065	Domain of unknown function DUF1910	0.1	4.66E-01
Clo1313_1066	transposase mutator type	NA	NA
Clo1313_1067	hypothetical protein	-0.4	2.17E-02
Clo1313_1068	HAD-superfamily hydrolase, subfamily IA, variant 1	0.2	6.95E-01
Clo1313_1069	hypothetical protein	-0.2	7.05E-01
Clo1313_1070	ABC transporter related protein	-0.1	8.39E-01
Clo1313_1071	protein of unknown function DUF990	0.0	9.03E-01
Clo1313_1072	protein of unknown function DUF990	-0.1	6.44E-01
Clo1313_1073	esterase/lipase	0.2	3.55E-01
Clo1313_1074	hypothetical protein	0.2	1.92E-01
Clo1313_1075	hypothetical protein	0.0	9.94E-01
Clo1313_1076	ATPase associated with various cellular activities AAA_3	0.1	7.72E-01
Clo1313_1077	protein of unknown function DUF58	0.2	2.41E-01
Clo1313_1078	transglutaminase domain-containing protein	0.2	2.56E-01
Clo1313_1079	hypothetical protein	0.4	1.96E-02
Clo1313_1080	small acid-soluble spore protein, H-type	0.2	7.10E-01
Clo1313_1081	isochorismatase hydrolase	-0.1	8.36E-01
Clo1313_1082	nicotinate phosphoribosyltransferase	-0.2	4.01E-01
Clo1313_1083	CheC domain protein	0.5	1.26E-04
Clo1313_1084	protein of unknown function DUF155	0.4	2.37E-01
Clo1313_1085	protein of unknown function DUF155	0.7	4.95E-03
Clo1313_1086	type IV pilus assembly PilZ	0.2	2.65E-01
Clo1313_1087	iron-sulfur cluster-binding protein	0.4	3.07E-04
Clo1313_1088	hypothetical protein	0.0	9.98E-01
Clo1313_1089	peptidase S11 D-alanyl-D-alanine carboxypeptidase 1	0.7	3.11E-10
Clo1313_1090	dihydrodipicolinate reductase (EC 1.3.1.26)	0.5	6.81E-03
Clo1313_1091	hypothetical protein	0.1	6.39E-01
Clo1313_1092	hypothetical protein	0.1	5.40E-01
Clo1313_1093	AMMECR1 domain protein	0.4	5.40E-02
Clo1313_1094	Radical SAM domain protein	0.6	4.65E-03
Clo1313_1095	protein of unknown function DUF147	0.8	1.30E-17
Clo1313_1096	YbbR family protein	0.8	7.96E-19
Clo1313_1097	FAD-dependent pyridine nucleotide-disulfide oxidoreductase	0.6	3.62E-07
Clo1313_1098	phosphoglucosamine mutase (EC 5.4.2.10)	0.2	2.22E-01
Clo1313_1099	glutamine--fructose-6-phosphate transaminase	0.2	5.72E-03
Clo1313_1100	transcriptional regulator, ArsR family	-0.1	7.42E-01
Clo1313_1101	arsenical-resistance protein	0.1	8.82E-01
Clo1313_1102	Protein-tyrosine phosphatase, low molecular weight	-0.4	1.12E-01
Clo1313_1103	permease	0.2	5.54E-01
Clo1313_1104	transposase IS3/IS911 family protein	NA	NA
Clo1313_1105	hypothetical protein	0.0	9.56E-01
Clo1313_1106	type II secretion system protein E	0.2	1.64E-02
Clo1313_1107	twitching motility protein	0.2	1.85E-02
Clo1313_1108	Type II secretion system F domain	0.5	8.03E-09
Clo1313_1109	hypothetical protein	0.4	1.82E-05
Clo1313_1110	hypothetical protein	0.1	3.50E-01
Clo1313_1111	Fimbrial assembly family protein	-0.1	6.51E-01
Clo1313_1112	hypothetical protein	-0.2	9.60E-03
Clo1313_1113	hypothetical protein	-0.1	3.35E-01
Clo1313_1114	hypothetical protein	0.0	8.99E-01
Clo1313_1115	hypothetical protein	0.2	9.10E-03

Clo1313_1116	ATP-dependent Clp protease proteolytic subunit ClpP (EC 3.4.21.92)	0.0	9.02E-01
Clo1313_1117	hypothetical protein	0.5	1.16E-01
Clo1313_1118	DNA translocase FtsK	-0.1	6.05E-01
Clo1313_1119	Radical SAM domain protein	0.2	5.40E-02
Clo1313_1120	Methylenetetrahydrofolate dehydrogenase (NADP(+))	0.4	1.34E-08
Clo1313_1121	hypothetical protein	0.0	7.86E-01
Clo1313_1122	metal dependent phosphohydrolase	-0.1	1.34E-01
Clo1313_1123	metallophosphoesterase	0.1	5.49E-01
Clo1313_1124	Stage V sporulation protein S	0.2	1.41E-01
Clo1313_1125	hypothetical protein	0.1	6.22E-01
Clo1313_1126	hypothetical protein	-0.1	8.40E-01
Clo1313_1127	dTDP-glucose 4,6-dehydratase (EC 4.2.1.46)	0.4	3.47E-01
Clo1313_1128	glycosyl transferase group 1	-0.1	7.75E-01
Clo1313_1129	spore coat protein, CotS family	0.3	4.87E-01
Clo1313_1130	spore coat protein, CotS family	-0.3	4.03E-01
Clo1313_1131	transcription initiation factor IIE (TFIIE) alpha subunit family protein	0.1	8.11E-01
Clo1313_1132	hypothetical protein	-0.2	6.09E-01
Clo1313_1133	hypothetical protein	0.0	8.98E-01
Clo1313_1134	Nucleotidyl transferase	-0.5	4.16E-03
Clo1313_1135	hypothetical protein	-0.6	8.83E-06
Clo1313_1136	hypothetical protein	0.2	6.27E-01
Clo1313_1137	protein of unknown function DUF881	0.3	9.48E-03
Clo1313_1138	protein of unknown function DUF881	0.5	1.12E-06
Clo1313_1139	delta-lactam-biosynthetic de-N-acetylase	0.3	3.98E-01
Clo1313_1140	sporulation protein YqfC	0.3	5.49E-01
Clo1313_1141	sporulation protein YqfD	-0.1	5.86E-01
Clo1313_1142	PhoH family protein	-0.2	8.55E-02
Clo1313_1143	7TM receptor with intracellular metal dependent phosphohydrolase	-0.2	1.85E-02
Clo1313_1144	protein of unknown function UPF0054	-0.3	2.92E-02
Clo1313_1145	cytidine deaminase (EC 3.5.4.5)	-0.1	5.88E-01
Clo1313_1146	GTP-binding protein Era	0.0	9.93E-01
Clo1313_1147	hypothetical protein	0.1	8.40E-01
Clo1313_1148	DNA replication and repair protein RecO	0.2	2.15E-02
Clo1313_1149	type IV pilus assembly PilZ	0.1	3.48E-01
Clo1313_1150	Cysteine desulfurase	0.0	8.43E-01
Clo1313_1151	thiamine biosynthesis/tRNA modification protein ThiI	0.0	8.75E-01
Clo1313_1152	VanW family protein	-0.2	1.46E-01
Clo1313_1153	Protein of unknown function DUF2225	0.6	1.25E-02
Clo1313_1154	AIR synthase related protein domain protein	0.5	6.95E-05
Clo1313_1155	serine hydroxymethyltransferase (EC 2.1.2.1)	-2.4	2.61E-188
Clo1313_1156	phage shock protein C (PspC) family protein	-0.7	4.03E-02
Clo1313_1157	transglutaminase-domain-containing protein	-0.1	6.65E-01
Clo1313_1158	protein of unknown function DUF58	0.1	4.97E-01
Clo1313_1159	ATPase associated with various cellular activities AAA_3	0.0	9.95E-01
Clo1313_1160	L-lactate dehydrogenase (EC 1.1.1.27)	0.3	5.14E-01
Clo1313_1161	competence/damage-inducible protein cinA	0.0	9.66E-01
Clo1313_1162	integral membrane protein MviN	0.2	3.52E-01
Clo1313_1163	recA protein	0.3	1.08E-02
Clo1313_1164	regulatory protein RecX	-0.1	7.75E-01
Clo1313_1165	Rhomboid family protein	0.1	4.97E-01
Clo1313_1166	hypothetical protein	0.4	3.08E-03
Clo1313_1167	carbohydrate ABC transporter substrate-binding protein, CUT1 family (TC 3.A.1.1.-)	0.9	4.97E-11
Clo1313_1168	hypothetical protein	0.1	7.91E-01
Clo1313_1169	hypothetical protein	-0.1	6.95E-01
Clo1313_1170	hypothetical protein	0.0	9.68E-01
Clo1313_1171	hypothetical protein	-0.1	7.53E-01
Clo1313_1172	UDP-N-acetylmuramoylalanine--D-glutamate ligase (EC 6.3.2.9)	0.2	5.61E-02
Clo1313_1173	DNA polymerase III, delta subunit (EC 2.7.7.7)	0.1	4.62E-01
Clo1313_1174	SSU ribosomal protein S20P	-0.4	4.69E-04
Clo1313_1175	spore protease	0.3	4.42E-01
Clo1313_1176	cell wall hydrolase/autolysin	-0.1	7.01E-01
Clo1313_1177	hypothetical protein	0.4	1.51E-02
Clo1313_1178	DNA ligase, NAD-dependent	0.1	5.36E-01
Clo1313_1179	hypothetical protein	0.2	6.39E-01
Clo1313_1180	hypothetical protein	0.3	1.67E-02
Clo1313_1181	aspartyl/glutamyl-tRNA(Asn/Gln) amidotransferase subunit C (EC 6.3.5.-)	0.0	9.90E-01
Clo1313_1182	aspartyl/glutamyl-tRNA(Asn/Gln) amidotransferase subunit A (EC 6.3.5.-)	-0.1	5.34E-01
Clo1313_1183	aspartyl/glutamyl-tRNA(Asn/Gln) amidotransferase subunit B (EC 6.3.5.-)	-0.1	7.29E-01
Clo1313_1184	Tetratricopeptide TPR_1 repeat-containing protein	0.2	5.69E-02
Clo1313_1185	phosphate acetyltransferase	0.4	8.56E-04

Clo1313_1186	acetate kinase (EC 2.7.2.1)	0.4	1.46E-05
Clo1313_1187	protein of unknown function DUF177	-0.1	5.96E-01
Clo1313_1188	ribosomal protein L32	0.1	6.30E-01
Clo1313_1189	protein of unknown function DUF512	0.0	8.44E-01
Clo1313_1190	ribosome-associated GTPase EngA	-0.2	1.08E-01
Clo1313_1191	acyl-phosphate glycerol-3-phosphate acyltransferase	-0.1	4.63E-01
Clo1313_1192	glycerol 3-phosphate dehydrogenase (NAD(P)+) (EC 1.1.1.94)	-0.2	1.20E-01
Clo1313_1193	stage IV sporulation protein A	0.1	7.73E-01
Clo1313_1194	extracellular solute-binding protein family 1	-0.1	3.27E-01
Clo1313_1195	binding-protein-dependent transport systems inner membrane component	-0.1	7.14E-01
Clo1313_1196	binding-protein-dependent transport systems inner membrane component	-0.2	1.70E-01
Clo1313_1197	hypothetical protein	0.0	9.78E-01
Clo1313_1198	cell wall hydrolase/autolysin	-0.5	1.22E-03
Clo1313_1199	hypothetical protein	-0.5	2.77E-05
Clo1313_1200	hypothetical protein	0.4	3.63E-01
Clo1313_1201	MutS2 family protein	-0.2	7.37E-02
Clo1313_1202	sporulation integral membrane protein YtvI	0.5	2.59E-03
Clo1313_1203	RNA polymerase, sigma 27/28 subunit, RpsK/SigK	0.7	4.97E-02
Clo1313_1204	Peptidoglycan glycosyltransferase	0.7	1.20E-03
Clo1313_1205	peptidase U32	-0.2	5.58E-02
Clo1313_1206	O-methyltransferase family 3	0.0	9.48E-01
Clo1313_1207	aminodeoxychorismate lyase	-0.4	1.69E-04
Clo1313_1208	GTP-binding protein TypA	-0.2	1.35E-02
Clo1313_1209	ribosomal protein S2	-0.3	2.51E-02
Clo1313_1210	translation elongation factor Ts	-0.2	1.21E-02
Clo1313_1211	uridylate kinase (EC 2.7.4.22)	0.1	7.83E-01
Clo1313_1212	ribosome recycling factor	-0.2	5.96E-02
Clo1313_1213	hypothetical protein	0.1	8.04E-01
Clo1313_1214	undecaprenyl diphosphate synthase	0.1	3.06E-01
Clo1313_1215	phosphatidate cytidylyltransferase	0.1	6.92E-01
Clo1313_1216	1-deoxy-D-xylulose 5-phosphate reductoisomerase (EC 1.1.1.267)	0.1	5.77E-01
Clo1313_1217	membrane-associated zinc metalloprotease	0.0	9.36E-01
Clo1313_1218	4-hydroxy-3-methylbut-2-en-1-yl diphosphate synthase (EC 1.17.4.3)	-0.1	6.89E-01
Clo1313_1219	DNA polymerase III, alpha subunit	0.2	4.97E-02
Clo1313_1220	hypothetical protein	-0.1	6.47E-01
Clo1313_1221	protein of unknown function DUF150	-0.3	7.22E-03
Clo1313_1222	NusA antitermination factor	-0.3	5.66E-04
Clo1313_1223	protein of unknown function DUF448	0.2	3.05E-01
Clo1313_1224	LSU ribosomal protein L7AE	-0.2	2.20E-01
Clo1313_1225	translation initiation factor IF-2	0.0	8.56E-01
Clo1313_1226	ribosome-binding factor A	-0.1	5.33E-01
Clo1313_1227	phosphoesterase RecJ domain protein	0.0	9.63E-01
Clo1313_1228	tRNA pseudouridine synthase B (EC 4.2.1.70)	0.2	1.26E-01
Clo1313_1229	riboflavin biosynthesis protein RibF	0.1	1.44E-01
Clo1313_1230	hypothetical protein	0.0	9.82E-01
Clo1313_1231	peptidase M16 domain protein	0.1	3.57E-01
Clo1313_1232	peptidase M16 domain protein	0.3	3.81E-03
Clo1313_1233	prolipoprotein diacylglyceryl transferase	0.1	2.66E-01
Clo1313_1234	hypothetical protein	1.9	8.12E-21
Clo1313_1235	rod shape-determining protein RodA	0.9	5.64E-15
Clo1313_1236	MraZ protein	0.3	1.64E-02
Clo1313_1237	S-adenosyl-methyltransferase MraW	-0.1	7.42E-01
Clo1313_1238	cell division protein FtsL	0.3	2.62E-02
Clo1313_1239	penicillin-binding protein transpeptidase	0.6	3.90E-11
Clo1313_1240	UDP-N-acetylmuramoylalanyl-D-glutamate--2,6-diaminopimelate ligase (EC 6.3.2.13)	0.6	3.54E-07
Clo1313_1241	UDP-N-acetylmuramoyl-tripeptide--D-alanyl-D-alanine ligase (EC 6.3.2.10)	0.7	7.33E-05
Clo1313_1242	Phospho-N-acetylmuramoyl-pentapeptide-transferase (EC 2.7.8.13)	0.6	2.23E-07
Clo1313_1243	stage V sporulation protein E	0.8	8.30E-08
Clo1313_1244	UDP-N-acetylglucosamine-N-acetylmuramylpentapeptide N-acetylglucosamine transferase	0.7	4.94E-15
Clo1313_1245	UDP-N-acetylglucosamine 1-carboxyvinyltransferase	0.4	1.81E-01
Clo1313_1246	hypothetical protein	0.1	7.26E-01
Clo1313_1247	Pseudo gene	-0.2	3.41E-01
Clo1313_1248	transposase mutator type	NA	NA
Clo1313_1249	UvrD/REP helicase	-0.1	7.32E-01
Clo1313_1250	copper amine oxidase-like domain-containing protein	0.0	9.09E-01
Clo1313_1251	ATP:corrinoid adenosyltransferase BtuR/CobO/CobP	0.6	7.29E-03
Clo1313_1252	aspartate kinase (EC 2.7.2.4)	0.1	3.67E-01
Clo1313_1253	dihydrodipicolinate reductase (EC 1.3.1.26)	0.0	8.21E-01
Clo1313_1254	dihydrodipicolinate synthase (EC 4.2.1.52)	0.2	4.10E-02
Clo1313_1255	aspartate semialdehyde dehydrogenase (EC 1.2.1.11)	0.2	3.77E-02

Clo1313_1256	SpoIID/LytB domain protein	0.9	6.71E-13
Clo1313_1257	S-adenosylmethionine--tRNA ribosyltransferase-isomerase	0.2	6.12E-02
Clo1313_1258	tRNA-guanine transglycosylase (EC 2.4.2.29)	0.3	1.96E-03
Clo1313_1259	protein translocase subunit yajC	0.0	7.28E-01
Clo1313_1260	hypothetical protein	0.2	5.40E-01
Clo1313_1261	hypothetical protein	0.9	1.23E-03
Clo1313_1262	phosphoribosyltransferase	0.1	3.27E-01
Clo1313_1263	Helicase conserved C-terminal domain containing protein-like protein	0.1	8.99E-01
Clo1313_1264	aspartate carbamoyltransferase (EC 2.1.3.2)	0.2	1.25E-01
Clo1313_1265	dihydroorotase (EC 3.5.2.3)	0.2	1.28E-01
Clo1313_1266	orotidine-5'-phosphate decarboxylase (EC 4.1.1.23)	0.1	4.85E-01
Clo1313_1267	carbamoyl-phosphate synthase, small subunit	0.0	8.40E-01
Clo1313_1268	carbamoyl-phosphate synthase large subunit	0.1	7.31E-01
Clo1313_1269	Dihydroorotate dehydrogenase, electron transfer subunit, iron-sulfur cluster binding domain	-0.1	4.94E-01
Clo1313_1270	dihydroorotate dehydrogenase family protein	0.4	8.11E-02
Clo1313_1271	Phosphoglycerate mutase	0.1	5.29E-01
Clo1313_1272	metallophosphoesterase	0.0	8.52E-01
Clo1313_1273	Rad50 zinc hook domain protein	-0.1	3.58E-01
Clo1313_1274	hypothetical protein	-0.2	5.59E-01
Clo1313_1275	SSU ribosomal protein S12P methylthiotransferase (EC 2.-.-.-)	0.0	9.13E-01
Clo1313_1276	CDP-diacylglycerol--glycerol-3-phosphate 3-phosphatidyltransferase (EC 2.7.8.5)	-0.1	7.10E-01
Clo1313_1277	hypothetical protein	-0.1	7.05E-01
Clo1313_1278	hypothetical protein	0.2	6.56E-01
Clo1313_1279	regulatory protein DeoR	0.0	7.96E-01
Clo1313_1280	phosphate:acyl-[acyl carrier protein] acyltransferase	-0.2	1.88E-01
Clo1313_1281	3-oxoacyl-[acyl-carrier-protein] synthase III (EC 2.3.1.41)	-0.3	8.00E-03
Clo1313_1282	[Acyl-carrier-protein] S-malonyltransferase (EC 2.3.1.39)	-0.7	7.59E-10
Clo1313_1283	3-oxoacyl-[acyl-carrier-protein] reductase (EC 1.1.1.100)	-0.9	2.44E-22
Clo1313_1284	acyl carrier protein	0.0	8.25E-01
Clo1313_1285	3-oxoacyl-[acyl-carrier-protein] synthase II (EC 2.3.1.41)	-0.9	3.55E-16
Clo1313_1286	RNAse III (EC 3.1.26.3)	-0.5	9.74E-06
Clo1313_1287	Radical SAM domain protein	-0.5	1.28E-04
Clo1313_1288	Stage V sporulation protein S	0.4	3.94E-02
Clo1313_1289	4-hydroxythreonine-4-phosphate dehydrogenase (EC 1.1.1.262)	0.0	9.06E-01
Clo1313_1290	condensin subunit Smc	0.0	8.51E-01
Clo1313_1291	signal recognition particle-docking protein FtsY	-0.3	1.85E-04
Clo1313_1292	protein of unknown function DUF218	0.0	9.78E-01
Clo1313_1293	hypothetical protein	0.1	7.50E-01
Clo1313_1294	hypothetical protein	0.4	1.17E-01
Clo1313_1295	diaminopimelate dehydrogenase (EC 1.4.1.16)	-0.1	5.11E-01
Clo1313_1296	hypothetical protein	0.3	3.22E-01
Clo1313_1297	fibronectin, type III domain-containing protein	-0.2	1.84E-01
Clo1313_1298	transposase IS3/IS911 family protein	NA	NA
Clo1313_1299	fibronectin, type III domain-containing protein	-0.5	7.55E-04
Clo1313_1300	Dockerin type 1	-0.3	2.33E-04
Clo1313_1301	glutaminyl-tRNA synthetase (EC 6.1.1.18)	-0.2	2.89E-02
Clo1313_1302	hypothetical protein	-0.3	1.34E-02
Clo1313_1303	hypothetical protein	-0.6	1.22E-05
Clo1313_1304	Protein of unknown function DUF2339, transmembrane	-0.3	4.67E-03
Clo1313_1305	glycoside hydrolase family 10	-0.1	4.16E-01
Clo1313_1306	protein of unknown function DUF152	-0.1	3.79E-01
Clo1313_1307	hypothetical protein	0.2	5.79E-01
Clo1313_1308	extracellular solute-binding protein family 5	0.0	8.64E-01
Clo1313_1309	signal peptidase II (EC:3.4.23.36). Aspartic peptidase. MEROPS family A08	-0.1	4.89E-01
Clo1313_1310	ribosomal large subunit pseudouridine synthase D (EC 5.4.99.-)	-0.4	2.83E-04
Clo1313_1311	hypothetical protein	-0.5	1.91E-01
Clo1313_1312	Radical SAM domain protein	0.2	8.25E-02
Clo1313_1313	metal dependent phosphohydrolase	0.0	9.41E-01
Clo1313_1314	protein-export membrane protein SecD	-0.2	2.63E-02
Clo1313_1315	protein translocase subunit secF	0.1	6.95E-01
Clo1313_1316	ketopantoate hydroxymethyltransferase (EC 2.1.2.11)	0.1	6.19E-01
Clo1313_1317	pantoate/beta-alanine ligase	0.3	2.54E-02
Clo1313_1318	L-aspartate 1-decarboxylase (EC 4.1.1.11)	0.3	1.74E-02
Clo1313_1319	hypothetical protein	-0.1	8.05E-01
Clo1313_1320	metal dependent phosphohydrolase	0.8	7.53E-07
Clo1313_1321	deoxyguanosinetriphosphate triphosphohydrolase	0.3	2.49E-01
Clo1313_1322	hypothetical protein	0.6	5.58E-02
Clo1313_1323	DNA primase	0.0	8.51E-01
Clo1313_1324	RNA polymerase, sigma 38 subunit, RpoS	0.0	8.89E-01
Clo1313_1325	hypothetical protein	-0.3	2.94E-01
Clo1313_1326	hypothetical protein	0.1	6.65E-01
Clo1313_1327	protein of unknown function DUF633	0.1	8.04E-01
Clo1313_1328	protein of unknown function DUF34	0.3	1.17E-02
Clo1313_1329	response regulator receiver protein	0.6	2.85E-03

Clo1313_1330	RNA polymerase, sigma-24 subunit, RpoE	0.8	3.08E-07
Clo1313_1331	hypothetical protein	0.5	2.99E-05
Clo1313_1332	type IV pilus assembly PilZ	0.7	4.52E-07
Clo1313_1333	hypothetical protein	0.5	5.78E-02
Clo1313_1334	DNA polymerase I (EC 2.7.7.7)	0.1	2.07E-01
Clo1313_1335	dephospho-CoA kinase	0.1	5.93E-01
Clo1313_1336	Lytic transglycosylase catalytic	-0.1	6.95E-01
Clo1313_1337	regulatory protein, MerR	0.3	1.97E-01
Clo1313_1338	protein of unknown function DUF6 transmembrane	0.2	4.73E-01
Clo1313_1339	diguanylate cyclase	0.8	8.84E-09
Clo1313_1340	3-deoxy-D-arabinoheptulosonate-7-phosphate synthase (EC 2.5.1.54)	-0.3	4.67E-02
Clo1313_1341	CheW protein	0.4	5.38E-05
Clo1313_1342	GTP-binding protein YchF	-0.5	2.40E-10
Clo1313_1343	glycosyl transferase family protein	0.1	5.37E-01
Clo1313_1344	anthranilate synthase component I	0.1	3.01E-01
Clo1313_1345	anthranilate synthase, component II (EC 4.1.3.27)	0.1	6.92E-01
Clo1313_1346	anthranilate phosphoribosyltransferase (EC 2.4.2.18)	-0.3	3.39E-02
Clo1313_1347	indole-3-glycerol phosphate synthase (EC 4.1.1.48)	-0.4	7.08E-03
Clo1313_1348	phosphoribosylanthranilate isomerase (EC 5.3.1.24)	-0.3	1.10E-01
Clo1313_1349	NADPH-dependent FMN reductase	-0.1	2.30E-01
Clo1313_1350	hypothetical protein	-0.1	8.54E-01
Clo1313_1351	hypothetical protein	-0.1	1.85E-01
Clo1313_1352	type IV pilus assembly PilZ	0.4	2.30E-04
Clo1313_1353	4Fe-4S ferredoxin iron-sulfur binding domain protein	0.7	1.28E-02
Clo1313_1354	pyruvate flavodoxin/ferredoxin oxidoreductase domain protein	0.1	5.24E-01
Clo1313_1355	thiamine pyrophosphate TPP-binding domain-containing protein	0.0	7.95E-01
Clo1313_1356	2-oxoglutarate ferredoxin oxidoreductase, gamma subunit (EC 1.2.7.3)	0.0	7.78E-01
Clo1313_1357	glutamine synthetase catalytic region	0.6	5.38E-11
Clo1313_1358	hypothetical protein	0.3	2.43E-01
Clo1313_1359	transcriptional attenuator, LytR family	0.3	1.45E-03
Clo1313_1360	Rubrerythrin	-0.5	2.06E-09
Clo1313_1361	hypothetical protein	0.2	1.51E-01
Clo1313_1362	band 7 protein	0.2	7.29E-02
Clo1313_1363	protein of unknown function DUF107	0.2	5.28E-02
Clo1313_1364	branched-chain amino acid aminotransferase	0.1	6.65E-01
Clo1313_1365	HAD superfamily (subfamily IIIA) phosphatase, TIGR01668	0.6	1.79E-05
Clo1313_1366	shikimate dehydrogenase (EC 1.1.1.25)	0.4	1.91E-02
Clo1313_1367	type II secretion system protein E (GspE)	0.2	3.83E-01
Clo1313_1368	Prepilin peptidase	0.3	4.62E-03
Clo1313_1369	Late competence development protein ComFB	0.2	4.87E-01
Clo1313_1370	Xylose isomerase domain-containing protein TIM barrel	-0.2	3.06E-01
Clo1313_1371	3-dehydroquinate dehydratase (EC 4.2.1.10)	-0.2	6.02E-01
Clo1313_1372	peptidase M24	0.1	3.48E-01
Clo1313_1373	translation elongation factor P (EF-P)	0.3	1.51E-02
Clo1313_1374	hypothetical protein	0.0	8.52E-01
Clo1313_1375	transposase IS200-family protein	NA	NA
Clo1313_1376	stage III sporulation protein AA	0.1	7.83E-01
Clo1313_1377	stage III sporulation protein AB	-0.1	9.02E-01
Clo1313_1378	stage III sporulation protein AC	-0.2	2.94E-01
Clo1313_1379	stage III sporulation protein AD	0.0	9.80E-01
Clo1313_1380	stage III sporulation protein AE	-0.2	6.82E-01
Clo1313_1381	stage III sporulation protein AF	0.4	2.87E-01
Clo1313_1382	stage III sporulation protein AG	-0.4	3.82E-01
Clo1313_1383	hypothetical protein	-0.2	5.10E-01
Clo1313_1384	protein of unknown function DUF322	0.2	4.55E-01
Clo1313_1385	hypothetical protein	0.5	4.53E-07
Clo1313_1386	Protein of unknown function DUF2273	0.0	9.02E-01
Clo1313_1387	NusB antitermination factor	0.4	9.31E-04
Clo1313_1388	Exodeoxyribonuclease VII large subunit (EC 3.1.11.6)	0.3	1.49E-02
Clo1313_1389	Exodeoxyribonuclease VII small subunit (EC 3.1.11.6)	0.5	3.20E-02
Clo1313_1390	Polyprenyl synthetase	0.1	6.48E-01
Clo1313_1391	acid phosphatase/vanadium-dependent haloperoxidase related protein	-0.1	6.38E-01
Clo1313_1392	hypothetical protein	0.3	1.61E-02
Clo1313_1393	1-deoxy-D-xylulose-5-phosphate synthase (EC 2.2.1.7)	0.4	1.22E-04
Clo1313_1394	hemolysin A	0.6	4.92E-05
Clo1313_1395	hypothetical protein	0.3	3.07E-02
Clo1313_1396	non-processive endocellulase	0.9	7.39E-27
Clo1313_1397	copper amine oxidase-like domain-containing protein	0.3	3.85E-02
Clo1313_1398	coagulation factor 5/8 type domain protein	0.8	1.94E-03
Clo1313_1399	hypothetical protein	0.1	8.54E-01
Clo1313_1400	hypothetical protein	-0.2	1.91E-01
Clo1313_1401	ABC transporter related protein	0.1	7.53E-01
Clo1313_1402	hypothetical protein	0.3	3.65E-01
Clo1313_1403	Pseudo gene	0.2	6.36E-01
Clo1313_1404	diguanylate cyclase with TPR repeats	0.7	1.01E-08

Clo1313_1405	ATP-NAD/AcoX kinase	0.2	2.60E-01
Clo1313_1406	transcriptional regulator, ArgR family	0.2	3.57E-01
Clo1313_1407	DNA replication and repair protein RecN	0.0	8.87E-01
Clo1313_1408	SpoIVB peptidase. Serine peptidase. MEROPS family S55	-0.1	6.79E-01
Clo1313_1409	sporulation transcriptional activator Spo0A	0.0	9.37E-01
Clo1313_1410	response regulator receiver protein	1.0	3.02E-03
Clo1313_1411	CheA signal transduction histidine kinase	1.2	3.12E-11
Clo1313_1412	CheW protein	0.7	5.58E-02
Clo1313_1413	MCP methyltransferase, CheR-type	0.8	2.34E-03
Clo1313_1414	response regulator receiver modulated CheB methylesterase	0.8	4.86E-03
Clo1313_1415	PAS/PAC sensor hybrid histidine kinase (EC 2.7.13.3)	0.3	1.18E-01
Clo1313_1416	response regulator receiver protein	0.6	7.00E-02
Clo1313_1417	regulatory protein MerR	0.7	3.74E-02
Clo1313_1418	pseudouridine synthase, RluA family	0.3	1.10E-01
Clo1313_1419	hypothetical protein	-0.1	8.43E-01
Clo1313_1420	hypothetical protein	0.0	8.94E-01
Clo1313_1421	transposase mutator type	0.1	7.48E-01
Clo1313_1422	integral membrane sensor signal transduction histidine kinase	0.6	4.99E-04
Clo1313_1423	two component transcriptional regulator, winged helix family	0.0	9.19E-01
Clo1313_1424	lipolytic protein G-D-S-L family	1.1	9.27E-06
Clo1313_1425	glycoside hydrolase family 5	-0.2	6.00E-01
Clo1313_1426	alpha amylase catalytic region	0.2	3.25E-01
Clo1313_1427	aluminum resistance family protein	0.4	4.76E-03
Clo1313_1428	hypothetical protein	0.0	8.68E-01
Clo1313_1429	alanine racemase domain protein	0.1	5.37E-01
Clo1313_1430	protein of unknown function DUF552	0.1	6.13E-01
Clo1313_1431	protein of unknown function YGGT	-0.4	3.28E-02
Clo1313_1432	RNA-binding S4 domain protein	0.1	4.87E-01
Clo1313_1433	DivIVA domain	-0.1	6.92E-01
Clo1313_1434	Isoleucyl-tRNA synthetase (EC 6.1.1.5)	0.0	9.78E-01
Clo1313_1435	3-dehydroquinate synthase (EC 4.2.3.4)	0.1	7.04E-01
Clo1313_1436	hypothetical protein	0.6	8.54E-06
Clo1313_1437	hypothetical protein	0.2	2.07E-02
Clo1313_1438	tRNA-i(6)A37 thiotransferase enzyme MiaB	0.2	2.43E-01
Clo1313_1439	protein of unknown function DUF534	-0.3	9.09E-03
Clo1313_1440	methyl-accepting chemotaxis sensory transducer with TarH sensor	-0.2	3.30E-01
Clo1313_1441	GCN5-related N-acetyltransferase	-0.2	6.21E-01
Clo1313_1442	HAD-superfamily hydrolase, subfamily IA, variant 3	-0.1	7.83E-01
Clo1313_1443	copper amine oxidase-like domain-containing protein	0.4	2.20E-03
Clo1313_1444	hypothetical protein	0.1	7.83E-01
Clo1313_1445	DNA mismatch repair protein MutS	0.0	8.79E-01
Clo1313_1446	DNA mismatch repair protein MutL	0.0	8.54E-01
Clo1313_1447	tRNA delta(2)-isopentenylpyrophosphate transferase	-0.1	6.51E-01
Clo1313_1448	RNA-binding protein Hfq	0.6	3.06E-11
Clo1313_1449	SOS-response transcriptional repressor, LexA	0.4	1.84E-03
Clo1313_1450	Peptidoglycan-binding lysin domain	0.8	4.18E-06
Clo1313_1451	helix-turn-helix protein YlxM/p13 family protein	-0.2	9.90E-02
Clo1313_1452	signal recognition particle subunit FFH/SRP54 (srp54)	-0.2	2.20E-02
Clo1313_1453	ribosomal protein S16	-0.3	1.13E-02
Clo1313_1454	RNA-binding protein (KH domain)	-0.1	6.54E-01
Clo1313_1455	16S rRNA processing protein RimM	-0.3	1.02E-02
Clo1313_1456	tRNA (guanine-N1)-methyltransferase	-0.2	3.19E-02
Clo1313_1457	LSU ribosomal protein L19P	-0.1	7.48E-01
Clo1313_1458	signal peptidase I	0.0	9.22E-01
Clo1313_1459	ribosome biogenesis GTP-binding protein YlqF	-0.2	1.79E-01
Clo1313_1460	hypothetical protein	-0.1	5.89E-01
Clo1313_1461	RNase HII (EC 3.1.26.4)	-0.1	4.39E-01
Clo1313_1462	hypothetical protein	0.1	3.88E-01
Clo1313_1463	type III secretion exporter	0.2	3.91E-01
Clo1313_1464	Uncharacterized protein family UPF0102	0.2	4.15E-01
Clo1313_1465	DNA repair protein RadC	0.0	8.89E-01
Clo1313_1466	dipeptidase. Metallo peptidase. MEROPS family M19	-0.1	8.38E-01
Clo1313_1467	aminotransferase class I and II	-1.5	3.50E-41
Clo1313_1468	hypothetical protein	0.0	9.08E-01
Clo1313_1469	hypothetical protein	-0.3	2.20E-01
Clo1313_1470	hypothetical protein	0.0	9.59E-01
Clo1313_1471	transcriptional regulator, XRE family with cupin sensor	-0.7	9.59E-05
Clo1313_1472	spermidine/putrescine ABC transporter ATPase subunit	-0.9	2.93E-11
Clo1313_1473	binding-protein-dependent transport systems inner membrane component	-1.0	8.60E-20
Clo1313_1474	binding-protein-dependent transport systems inner membrane component	-1.1	2.16E-20
Clo1313_1475	extracellular solute-binding protein family 1	-1.1	4.17E-35
Clo1313_1476	Peptidase S7 flavivirus helicase (NS3)	-1.0	3.50E-13
Clo1313_1477	glycoside hydrolase family 9	0.6	1.81E-07
Clo1313_1478	copper amine oxidase-like domain-containing protein	0.0	8.22E-01
Clo1313_1479	MATE efflux family protein	-0.1	5.57E-01
Clo1313_1480	hypothetical protein	0.1	7.66E-01

Clo1313_1481	adenylosuccinate lyase	0.0	9.45E-01
Clo1313_1482	transcriptional regulator, GntR family	0.4	1.05E-05
Clo1313_1483	pyrrolo-quinoline quinone	0.6	1.14E-08
Clo1313_1484	copper ion binding protein	0.3	1.44E-02
Clo1313_1485	hypothetical protein	0.0	9.49E-01
Clo1313_1486	4'-phosphopantetheinyl transferase	-0.1	7.19E-01
Clo1313_1487	cellulosome anchoring protein cohesin region	-0.2	1.16E-01
Clo1313_1488	cellulosome anchoring protein cohesin region	0.2	7.03E-02
Clo1313_1489	Peptidase M23	0.4	3.64E-04
Clo1313_1490	type IV pilus assembly PilZ	0.7	5.28E-05
Clo1313_1491	chorismate synthase (EC 4.2.3.5)	-0.1	2.58E-01
Clo1313_1492	shikimate kinase (EC 2.7.1.71)	0.2	3.21E-01
Clo1313_1493	hypothetical protein	0.2	2.03E-01
Clo1313_1494	Dockerin type 1	-0.7	5.76E-04
Clo1313_1495	integrase family protein	0.3	2.08E-01
Clo1313_1496	transcriptional attenuator, LytR family	0.6	3.65E-07
Clo1313_1497	peptidase M18 aminopeptidase I	0.1	3.10E-01
Clo1313_1498	response regulator receiver modulated metal dependent phosphohydrolase	0.5	9.99E-08
Clo1313_1499	histidinol phosphate phosphatase HisJ family	-0.4	1.05E-02
Clo1313_1500	tyrosyl-tRNA synthetase (EC 6.1.1.1)	0.4	2.78E-04
Clo1313_1501	tRNA (5-methylaminomethyl-2-thiouridylate)-methyltransferase (EC 2.1.1.61)	0.1	7.03E-01
Clo1313_1502	FeS cluster assembly scaffold protein NifU	-0.2	2.10E-01
Clo1313_1503	cysteine desulfurase NifS	-0.2	6.07E-02
Clo1313_1504	transcriptional regulator, BadM/Rrf2 family	-0.3	2.73E-01
Clo1313_1505	hypothetical protein	0.6	1.40E-01
Clo1313_1506	hypothetical protein	-0.1	8.99E-01
Clo1313_1507	MCP methyltransferase, CheR-type	0.5	7.92E-05
Clo1313_1508	nucleoside diphosphate kinase (EC 2.7.4.6)	0.4	3.18E-02
Clo1313_1509	adenosylmethionine decarboxylase proenzyme (EC 4.1.1.50)	0.5	2.54E-04
Clo1313_1510	4-hydroxy-3-methylbut-2-enyl diphosphate reductase (EC 1.17.1.2)	-0.2	1.26E-02
Clo1313_1511	1-acyl-sn-glycerol-3-phosphate acyltransferase (EC 2.3.1.51)	-0.1	4.04E-01
Clo1313_1512	cytidylate kinase (EC 2.7.4.14)	0.0	9.14E-01
Clo1313_1513	chorismate mutase (EC 5.4.99.5)	0.0	7.51E-01
Clo1313_1514	hypothetical protein	0.0	9.99E-01
Clo1313_1515	hypothetical protein	-0.1	7.05E-01
Clo1313_1516	HI0933 family protein	-0.1	7.51E-01
Clo1313_1517	Phosphoglycerate mutase	0.0	8.75E-01
Clo1313_1518	transcriptional regulator, RpiR family	0.0	8.96E-01
Clo1313_1519	hypothetical protein	0.1	8.47E-01
Clo1313_1520	hypothetical protein	0.6	2.74E-04
Clo1313_1521	hypothetical protein	0.7	1.57E-06
Clo1313_1522	hypothetical protein	0.6	1.47E-03
Clo1313_1523	Conserved carboxylase region	-0.2	5.60E-02
Clo1313_1524	biotin/lipoyl attachment domain-containing protein	-0.5	3.12E-04
Clo1313_1525	sodium pump decarboxylase gamma subunit	0.0	8.58E-01
Clo1313_1526	carboxyl transferase	-0.1	6.97E-01
Clo1313_1527	type IV pilus assembly PilZ	0.6	1.17E-03
Clo1313_1528	rRNA methylase	-0.1	5.19E-01
Clo1313_1529	agmatinase (EC 3.5.3.11)	0.0	9.66E-01
Clo1313_1530	spermidine synthase	0.1	3.82E-01
Clo1313_1531	pseudouridine synthase	0.2	3.41E-01
Clo1313_1532	sporulation protein YtfJ	0.5	5.34E-02
Clo1313_1533	hypothetical protein	0.4	3.06E-01
Clo1313_1534	RDD domain containing protein	0.2	4.79E-02
Clo1313_1535	chromosome segregation and condensation protein, ScpB	-0.2	2.22E-01
Clo1313_1536	condensin subunit ScpA	0.0	8.06E-01
Clo1313_1537	tryptophanyl-tRNA synthetase (EC 6.1.1.2)	0.1	3.12E-01
Clo1313_1538	peptidase M50	0.2	3.55E-01
Clo1313_1539	CBS domain containing protein	0.0	8.68E-01
Clo1313_1540	diaminopimelate decarboxylase	-0.5	5.36E-04
Clo1313_1541	hypothetical protein	0.3	4.67E-01
Clo1313_1542	IMP dehydrogenase	-0.2	8.85E-03
Clo1313_1543	Lysine exporter protein (LYSE/YGGA)	0.1	6.24E-01
Clo1313_1544	Serine-type D-Ala-D-Ala carboxypeptidase	-0.2	3.54E-01
Clo1313_1545	thymidine phosphorylase (EC 2.4.2.4)	-0.2	2.62E-01
Clo1313_1546	phosphopentomutase	-0.2	3.75E-01
Clo1313_1547	tyrosine recombinase XerD	1.0	7.37E-10
Clo1313_1548	stage II sporulation protein M	0.5	1.85E-01
Clo1313_1549	NUDIX hydrolase	0.2	3.09E-01
Clo1313_1550	Ribonuclease H	0.0	9.76E-01
Clo1313_1551	pyrroline-5-carboxylate reductase	0.0	9.93E-01
Clo1313_1552	hypothetical protein	0.2	6.02E-01
Clo1313_1553	spore germination protein	-0.2	6.20E-01
Clo1313_1554	hypothetical protein	NA	NA
Clo1313_1555	germination protein, Ger(x)C family	0.1	7.86E-01
Clo1313_1556	spore germination protein	0.3	4.50E-01

Clo1313_1557	GerA spore germination protein	0.4	3.41E-01
Clo1313_1558	protease FtsH subunit HflC	0.2	1.12E-01
Clo1313_1559	protease FtsH subunit HflK	0.0	9.68E-01
Clo1313_1560	3D domain-containing protein	0.4	3.20E-01
Clo1313_1561	hypothetical protein	0.3	4.13E-01
Clo1313_1562	Pseudo gene	-0.1	6.47E-01
Clo1313_1563	Ricin B lectin	0.0	9.27E-01
Clo1313_1564	glycoside hydrolase family 81	-0.1	6.00E-01
Clo1313_1565	hypothetical protein	0.1	7.86E-01
Clo1313_1566	hypothetical protein	0.1	8.54E-01
Clo1313_1567	hypothetical protein	0.4	6.19E-03
Clo1313_1568	hypothetical protein	0.0	9.56E-01
Clo1313_1569	type IV pilus assembly protein PilM	0.1	1.98E-01
Clo1313_1570	cysteine desulfurase family protein	-0.7	4.87E-09
Clo1313_1571	iron-only hydrogenase maturation protein HydG	-1.2	1.47E-35
Clo1313_1572	hypothetical protein	-1.2	4.73E-17
Clo1313_1573	4-oxalocrotonate tautomerase	0.4	2.59E-01
Clo1313_1574	hypothetical protein	0.3	4.05E-01
Clo1313_1575	hypothetical protein	-0.1	8.89E-01
Clo1313_1576	hypothetical protein	0.0	9.65E-01
Clo1313_1577	hypothetical protein	0.3	5.22E-01
Clo1313_1578	glutamyl-tRNA synthetase	0.3	5.47E-03
Clo1313_1579	anaerobic ribonucleoside-triphosphate reductase activating protein	0.6	5.34E-07
Clo1313_1580	ribonucleoside-triphosphate reductase class III catalytic subunit (EC 1.17.4.2)	0.5	2.50E-07
Clo1313_1581	methionine synthase (B12-dependent) (EC 2.1.1.13)	0.1	4.10E-01
Clo1313_1582	Vitamin B12 dependent methionine synthase activation region	0.3	8.96E-02
Clo1313_1583	uncharacterized membrane protein	-0.2	6.02E-01
Clo1313_1584	hypothetical protein	-0.4	3.54E-01
Clo1313_1585	Coat F domain protein	0.9	6.76E-05
Clo1313_1586	hypothetical protein	-0.1	8.17E-01
Clo1313_1587	Dockerin type 1	-0.2	3.42E-01
Clo1313_1588	glycoside hydrolase family 13 domain protein	0.2	7.39E-02
Clo1313_1589	FHA domain containing protein	0.4	6.31E-06
Clo1313_1590	hypothetical protein	0.4	2.85E-02
Clo1313_1591	hypothetical protein	0.4	2.19E-01
Clo1313_1592	protein serine/threonine phosphatase	0.7	2.03E-10
Clo1313_1593	serine/threonine protein kinase	0.5	9.82E-07
Clo1313_1594	hypothetical protein	0.0	9.96E-01
Clo1313_1595	hypothetical protein	0.0	8.45E-01
Clo1313_1596	hypothetical protein	0.1	7.26E-01
Clo1313_1597	hypothetical protein	-0.2	9.32E-02
Clo1313_1598	hypothetical protein	0.1	5.20E-01
Clo1313_1599	type II secretion system protein E	-0.1	5.93E-01
Clo1313_1600	hypothetical protein	0.2	4.06E-01
Clo1313_1601	hypothetical protein	-0.1	4.24E-01
Clo1313_1602	hypothetical protein	0.2	3.07E-01
Clo1313_1603	glycoside hydrolase family 9	0.5	7.52E-10
Clo1313_1604	glycoside hydrolase family 9	0.1	3.73E-01
Clo1313_1605	transcriptional regulator, TraR/DksA family	-0.3	9.61E-02
Clo1313_1606	methylthioadenosine phosphorylase (EC 2.4.2.28)	0.3	7.21E-02
Clo1313_1607	methylthioribose-1-phosphate isomerase (EC 5.3.1.23)	-0.1	3.38E-01
Clo1313_1608	iron (metal) dependent repressor, DtxR family	0.4	1.95E-03
Clo1313_1609	FeoA family protein	-1.3	3.20E-10
Clo1313_1610	ferrous iron transport protein B	-1.6	1.01E-22
Clo1313_1611	hypothetical protein	-0.4	2.91E-01
Clo1313_1612	AAA ATPase central domain protein	-0.2	1.44E-02
Clo1313_1613	amino acid-binding ACT domain protein	0.1	6.33E-01
Clo1313_1614	phenylacetate-CoA ligase (EC 6.2.1.30)	-0.2	1.58E-01
Clo1313_1615	indolepyruvate ferredoxin oxidoreductase, beta subunit	0.0	7.92E-01
Clo1313_1616	indolepyruvate ferredoxin oxidoreductase, alpha subunit	0.1	5.10E-01
Clo1313_1617	SSS sodium solute transporter superfamily	0.2	5.89E-03
Clo1313_1618	RNA-metabolising metallo-beta-lactamase	0.3	5.73E-03
Clo1313_1619	histidinol phosphate aminotransferase apoenzyme (EC 2.6.1.9)	0.9	3.63E-07
Clo1313_1620	peptidase M42 family protein	0.7	6.89E-06
Clo1313_1621	Cellulase	0.3	1.29E-01
Clo1313_1622	peptidase M42 family protein	0.1	7.27E-01
Clo1313_1623	DNA internalization-related competence protein ComEC/Rec2	-0.1	1.95E-01
Clo1313_1624	NLP/P60 protein	-0.2	1.37E-01
Clo1313_1625	hypothetical protein	-0.3	2.11E-01
Clo1313_1626	GCN5-related N-acetyltransferase	-0.4	4.49E-03
Clo1313_1627	hydroxymethylpyrimidine synthase	-0.3	3.90E-03
Clo1313_1628	thiamine-phosphate pyrophosphorylase	-0.4	7.97E-03
Clo1313_1629	thiamine biosynthesis protein ThiF	-0.3	7.11E-02
Clo1313_1630	tyrosine lyase ThiH	-0.4	5.52E-02
Clo1313_1631	thiazole-phosphate synthase	-0.3	3.39E-01
Clo1313_1632	thiamine biosynthesis protein ThiS	-0.1	6.92E-01

Clo1313_1633	ribosome biogenesis GTP-binding protein YsxC	0.1	7.28E-01
Clo1313_1634	hypothetical protein	0.3	5.10E-01
Clo1313_1635	hypothetical protein	0.2	4.10E-01
Clo1313_1636	hypothetical protein	0.4	4.64E-02
Clo1313_1637	copper amine oxidase-like domain-containing protein	0.5	6.06E-06
Clo1313_1638	Methyltransferase type 11	-0.4	3.13E-01
Clo1313_1639	Pseudo gene	0.3	2.60E-01
Clo1313_1640	Pseudo gene	0.1	6.92E-01
Clo1313_1641	Pseudo gene	NA	NA
Clo1313_1642	hypothetical protein	-0.2	6.66E-01
Clo1313_1643	Resolvase domain protein	-0.1	6.03E-01
Clo1313_1644	hypothetical protein	0.0	9.51E-01
Clo1313_1645	DEAD/DEAH box helicase domain protein	-0.3	5.15E-02
Clo1313_1646	Pseudo gene	-0.2	2.94E-01
Clo1313_1647	hypothetical protein	-0.3	2.59E-02
Clo1313_1648	Resolvase domain protein	0.0	8.86E-01
Clo1313_1649	hypothetical protein	0.0	8.33E-01
Clo1313_1650	DNA binding domain protein, excisionase family	NA	NA
Clo1313_1651	transposase IS3/IS911 family protein	-0.3	1.09E-01
Clo1313_1652	hypothetical protein	0.3	4.78E-01
Clo1313_1653	protein of unknown function DUF503	0.5	5.09E-03
Clo1313_1654	response regulator receiver protein	-0.1	7.42E-01
Clo1313_1655	PAS/PAC sensor signal transduction histidine kinase	-0.1	4.46E-01
Clo1313_1656	Fibronectin-binding A domain protein	0.3	2.62E-02
Clo1313_1657	aminotransferase class I and II	0.4	3.11E-04
Clo1313_1658	nitroreductase	-0.1	7.27E-01
Clo1313_1659	endoglucanase Cel9R	0.1	2.51E-01
Clo1313_1660	thiamine diphosphokinase	0.1	1.67E-01
Clo1313_1661	ribulose-5-phosphate 3-epimerase (EC 5.1.3.1)	0.2	1.45E-01
Clo1313_1662	ribosome small subunit-dependent GTPase A	0.2	1.73E-01
Clo1313_1663	serine/threonine protein kinase with PASTA sensor(s)	0.4	6.58E-06
Clo1313_1664	protein serine/threonine phosphatase	0.2	9.82E-03
Clo1313_1665	23S rRNA m(2)A-2503 methyltransferase (EC 2.1.1.192)	-0.1	2.43E-01
Clo1313_1666	NusB antitermination factor	0.0	7.04E-01
Clo1313_1667	peptidase membrane zinc metallopeptidase	0.2	1.91E-01
Clo1313_1668	protein of unknown function DUF116	0.2	2.62E-01
Clo1313_1669	methionyl-tRNA formyltransferase (EC 2.1.2.9)	0.1	6.95E-01
Clo1313_1670	peptide deformylase (EC 3.5.1.88)	0.2	2.43E-01
Clo1313_1671	replication restart DNA helicase PriA	0.0	8.92E-01
Clo1313_1672	hypothetical protein	0.1	4.07E-01
Clo1313_1673	Polyprenyl synthetase	-0.2	8.63E-02
Clo1313_1674	Heptaprenyl diphosphate synthase component I	0.1	7.53E-01
Clo1313_1675	protein of unknown function DUF1312	-0.2	5.97E-01
Clo1313_1676	ApbE family lipoprotein	-0.3	2.28E-02
Clo1313_1677	FAD-dependent pyridine nucleotide-disulfide oxidoreductase	0.0	8.13E-01
Clo1313_1678	single-strand binding protein/Primosomal replication protein n	0.8	1.22E-04
Clo1313_1679	Mur ligase middle domain protein	0.7	4.64E-04
Clo1313_1680	polysaccharide deacetylase	1.2	2.33E-04
Clo1313_1681	asparagine synthase (glutamine-hydrolyzing)	0.3	3.68E-01
Clo1313_1682	PpiC-type peptidyl-prolyl cis-trans isomerase	-0.3	1.00E-03
Clo1313_1683	phosphoribosylformylglycinamidine synthase (EC 6.3.5.3)	-0.7	3.29E-15
Clo1313_1684	transcriptional regulator, LysR family	0.0	9.37E-01
Clo1313_1685	transcriptional regulator, XRE family with cupin sensor	0.4	2.96E-02
Clo1313_1686	AMP-dependent synthetase and ligase	0.1	1.81E-01
Clo1313_1687	Heat shock protein Hsp90-like protein	0.1	7.16E-01
Clo1313_1688	ABC-3 protein	0.0	8.89E-01
Clo1313_1689	ABC transporter related protein	0.2	1.72E-01
Clo1313_1690	periplasmic solute binding protein	0.2	2.47E-01
Clo1313_1691	ferric uptake regulator, Fur family	0.7	1.23E-03
Clo1313_1692	peptidase A24A prepilin type IV	0.0	9.06E-01
Clo1313_1693	protein serine/threonine phosphatase	-0.1	7.37E-01
Clo1313_1694	endoglucanase Cel9W	0.0	7.18E-01
Clo1313_1695	protein of unknown function DUF45	0.1	6.99E-01
Clo1313_1696	peptidase M24	-0.1	6.70E-01
Clo1313_1697	protein of unknown function DUF214	0.0	9.19E-01
Clo1313_1698	ABC transporter related protein	-0.1	6.73E-01
Clo1313_1699	integral membrane sensor signal transduction histidine kinase	-0.2	3.28E-01
Clo1313_1700	two component transcriptional regulator, winged helix family	-0.1	8.24E-01
Clo1313_1701	glycoside hydrolase family 5	-0.3	3.68E-06
Clo1313_1702	hypothetical protein	0.3	1.87E-01
Clo1313_1703	ABC-type bacteriocin transporter	0.3	1.14E-01
Clo1313_1704	Radical SAM domain protein	0.6	3.13E-03
Clo1313_1705	Haloacid dehalogenase domain protein hydrolase	0.4	2.92E-01
Clo1313_1706	hypothetical protein	0.3	1.50E-01
Clo1313_1707	hypothetical protein	0.4	3.64E-02
Clo1313_1708	Peptidoglycan-binding lysin domain	0.0	9.46E-01
Clo1313_1709	hypothetical protein	0.1	7.69E-01
Clo1313_1710	transposase IS200-family protein	NA	NA

Clo1313_1711	histidine kinase	1.1	5.64E-22
Clo1313_1712	sodium ion-translocating decarboxylase, beta subunit	0.5	1.52E-09
Clo1313_1713	sodium pump decarboxylase gamma subunit	0.9	1.03E-03
Clo1313_1714	hypothetical protein	1.1	1.22E-07
Clo1313_1715	Exonuclease RNase T and DNA polymerase III	0.4	3.29E-02
Clo1313_1716	pyruvate formate-lyase activating enzyme	0.2	1.79E-01
Clo1313_1717	formate acetyltransferase	-0.2	1.60E-01
Clo1313_1718	Capsule synthesis protein, CapA	0.0	8.06E-01
Clo1313_1719	2'-5' RNA ligase	-0.1	8.54E-01
Clo1313_1720	hypothetical protein	-0.2	6.84E-01
Clo1313_1721	aminodeoxychorismate lyase	0.1	3.14E-01
Clo1313_1722	hypothetical protein	0.5	1.14E-04
Clo1313_1723	hypothetical protein	0.4	2.13E-02
Clo1313_1724	protein of unknown function DUF342	0.3	2.17E-02
Clo1313_1725	RNA polymerase, sigma 28 subunit, SigD/FliA/WhiG	0.6	3.18E-05
Clo1313_1726	hypothetical protein	0.5	2.66E-03
Clo1313_1727	CheD	0.6	8.03E-04
Clo1313_1728	CheC, inhibitor of MCP methylation	0.5	2.29E-02
Clo1313_1729	CheW protein	0.6	2.40E-04
Clo1313_1730	CheA signal transduction histidine kinase	0.7	8.26E-13
Clo1313_1731	response regulator receiver modulated CheB methylesterase	0.8	2.74E-10
Clo1313_1732	type IV pilus assembly PilZ	0.8	4.58E-05
Clo1313_1733	cobyrinic acid ac-diamide synthase	0.6	4.83E-05
Clo1313_1734	flagellar biosynthetic protein FlhF	0.4	5.82E-04
Clo1313_1735	flagellar biosynthesis protein FlhA	0.5	3.02E-08
Clo1313_1736	flagellar biosynthetic protein FlhB	0.2	6.30E-03
Clo1313_1737	flagellar biosynthetic protein FliR	0.4	2.71E-04
Clo1313_1738	flagellar biosynthetic protein FliQ	0.8	6.29E-04
Clo1313_1739	flagellar biosynthetic protein FliP	1.2	7.10E-14
Clo1313_1740	hypothetical protein	0.8	3.68E-12
Clo1313_1741	response regulator receiver protein	0.4	1.75E-02
Clo1313_1742	CheC, inhibitor of MCP methylation / FliN fusion protein	0.4	1.37E-03
Clo1313_1743	flagellar motor switch protein FliM	0.1	3.29E-01
Clo1313_1744	flagellar basal body-associated protein FliL	0.5	1.44E-04
Clo1313_1745	flagellar FlbD family protein	0.4	2.62E-01
Clo1313_1746	flagellar hook-basal body protein	0.1	4.61E-01
Clo1313_1747	flagellar operon protein	0.4	8.18E-02
Clo1313_1748	flagellar hook capping protein	0.5	2.14E-04
Clo1313_1749	Flagellar hook-length control protein-like, C-terminal domain	0.5	8.55E-09
Clo1313_1750	hypothetical protein	0.5	9.14E-04
Clo1313_1751	flagellar export protein FliJ	0.5	4.97E-02
Clo1313_1752	type III secretion system ATPase, FliI/YscN (EC 3.6.3.15)	0.5	1.69E-03
Clo1313_1753	Flagellar assembly protein FliH/Type III secretion system HrpE	0.4	1.56E-02
Clo1313_1754	flagellar motor switch protein FliG	0.5	5.17E-05
Clo1313_1755	flagellar M-ring protein FliF	0.8	8.40E-17
Clo1313_1756	flagellar hook-basal body complex subunit FliE	0.7	1.79E-04
Clo1313_1757	flagellar basal-body rod protein FlgC	0.7	5.20E-10
Clo1313_1758	flagellar basal-body rod protein FlgB	0.6	3.19E-09
Clo1313_1759	gid protein	1.0	3.10E-25
Clo1313_1760	DNA topoisomerase I	0.6	3.34E-10
Clo1313_1761	DNA protecting protein DprA	0.4	5.83E-05
Clo1313_1762	exodeoxyribonuclease III Xth	0.3	2.95E-03
Clo1313_1763	protein of unknown function DUF307	0.4	7.46E-02
Clo1313_1764	protein of unknown function UPF0047	0.4	1.20E-01
Clo1313_1765	Mg chelatase, subunit ChlI	0.5	3.85E-04
Clo1313_1766	hypothetical protein	0.5	1.86E-01
Clo1313_1767	hypothetical protein	-0.1	8.00E-01
Clo1313_1768	cellulosome anchoring protein cohesin region	0.5	1.66E-04
Clo1313_1769	protein of unknown function DUF115	0.2	1.31E-01
Clo1313_1770	ATP-cone domain protein	0.6	9.95E-04
Clo1313_1771	sporulation protein, YlmC/YmxH family	0.0	9.47E-01
Clo1313_1772	RNA polymerase, sigma subunit, RpsG/SigG	0.3	3.32E-01
Clo1313_1773	RNA polymerase, sigma 29 subunit, SigE	-0.2	4.23E-01
Clo1313_1774	sigma-E processing peptidase SpoIIGA	0.3	4.94E-01
Clo1313_1775	cell division protein FtsZ	0.3	3.99E-02
Clo1313_1776	cell division protein FtsA	0.5	9.39E-07
Clo1313_1777	protein of unknown function DUF1290	0.4	2.50E-02
Clo1313_1778	cell division protein FtsQ	0.3	1.92E-03
Clo1313_1779	Pseudo gene	0.3	1.69E-01
Clo1313_1780	domain of unknown function DUF1727	0.2	2.00E-01
Clo1313_1781	CobB/CobQ domain protein glutamine amidotransferase	-0.1	6.01E-01
Clo1313_1782	transposase mutator type	0.0	8.62E-01
Clo1313_1783	Dockerin type 1	0.3	4.76E-01
Clo1313_1784	hypothetical protein	0.3	7.97E-03
Clo1313_1785	Tetratricopeptide TPR_1 repeat-containing protein	0.0	7.43E-01
Clo1313_1786	Dockerin type 1	0.2	8.55E-02
Clo1313_1787	ATPase	-0.3	3.79E-01
Clo1313_1788	endoglucanase Cel9P	0.3	1.42E-03

Clo1313_1789	hypothetical protein	0.1	7.96E-01
Clo1313_1790	hypothetical protein	0.1	5.40E-01
Clo1313_1791	hydrogenase, Fe-only	-0.1	2.67E-01
Clo1313_1792	NADH dehydrogenase (quinone)	0.0	8.79E-01
Clo1313_1793	NADH dehydrogenase (ubiquinone) 24 kDa subunit	0.2	3.66E-01
Clo1313_1794	Stage II sporulation protein E	-0.5	4.41E-05
Clo1313_1795	putative PAS/PAC sensor protein	0.0	7.97E-01
Clo1313_1796	hypothetical protein	0.4	8.15E-03
Clo1313_1797	aminoglycoside phosphotransferase	0.1	3.72E-01
Clo1313_1798	acetaldehyde dehydrogenase (EC 1.2.1.10)/alcohol dehydrogenase AdhE (EC 1.1.1.1)	0.1	5.34E-01
Clo1313_1799	CoA-binding domain protein	0.3	3.31E-02
Clo1313_1800	dipicolinic acid synthetase, B subunit	-0.8	3.90E-03
Clo1313_1801	dipicolinate synthase subunit A	-1.1	1.44E-09
Clo1313_1802	peptidase M16 domain protein	-0.5	4.34E-06
Clo1313_1803	polyribonucleotide nucleotidyltransferase	-0.3	4.02E-05
Clo1313_1804	SSU ribosomal protein S15P	0.2	3.30E-01
Clo1313_1805	hypothetical protein	-0.1	6.47E-01
Clo1313_1806	spore coat protein CotJB	NA	NA
Clo1313_1807	manganese containing catalase	0.2	6.65E-01
Clo1313_1808	glycoside hydrolase family 9	0.8	1.63E-21
Clo1313_1809	cellulose 1,4-beta-cellobiosidase (EC 3.2.1.91)	-0.1	5.20E-01
Clo1313_1810	hypothetical protein	0.3	4.17E-02
Clo1313_1811	protein of unknown function DUF711	0.0	7.15E-01
Clo1313_1812	ACT domain-containing protein	-0.3	2.09E-01
Clo1313_1813	periplasmic sensor diguanylate cyclase/phosphodiesterase	-0.1	6.10E-01
Clo1313_1814	radical SAM domain-containing protein	0.5	3.24E-03
Clo1313_1815	hypothetical protein	0.2	2.23E-01
Clo1313_1816	glycoside hydrolase family 5	-0.3	4.05E-03
Clo1313_1817	type 3a cellulose-binding domain protein	-0.2	1.04E-01
Clo1313_1818	RNA polymerase, sigma 28 subunit, SigI	0.1	4.49E-01
Clo1313_1819	copper amine oxidase-like domain-containing protein	0.1	1.76E-01
Clo1313_1820	methyl-accepting chemotaxis sensory transducer	0.6	1.63E-09
Clo1313_1821	hypothetical protein	0.3	3.17E-02
Clo1313_1822	protein of unknown function DUF477	0.3	3.74E-02
Clo1313_1823	protein of unknown function DUF74	0.5	7.82E-03
Clo1313_1824	ABC transporter transmembrane region	-0.3	1.10E-01
Clo1313_1825	ABC transporter related protein	0.3	1.44E-01
Clo1313_1826	RbsD or FucU transport	0.8	5.30E-05
Clo1313_1827	iron-containing alcohol dehydrogenase	0.1	3.97E-01
Clo1313_1828	periplasmic binding protein/LacI transcriptional regulator	0.1	6.02E-01
Clo1313_1829	inner-membrane translocator	0.0	9.29E-01
Clo1313_1830	ABC transporter related protein	0.0	9.44E-01
Clo1313_1831	ROK family protein	0.7	3.73E-07
Clo1313_1832	PfkB domain protein	-0.1	5.55E-01
Clo1313_1833	Alcohol dehydrogenase GroES domain protein	0.1	2.07E-01
Clo1313_1834	hypothetical protein	0.8	2.34E-02
Clo1313_1835	hypothetical protein	0.0	8.92E-01
Clo1313_1836	Pseudo gene	NA	NA
Clo1313_1837	Pseudo gene	0.0	9.83E-01
Clo1313_1838	ABC transporter related protein	0.2	5.62E-01
Clo1313_1839	Pseudo gene	0.3	4.54E-01
Clo1313_1840	hypothetical protein	0.3	1.12E-01
Clo1313_1841	hypothetical protein	0.0	9.41E-01
Clo1313_1842	hypothetical protein	-0.6	8.09E-02
Clo1313_1843	hypothetical protein	0.1	8.78E-01
Clo1313_1844	peptidase M56 BlaR1	-0.4	2.88E-01
Clo1313_1845	transcriptional repressor, CopY family	-0.1	7.95E-01
Clo1313_1846	GMP synthase, large subunit	-0.4	2.70E-06
Clo1313_1847	glutamate dehydrogenase (NADP) (EC 1.4.1.4)	-1.0	1.44E-18
Clo1313_1848	oxidoreductase FAD/NAD(P)-binding domain protein	0.1	6.07E-01
Clo1313_1849	sulfide dehydrogenase (flavoprotein) subunit SudA (EC 1.8.1.-)	0.1	6.11E-01
Clo1313_1850	transposase mutator type	-0.1	4.23E-01
Clo1313_1851	conserved hypothetical protein	0.1	3.66E-01
Clo1313_1852	protein of unknown function DUF111	0.2	5.59E-02
Clo1313_1853	1-(5-phosphoribosyl)-5-amino-4-imidazole- carboxylate (AIR) carboxylase	0.2	2.20E-01
Clo1313_1854	Domain of unknown function DUF2520	0.3	3.90E-02
Clo1313_1855	copper amine oxidase-like domain-containing protein	1.3	1.16E-39
Clo1313_1856	Peptide chain release factor 2	0.2	5.61E-03
Clo1313_1857	helix-turn-helix domain protein	0.1	7.54E-01
Clo1313_1858	aminotransferase class I and II	0.4	5.80E-04
Clo1313_1859	transcriptional regulator, AsnC family	0.4	2.28E-05
Clo1313_1860	hypothetical protein	0.2	4.71E-01
Clo1313_1861	hypothetical protein	-0.1	3.35E-01
Clo1313_1862	thioredoxin	0.1	6.17E-01
Clo1313_1863	Pseudo gene	-0.2	4.80E-01
Clo1313_1864	transposase mutator type	NA	NA

Clo1313_1865	transposase IS3/IS911 family protein	NA	NA
Clo1313_1866	hypothetical protein	0.4	2.30E-01
Clo1313_1867	maltodextrin phosphorylase	0.2	1.18E-01
Clo1313_1868	transposase mutator type	-0.1	7.66E-01
Clo1313_1869	hypothetical protein	0.1	6.61E-01
Clo1313_1870	CoA-substrate-specific enzyme activase	0.0	9.38E-01
Clo1313_1871	hypothetical protein	0.2	6.02E-01
Clo1313_1872	Protein of unknown function DUF2229, CoA enzyme activase	0.5	1.05E-01
Clo1313_1873	PpiC-type peptidyl-prolyl cis-trans isomerase	-0.1	7.14E-01
Clo1313_1874	signal peptidase I (EC:3.4.21.89). Serine peptidase. MEROPS family S26A	-0.3	3.48E-03
Clo1313_1875	fructose-bisphosphate aldolase (EC 4.1.2.13)	-0.3	4.43E-03
Clo1313_1876	phosphofructokinase	-0.7	2.42E-12
Clo1313_1877	hypothetical protein	0.1	7.15E-01
Clo1313_1878	L-lactate dehydrogenase	-0.4	5.49E-06
Clo1313_1879	malic protein NAD-binding protein	-0.4	2.32E-07
Clo1313_1880	flavin reductase domain protein FMN-binding protein	0.0	9.65E-01
Clo1313_1881	NAD(P)-dependent iron-only hydrogenase catalytic subunit	-0.6	8.43E-08
Clo1313_1882	NAD(P)-dependent iron-only hydrogenase diaphorase component flavoprotein	-0.6	2.29E-10
Clo1313_1883	NAD(P)-dependent iron-only hydrogenase iron-sulfur protein	-0.4	2.83E-04
Clo1313_1884	ATP-binding region ATPase domain protein	-0.4	2.43E-05
Clo1313_1885	NAD(P)-dependent iron-only hydrogenase diaphorase component iron-sulfur protein	-0.3	3.24E-03
Clo1313_1886	PHP domain protein	0.1	4.72E-01
Clo1313_1887	DRTGG domain protein	-0.1	4.75E-01
Clo1313_1888	Fe-S cluster domain protein	0.2	1.73E-01
Clo1313_1889	putative anti-sigma regulatory factor, serine/threonine protein kinase	0.2	4.46E-01
Clo1313_1890	hypothetical protein	0.1	5.58E-01
Clo1313_1891	TGS domain-containing protein	0.2	9.33E-02
Clo1313_1892	hypothetical protein	0.1	7.56E-01
Clo1313_1893	Pseudo gene	0.0	8.70E-01
Clo1313_1894	Domain of unknown function DUF1848	-0.1	3.54E-01
Clo1313_1895	hypothetical protein	-0.1	7.83E-01
Clo1313_1896	bacterial peptide chain release factor 3 (bRF-3)	-0.4	2.37E-06
Clo1313_1897	conserved repeat domain protein	0.2	3.27E-01
Clo1313_1898	transposase IS200-family protein	NA	NA
Clo1313_1899	hypothetical protein	0.2	4.72E-01
Clo1313_1900	hypothetical protein	0.5	8.90E-02
Clo1313_1901	NAD+ synthetase	0.0	9.63E-01
Clo1313_1902	valyl-tRNA synthetase (EC 6.1.1.9)	0.0	8.26E-01
Clo1313_1903	hypothetical protein	0.4	2.10E-01
Clo1313_1904	glycoside hydrolase family 3 domain protein	0.1	8.09E-01
Clo1313_1905	Tetratricopeptide TPR_1 repeat-containing protein	-0.1	5.34E-01
Clo1313_1906	hypothetical protein	-0.1	5.26E-01
Clo1313_1907	class II aldolase/adducin family protein	0.0	9.66E-01
Clo1313_1908	ErfK/YbiS/YcfS/YnhG family protein	0.0	8.92E-01
Clo1313_1909	hypothetical protein	-0.4	4.05E-03
Clo1313_1910	PA14 domain protein	-0.1	7.77E-01
Clo1313_1911	RNA polymerase sigma-I factor	0.2	6.85E-01
Clo1313_1912	Glycosyltransferase 28 domain	0.2	4.54E-01
Clo1313_1913	hypothetical protein	0.3	1.35E-01
Clo1313_1914	ATPase AAA-2 domain protein	0.4	3.61E-06
Clo1313_1915	transposase, IS4 family	0.0	9.13E-01
Clo1313_1916	excinuclease ABC, A subunit	0.2	1.71E-01
Clo1313_1917	3D domain-containing protein	0.3	9.60E-03
Clo1313_1918	Excinuclease ABC subunit B	0.3	4.65E-03
Clo1313_1919	Pseudo gene	0.2	1.28E-01
Clo1313_1920	hypothetical protein	0.3	9.24E-03
Clo1313_1921	DNA topoisomerase (ATP-hydrolyzing)	0.1	2.73E-01
Clo1313_1922	DNA gyrase subunit B domain protein	0.2	1.65E-01
Clo1313_1923	hypothetical protein	0.6	1.32E-02
Clo1313_1924	hypothetical protein	0.9	1.19E-02
Clo1313_1925	hypothetical protein	0.1	7.62E-01
Clo1313_1926	protein of unknown function DUF1212	0.5	1.88E-01
Clo1313_1927	spore coat assembly protein SafA	0.6	6.45E-02
Clo1313_1928	MATE efflux family protein	0.0	9.34E-01
Clo1313_1929	methyl-accepting chemotaxis sensory transducer	0.2	2.78E-01
Clo1313_1930	hypothetical protein	0.3	1.58E-01
Clo1313_1931	phosphoserine aminotransferase apoenzyme (EC 2.6.1.52)	-0.1	7.09E-01
Clo1313_1932	Pseudo gene	-0.2	3.05E-01
Clo1313_1933	hypothetical protein	0.1	7.34E-01
Clo1313_1934	Pseudo gene	-0.5	1.08E-03
Clo1313_1935	Integrase catalytic region	NA	NA
Clo1313_1936	hypothetical protein	0.3	2.92E-01
Clo1313_1937	hypothetical protein	-0.3	1.04E-01
Clo1313_1938	homoserine dehydrogenase (EC 1.1.1.3)	0.2	2.33E-01

Clo1313_1939	helicase c2	-0.3	7.55E-04
Clo1313_1940	MCP methyltransferase, CheR-type	-0.2	1.21E-01
Clo1313_1941	Pseudo gene	-0.9	7.02E-12
Clo1313_1942	response regulator receiver sensor signal transduction histidine kinase	-0.7	5.58E-04
Clo1313_1943	hypothetical protein	NA	NA
Clo1313_1944	isocitrate dehydrogenase (NADP) (EC 1.1.1.42)	-0.7	5.93E-18
Clo1313_1945	protein of unknown function DUF815	-0.1	2.94E-01
Clo1313_1946	aldo/keto reductase	0.0	8.98E-01
Clo1313_1947	glycogen/starch synthase, ADP-glucose type	0.9	1.57E-08
Clo1313_1948	Coat F domain protein	0.2	5.40E-01
Clo1313_1949	hypothetical protein	0.1	8.27E-01
Clo1313_1950	ribosomal large subunit pseudouridine synthase D (EC 5.4.99.-)	0.1	7.35E-01
Clo1313_1951	hypothetical protein	0.3	6.25E-02
Clo1313_1952	hypothetical protein	0.1	8.04E-01
Clo1313_1953	D-isomer specific 2-hydroxyacid dehydrogenase NAD-binding protein	0.2	7.31E-02
Clo1313_1954	cellobiose phosphorylase (EC 2.4.1.20)	0.0	9.93E-01
Clo1313_1955	endoglucanase Cel9M	0.6	1.75E-04
Clo1313_1956	diguanylate cyclase and metal dependent phosphohydrolase	0.2	6.31E-02
Clo1313_1957	Serine-type D-Ala-D-Ala carboxypeptidase	0.5	7.19E-03
Clo1313_1958	type 3a cellulose-binding domain protein	0.1	2.60E-01
Clo1313_1959	glycoside hydrolase family 18	0.3	1.92E-02
Clo1313_1960	glycoside hydrolase family 8	0.0	9.09E-01
Clo1313_1961	RNA polymerase sigma-I factor	0.4	1.44E-02
Clo1313_1962	type 3a cellulose-binding domain protein	0.4	3.44E-02
Clo1313_1963	methyl-accepting chemotaxis sensory transducer	0.5	3.70E-08
Clo1313_1964	Alanine--glyoxylate transaminase	0.4	3.11E-10
Clo1313_1965	hypothetical protein	0.0	9.67E-01
Clo1313_1966	hypothetical protein	-0.1	8.24E-01
Clo1313_1967	glutamate-5-semialdehyde dehydrogenase (EC 1.2.1.41)	0.2	5.03E-03
Clo1313_1968	HAD-superfamily hydrolase, subfamily IA, variant 1	0.0	9.78E-01
Clo1313_1969	peptidase S1 and S6 chymotrypsin/Hap	0.7	5.24E-09
Clo1313_1970	Rhomboid family protein	0.0	9.38E-01
Clo1313_1971	Dockerin type 1	0.0	9.30E-01
Clo1313_1972	twitching motility protein	0.6	1.71E-02
Clo1313_1973	histidine kinase	0.8	6.36E-08
Clo1313_1974	hypothetical protein	0.1	8.77E-01
Clo1313_1975	protein of unknown function DUF58	-0.1	6.58E-01
Clo1313_1976	ATPase associated with various cellular activities AAA_3	0.1	7.14E-01
Clo1313_1977	hypothetical protein	-0.1	7.05E-01
Clo1313_1978	transglutaminase domain-containing protein	-0.3	7.73E-03
Clo1313_1979	protein of unknown function UPF0027	-0.6	2.49E-07
Clo1313_1980	peptidase C11 clostripain	-0.4	3.24E-06
Clo1313_1981	transposase, IS4 family	-0.1	7.15E-01
Clo1313_1982	DNA mismatch repair protein MutS domain protein	-0.1	4.68E-01
Clo1313_1983	Carbohydrate binding family 6	0.3	1.66E-01
Clo1313_1984	transcriptional regulator, ArsR family	-0.1	6.16E-01
Clo1313_1985	cadmium-translocating P-type ATPase	0.2	2.70E-01
Clo1313_1986	copper amine oxidase-like domain-containing protein	-0.5	2.30E-05
Clo1313_1987	hypothetical protein	-0.1	4.85E-01
Clo1313_1988	hypothetical protein	0.7	3.10E-07
Clo1313_1989	VTC domain	0.8	1.55E-09
Clo1313_1990	Spore coat protein CotH	0.6	2.91E-02
Clo1313_1991	Pseudo gene	0.2	1.73E-01
Clo1313_1992	aldo/keto reductase	-0.1	4.47E-01
Clo1313_1993	glutaredoxin-like protein, YruB-family	0.0	9.30E-01
Clo1313_1994	AMP-dependent synthetase and ligase	0.3	1.36E-03
Clo1313_1995	GCN5-related N-acetyltransferase	-0.6	1.55E-07
Clo1313_1996	hypothetical protein	-0.3	1.35E-02
Clo1313_1997	Radical SAM domain protein	-0.1	5.80E-01
Clo1313_1998	hypothetical protein	-0.2	5.40E-02
Clo1313_1999	NAD-dependent epimerase/dehydratase	-0.1	8.06E-01
Clo1313_2000	hypothetical protein	-0.1	6.74E-01
Clo1313_2001	hypothetical protein	-0.7	5.36E-06
Clo1313_2002	hypothetical protein	0.3	5.29E-02
Clo1313_2003	Pseudo gene	-0.3	5.64E-02
Clo1313_2004	UvrD/REP helicase	-0.3	9.60E-03
Clo1313_2005	SMC domain protein	-0.2	6.87E-02
Clo1313_2006	ADP-ribosylation/Crystallin J1	0.1	4.25E-01
Clo1313_2007	Pseudo gene	-0.8	8.44E-03
Clo1313_2008	transposase mutator type	NA	NA
Clo1313_2009	hypothetical protein	-0.4	3.04E-01
Clo1313_2010	hypothetical protein	0.4	3.72E-01
Clo1313_2011	hypothetical protein	-0.2	5.97E-01
Clo1313_2012	5-nitroimidazole antibiotic resistance protein	0.3	4.72E-01
Clo1313_2013	hypothetical protein	0.0	9.95E-01
Clo1313_2014	metallophosphoesterase	-0.3	3.39E-02

Clo1313_2015	Glucose-6-phosphate isomerase	-0.4	8.43E-04
Clo1313_2016	small acid-soluble spore protein alpha/beta type	0.3	4.16E-01
Clo1313_2017	phenylalanyl-tRNA synthetase beta subunit (EC 6.1.1.20)	0.2	7.18E-02
Clo1313_2018	phenylalanyl-tRNA synthetase, alpha subunit (EC 6.1.1.20)	0.4	2.97E-03
Clo1313_2019	protein of unknown function DUF975	-0.2	6.60E-02
Clo1313_2020	broad-specificity cellobiase (EC 3.2.1.21)	-0.1	4.87E-01
Clo1313_2021	hypothetical protein	-0.1	8.40E-01
Clo1313_2022	glycoside hydrolase family 16	-0.3	1.40E-01
Clo1313_2023	transcriptional regulator, LacI family	0.1	3.81E-01
Clo1313_2024	glycosyl transferase family 2	0.6	6.34E-02
Clo1313_2025	Pseudo gene	0.5	1.24E-07
Clo1313_2026	hypothetical protein	0.4	1.03E-02
Clo1313_2027	UvrD/REP helicase	0.4	1.23E-02
Clo1313_2028	hypothetical protein	0.0	8.54E-01
Clo1313_2029	protein of unknown function DUF1294	0.2	4.82E-01
Clo1313_2030	response regulator receiver and ANTAR domain protein	0.5	7.76E-02
Clo1313_2031	L-glutamine synthetase (EC 6.3.1.2)	0.3	1.59E-02
Clo1313_2032	glutamate synthase (NADPH) GltB3 subunit (EC 1.4.1.13)	-0.3	1.33E-02
Clo1313_2033	FAD-dependent pyridine nucleotide-disulfide oxidoreductase	-0.5	1.87E-04
Clo1313_2034	iron-sulfur cluster-binding protein	0.2	5.79E-01
Clo1313_2035	glutamate synthase (NADPH) GltB2 subunit (EC 1.4.1.13)	0.8	2.02E-03
Clo1313_2036	glutamine amidotransferase class-II	0.6	4.29E-02
Clo1313_2037	aspartate-ammonia ligase (EC 6.3.1.1) Pseudo gene	-0.1	8.57E-01
Clo1313_2038	glutamine synthetase catalytic region	0.4	6.88E-03
Clo1313_2039	Pseudo gene	0.3	7.03E-02
Clo1313_2040	hypothetical protein	-0.1	2.98E-01
Clo1313_2041	sodium/proton antiporter, CPA1 family (TC 2.A.36)	-0.7	8.30E-08
Clo1313_2042	proteinase inhibitor I4 serpin	0.4	2.74E-02
Clo1313_2043	proteinase inhibitor I4 serpin	0.5	6.77E-05
Clo1313_2044	non-canonical purine NTP pyrophosphatase, rdgB/HAM1 family	0.0	9.33E-01
Clo1313_2045	ribonuclease PH	0.1	4.45E-01
Clo1313_2046	Lipoprotein LpqB, GerMN domain	-0.2	2.39E-02
Clo1313_2047	UDP-galactose 4-epimerase (EC 5.1.3.2)	0.0	9.65E-01
Clo1313_2048	cell wall hydrolase/autolysin	0.7	4.76E-19
Clo1313_2049	type IV pilus assembly protein PilM	0.0	8.65E-01
Clo1313_2050	protein of unknown function DUF208	0.0	9.78E-01
Clo1313_2051	Holliday junction DNA helicase subunit RuvB	0.1	6.57E-01
Clo1313_2052	Holliday junction DNA helicase subunit RuvA	-0.1	8.20E-01
Clo1313_2053	Holliday junction endonuclease RuvC (EC 3.1.22.4)	0.1	5.69E-01
Clo1313_2054	argininosuccinate synthase (EC 6.3.4.5)	0.0	8.33E-01
Clo1313_2055	argininosuccinate lyase	-0.3	4.31E-04
Clo1313_2056	metal-dependent phosphohydrolase, HD region	0.3	4.24E-01
Clo1313_2057	hypothetical protein	0.1	6.02E-01
Clo1313_2058	polysaccharide deacetylase	0.1	4.44E-01
Clo1313_2059	sulfatase	0.3	4.69E-04
Clo1313_2060	FAD-dependent pyridine nucleotide-disulfide oxidoreductase	0.2	6.56E-01
Clo1313_2061	hypothetical protein	NA	NA
Clo1313_2062	hypothetical protein	0.1	7.05E-01
Clo1313_2063	hypothetical protein	0.0	9.57E-01
Clo1313_2064	FAD dependent oxidoreductase	0.5	2.19E-01
Clo1313_2065	hypothetical protein	0.7	1.04E-02
Clo1313_2066	response regulator receiver	-0.2	6.65E-01
Clo1313_2067	4Fe-4S ferredoxin iron-sulfur binding domain protein	0.5	5.54E-04
Clo1313_2068	hypothetical protein	0.3	1.85E-01
Clo1313_2069	protein of unknown function UPF0044	0.0	8.49E-01
Clo1313_2070	hypothetical protein	-0.4	4.83E-03
Clo1313_2071	GTP-binding protein Obg/CgtA	-0.2	2.96E-02
Clo1313_2072	LSU ribosomal protein L27P	-0.1	4.87E-01
Clo1313_2073	protein of unknown function DUF464	-0.5	3.37E-08
Clo1313_2074	LSU ribosomal protein L21P	-0.3	4.81E-04
Clo1313_2075	RNAse G (EC 3.1.4.-)	-0.4	7.90E-06
Clo1313_2076	Protein of unknown function DUF2344	-0.3	3.63E-02
Clo1313_2077	Radical SAM domain protein	-0.3	8.01E-04
Clo1313_2078	hypothetical protein	-0.3	1.17E-02
Clo1313_2079	protein of unknown function DUF1292	-0.3	1.72E-02
Clo1313_2080	Holliday junction resolvase YqgF	0.0	8.92E-01
Clo1313_2081	aldo/keto reductase	0.0	8.40E-01
Clo1313_2082	protein of unknown function DUF965	-0.2	2.08E-01
Clo1313_2083	RNA modification enzyme, MiaB family	0.2	1.70E-01
Clo1313_2084	phosphoryl transfer system HPr	0.6	1.32E-02
Clo1313_2085	protein of unknown function DUF523	0.3	5.58E-02
Clo1313_2086	RNAse R (EC 3.1.-.-)	-0.1	6.89E-01
Clo1313_2087	transposase IS200-family protein	-0.2	6.74E-01
Clo1313_2088	metal dependent phosphohydrolase	0.3	5.83E-02
Clo1313_2089	preprotein translocase, SecG subunit	0.7	4.04E-08
Clo1313_2090	enolase	0.0	7.31E-01
Clo1313_2091	diguanylate cyclase and metal dependent phosphohydrolase	1.0	2.30E-07
Clo1313_2092	phosphoglycerate mutase (EC 5.4.2.1)	-0.2	3.08E-02

Clo1313_2093	triosephosphate isomerase (EC 5.3.1.1)	-0.4	3.78E-05
Clo1313_2094	phosphoglycerate kinase (EC 2.7.2.3)	-0.5	3.72E-07
Clo1313_2095	glyceraldehyde-3-phosphate dehydrogenase, type I	-0.4	3.53E-05
Clo1313_2096	4'-phosphopantetheinyl transferase	0.2	9.41E-02
Clo1313_2097	Beta-ketoacyl synthase	0.1	4.13E-01
Clo1313_2098	hypothetical protein	0.2	4.18E-01
Clo1313_2099	AMP-dependent synthetase and ligase	0.4	4.23E-02
Clo1313_2100	Beta-ketoacyl-acyl-carrier-protein synthase I	0.4	1.96E-02
Clo1313_2101	phosphopantetheine-binding protein	0.8	5.89E-04
Clo1313_2102	Beta-ketoacyl-acyl-carrier-protein synthase I	0.0	8.99E-01
Clo1313_2103	diguanylate cyclase and metal dependent phosphohydrolase	-0.1	3.35E-01
Clo1313_2104	RNA methyltransferase, TrmH family, group 2	0.0	9.97E-01
Clo1313_2105	CheC domain protein	-0.3	4.99E-01
Clo1313_2106	stage V sporulation protein ae	0.1	8.72E-01
Clo1313_2107	stage V sporulation protein AE	0.0	9.31E-01
Clo1313_2108	stage V sporulation protein AD	0.1	8.90E-01
Clo1313_2109	stage V sporulation protein AC	0.1	7.66E-01
Clo1313_2110	hypothetical protein	NA	NA
Clo1313_2111	RNA polymerase, sigma subunit, RpoX/SigF	0.1	7.51E-01
Clo1313_2112	anti-sigma regulatory factor, serine/threonine protein kinase	0.4	2.35E-01
Clo1313_2113	anti-anti-sigma regulatory factor, SpoIIAA	0.4	3.21E-01
Clo1313_2114	Phosphotransferase system, phosphocarrier protein HPr	0.1	6.20E-01
Clo1313_2115	protein of unknown function DUF199	-0.4	2.71E-03
Clo1313_2116	glycogen debranching enzyme	-0.3	4.90E-03
Clo1313_2117	protein of unknown function UPF0052 and CofD	-0.4	1.92E-03
Clo1313_2118	hypothetical protein	-0.3	9.04E-02
Clo1313_2119	UDP-N-acetylmuramate dehydrogenase (EC 1.1.1.158)	-0.3	8.85E-02
Clo1313_2120	PHP domain protein	-0.3	6.29E-02
Clo1313_2121	Hpr(Ser) kinase/phosphatase (EC 2.7.1.-)	-0.1	7.75E-01
Clo1313_2122	Dockerin type 1	0.4	2.15E-01
Clo1313_2123	type IV pilus assembly PilZ	0.9	3.99E-07
Clo1313_2124	6,7-dimethyl-8-ribityllumazine synthase (EC 2.5.1.78)	0.8	1.37E-05
Clo1313_2125	3,4-dihydroxy-2-butanone 4-phosphate synthase	0.4	3.36E-04
Clo1313_2126	riboflavin synthase alpha chain (EC 2.5.1.9)	-0.1	7.18E-01
Clo1313_2127	riboflavin biosynthesis protein RibD	0.0	9.95E-01
Clo1313_2128	protein of unknown function UPF0047	0.2	5.39E-01
Clo1313_2129	tRNA (guanine-N(7)-)-methyltransferase (EC 2.1.1.33)	0.4	3.18E-02
Clo1313_2130	iron-containing alcohol dehydrogenase	-0.1	5.78E-01
Clo1313_2131	hypothetical protein	-0.1	4.13E-01
Clo1313_2132	5,10-methylenetetrahydrofolate reductase (NAD(P)) (EC 1.5.1.20)	0.2	1.89E-01
Clo1313_2133	peptidase M50	0.4	1.25E-01
Clo1313_2134	Peptidase M23	0.6	4.01E-02
Clo1313_2135	protein of unknown function UPF0180	0.2	5.20E-01
Clo1313_2136	methylglyoxal synthase	-0.2	5.83E-02
Clo1313_2137	cell division topological specificity factor MinE	-0.4	1.89E-05
Clo1313_2138	septum site-determining protein MinD	-0.1	1.74E-01
Clo1313_2139	septum site-determining protein MinC	0.3	1.40E-01
Clo1313_2140	penicillin-binding protein 2	-0.1	4.46E-01
Clo1313_2141	rod shape-determining protein MreD	0.3	1.52E-01
Clo1313_2142	rod shape-determining protein MreC	0.0	7.63E-01
Clo1313_2143	rod shape-determining protein MreB	0.5	2.51E-06
Clo1313_2144	maf protein	0.6	1.12E-07
Clo1313_2145	hypothetical protein	0.0	9.18E-01
Clo1313_2146	Negative regulator of genetic competence	0.4	3.48E-04
Clo1313_2147	hypothetical protein	0.8	3.27E-06
Clo1313_2148	protein serine/threonine phosphatase	0.0	7.86E-01
Clo1313_2149	ATP-dependent protease La	-0.5	6.06E-06
Clo1313_2150	glutamate N-acetyltransferase (EC 2.3.1.35)	-0.1	3.91E-01
Clo1313_2151	CheW protein	0.2	6.29E-01
Clo1313_2152	hypothetical protein	0.5	8.11E-02
Clo1313_2153	hypothetical protein	-0.6	9.32E-08
Clo1313_2154	hypothetical protein	0.3	1.64E-02
Clo1313_2155	hypothetical protein	0.1	2.53E-01
Clo1313_2156	hypothetical protein	0.4	2.83E-01
Clo1313_2157	hypothetical protein	0.2	3.94E-01
Clo1313_2158	Pseudo gene	-0.2	6.30E-01
Clo1313_2159	hypothetical protein	0.3	3.23E-02
Clo1313_2160	phage shock protein C (PspC) family protein	0.5	8.14E-02
Clo1313_2161	cellulose 1,4-beta-cellobiosidase (EC 3.2.1.91)	0.0	9.88E-01
Clo1313_2162	asparaginyl-tRNA synthetase (EC 6.1.1.22)	-0.2	1.03E-02
Clo1313_2163	aspartate-ammonia ligase (EC 6.3.1.1)	0.1	5.85E-01
Clo1313_2164	Peptidylprolyl isomerase	0.0	7.32E-01
Clo1313_2165	Silent information regulator protein Sir2	0.7	7.41E-06
Clo1313_2166	hypothetical protein	0.4	8.73E-05
Clo1313_2167	hypothetical protein	1.0	7.37E-10
Clo1313_2168	Na/Pi-cotransporter II-related protein	0.4	5.32E-03
Clo1313_2169	Rubredoxin-type Fe(Cys)4 protein	0.4	6.59E-05
Clo1313_2170	sulfatase	0.0	9.68E-01

Clo1313_2171	BioY protein	0.7	1.52E-05
Clo1313_2172	SEC-C motif domain protein	0.0	8.24E-01
Clo1313_2173	type 3a cellulose-binding domain protein	-0.1	7.18E-01
Clo1313_2174	RNA polymerase sigma-I factor	0.0	8.93E-01
Clo1313_2175	hypothetical protein	0.2	7.05E-01
Clo1313_2176	S-layer domain-containing protein	0.3	7.02E-03
Clo1313_2177	hypothetical protein	-0.7	2.15E-03
Clo1313_2178	protein of unknown function DUF187	-0.6	1.42E-09
Clo1313_2179	ribonucleoside-diphosphate reductase class II (EC 1.17.4.-)	-0.6	5.79E-13
Clo1313_2180	hypothetical protein	-0.3	5.58E-03
Clo1313_2181	hypothetical protein	-0.3	1.03E-01
Clo1313_2182	hypothetical protein	0.5	1.02E-01
Clo1313_2183	helix-turn-helix domain protein	0.4	5.58E-02
Clo1313_2184	copper amine oxidase-like domain-containing protein	0.7	1.50E-02
Clo1313_2185	copper amine oxidase-like domain-containing protein	0.3	4.31E-01
Clo1313_2186	hypothetical protein	0.4	4.02E-04
Clo1313_2187	copper amine oxidase-like domain-containing protein	0.9	1.45E-08
Clo1313_2188	Spore coat protein CotH	0.5	1.39E-01
Clo1313_2189	endoglucanase Cel9N	0.4	1.18E-04
Clo1313_2190	iron-only hydrogenase maturation protein HydF	-0.2	1.72E-01
Clo1313_2191	hypothetical protein	0.4	3.16E-01
Clo1313_2192	endo-1,3(4)-beta-glucanase (EC:3.2.1.6)	-0.3	5.46E-03
Clo1313_2193	methyl-accepting chemotaxis sensory transducer	0.3	9.75E-02
Clo1313_2194	CheW protein	0.9	9.49E-03
Clo1313_2195	methyl-accepting chemotaxis sensory transducer	-0.5	1.14E-07
Clo1313_2196	CheW protein	-0.4	1.81E-04
Clo1313_2197	heat shock protein Hsp20	-0.2	3.62E-01
Clo1313_2198	hybrid cluster protein	0.2	5.97E-01
Clo1313_2199	iron-sulfur binding protein	0.1	7.75E-01
Clo1313_2200	transcriptional regulator, Crp/Fnr family	0.5	4.88E-02
Clo1313_2201	small GTP-binding protein	-0.2	2.44E-01
Clo1313_2202	Dockerin type 1	0.5	5.67E-05
Clo1313_2203	Protein of unknown function DUF2174-like protein	0.3	1.46E-01
Clo1313_2204	Nucleotidyltransferase, predicted	-0.2	4.11E-01
Clo1313_2205	Pseudo gene	0.4	2.29E-01
Clo1313_2206	Pseudo gene	-0.1	7.47E-01
Clo1313_2207	adenosylmethionine-8-amino-7-oxononanoate aminotransferase	0.3	4.63E-01
Clo1313_2208	biotin biosynthesis protein BioC	-0.3	4.72E-01
Clo1313_2209	Carboxylesterase	0.1	9.08E-01
Clo1313_2210	8-amino-7-oxononanoate synthase	-0.2	6.19E-01
Clo1313_2211	dethiobiotin synthase	-0.3	5.19E-01
Clo1313_2212	biotin synthase (EC 2.8.1.6)	-0.6	6.05E-02
Clo1313_2213	Pseudo gene	-0.3	2.17E-02
Clo1313_2214	protein of unknown function DUF1538	-0.7	3.16E-15
Clo1313_2215	Ferritin Dps family protein	-1.1	8.75E-58
Clo1313_2216	alpha-L-arabinofuranosidase B	0.1	5.07E-01
Clo1313_2217	hypothetical protein	0.7	4.49E-02
Clo1313_2218	Pseudo gene	-0.3	2.94E-01
Clo1313_2219	hypothetical protein	0.1	8.33E-01
Clo1313_2220	Pseudo gene	-0.1	2.76E-01
Clo1313_2221	hypothetical protein	0.0	9.95E-01
Clo1313_2222	hypothetical protein	0.5	1.53E-01
Clo1313_2223	hypothetical protein	0.2	6.45E-01
Clo1313_2224	transposase mutator type	NA	NA
Clo1313_2225	helix-turn-helix domain protein	-0.1	6.71E-01
Clo1313_2226	protein of unknown function DUF891	0.0	9.57E-01
Clo1313_2227	Pseudo gene	0.0	8.65E-01
Clo1313_2228	alkyl hydroperoxide reductase/ Thiol specific antioxidant/ Mal allergen	0.1	4.85E-01
Clo1313_2229	protein of unknown function DUF1706	0.1	8.57E-01
Clo1313_2230	Pseudo gene	0.1	6.92E-01
Clo1313_2231	hypothetical protein	0.0	9.74E-01
Clo1313_2232	RNA polymerase, sigma-24 subunit, ECF subfamily	0.1	7.58E-01
Clo1313_2233	glycoside hydrolase family 5	0.0	8.33E-01
Clo1313_2234	glycoside hydrolase family 5	-0.2	9.68E-02
Clo1313_2235	NADPH-dependent FMN reductase	0.0	7.91E-01
Clo1313_2236	Pseudo gene	0.5	7.39E-02
Clo1313_2237	hypothetical protein	0.9	1.03E-08
Clo1313_2238	hypothetical protein	0.8	3.75E-11
Clo1313_2239	dCTP deaminase (EC 3.5.4.13)	0.6	9.05E-08
Clo1313_2240	transcriptional regulator, TetR family	0.1	7.27E-01
Clo1313_2241	hypothetical protein	0.2	5.10E-01
Clo1313_2242	hypothetical protein	0.3	5.51E-01
Clo1313_2243	hypothetical protein	0.3	1.43E-01
Clo1313_2244	Cof-like hydrolase	0.7	1.15E-07
Clo1313_2245	hypothetical protein	0.5	1.65E-03
Clo1313_2246	transcriptional regulator	0.3	1.52E-02
Clo1313_2247	beta-lactamase domain protein	0.1	4.81E-01

Clo1313_2248	NUDIX hydrolase	-0.3	4.74E-01
Clo1313_2249	hypothetical protein	0.0	9.13E-01
Clo1313_2250	methyl-accepting chemotaxis sensory transducer	-0.4	3.69E-03
Clo1313_2251	hypothetical protein	0.2	6.84E-01
Clo1313_2252	hypothetical protein	-0.2	5.62E-01
Clo1313_2253	transcriptional regulator, PadR family	-0.1	7.90E-01
Clo1313_2254	NAD(P)H dehydrogenase (quinone)	-0.5	6.40E-05
Clo1313_2255	transcriptional regulator, PadR family	-0.5	3.70E-04
Clo1313_2256	protein of unknown function DUF6 transmembrane	0.1	7.10E-01
Clo1313_2257	pyridoxamine 5'-phosphate oxidase-related FMN-binding protein	-0.2	6.07E-02
Clo1313_2258	hypothetical protein	0.5	1.81E-01
Clo1313_2259	methyl-accepting chemotaxis sensory transducer	0.9	6.18E-10
Clo1313_2260	ammonium transporter (TC 1.A.11)	0.7	1.21E-02
Clo1313_2261	ABC transporter transmembrane region	-0.2	7.25E-02
Clo1313_2262	ABC transporter transmembrane region	-0.2	2.23E-01
Clo1313_2263	transcriptional regulator, MarR family	-0.4	7.06E-02
Clo1313_2264	hypothetical protein	-0.2	9.24E-03
Clo1313_2265	Linocin_M18 bacteriocin protein	-0.3	6.62E-03
Clo1313_2266	S-layer domain-containing protein	0.6	5.24E-03
Clo1313_2267	metal dependent phosphohydrolase	0.5	6.81E-03
Clo1313_2268	Pseudo gene	0.8	3.28E-02
Clo1313_2269	AIG2 family protein	0.3	4.71E-01
Clo1313_2270	arsenate reductase-like protein	0.4	6.99E-02
Clo1313_2271	protein of unknown function DUF438	0.1	6.38E-01
Clo1313_2272	short-chain dehydrogenase/reductase SDR	-0.4	6.08E-03
Clo1313_2273	DNA methylase N-4/N-6 domain protein	-0.2	9.97E-02
Clo1313_2274	Type II site-specific deoxyribonuclease	0.1	7.53E-01
Clo1313_2275	DNA adenine methylase	0.0	8.86E-01
Clo1313_2276	protein of unknown function DUF125 transmembrane	-0.3	2.20E-02
Clo1313_2277	Pseudo gene	0.4	2.79E-01
Clo1313_2278	ribosome small subunit-dependent GTPase A	0.5	1.63E-01
Clo1313_2279	hypothetical protein	-0.1	7.53E-01
Clo1313_2280	type I phosphodiesterase/nucleotide pyrophosphatase	0.2	3.24E-01
Clo1313_2281	Pseudo gene	NA	NA
Clo1313_2282	hypothetical protein	0.6	1.32E-02
Clo1313_2283	SAM-dependent methyltransferase	-0.1	5.10E-01
Clo1313_2284	hypothetical protein	-0.4	3.78E-03
Clo1313_2285	hypothetical protein	-0.1	8.36E-01
Clo1313_2286	copper amine oxidase-like domain-containing protein	-0.1	7.14E-01
Clo1313_2287	hypothetical protein	0.2	5.17E-01
Clo1313_2288	NADPH-dependent FMN reductase	-0.3	3.71E-01
Clo1313_2289	glycosyl transferase group 1	0.1	7.24E-01
Clo1313_2290	hypothetical protein	0.9	3.38E-17
Clo1313_2291	protein of unknown function DUF214	-0.1	8.00E-01
Clo1313_2292	ABC transporter related protein	0.1	8.20E-01
Clo1313_2293	integral membrane sensor signal transduction histidine kinase	-0.4	2.95E-02
Clo1313_2294	two component transcriptional regulator, winged helix family	-0.3	3.75E-01
Clo1313_2295	Pseudo gene	0.3	1.72E-01
Clo1313_2296	flavin reductase domain protein FMN-binding protein	0.4	2.88E-01
Clo1313_2297	Pseudo gene	-0.1	6.97E-01
Clo1313_2298	hypothetical protein	0.6	1.45E-02
Clo1313_2299	hypothetical protein	0.1	9.01E-01
Clo1313_2300	ABC transporter related protein	0.2	5.78E-01
Clo1313_2301	hypothetical protein	0.1	6.92E-01
Clo1313_2302	helix-turn-helix domain protein	0.2	5.45E-01
Clo1313_2303	glutamine synthetase catalytic region	0.6	7.09E-02
Clo1313_2304	aspartyl/glutamyl-tRNA(Asn/Gln) amidotransferase subunit B (EC 6.3.5.-)	0.7	1.25E-02
Clo1313_2305	aspartyl/glutamyl-tRNA(Asn/Gln) amidotransferase subunit A (EC 6.3.5.-)	0.6	1.17E-02
Clo1313_2306	aspartyl/glutamyl-tRNA(Asn/Gln) amidotransferase subunit C (EC 6.3.5.-)	0.2	6.21E-01
Clo1313_2307	aspartyl-tRNA synthetase	0.8	8.97E-05
Clo1313_2308	Pseudo gene	0.2	5.92E-01
Clo1313_2309	hypothetical protein	0.1	7.31E-01
Clo1313_2310	4Fe-4S ferredoxin iron-sulfur binding domain protein	0.4	3.41E-02
Clo1313_2311	Protein of unknown function DUF2871	0.4	3.67E-03
Clo1313_2312	Enoyl-CoA hydratase/isomerase	0.0	9.08E-01
Clo1313_2313	Pseudo gene	0.0	8.24E-01
Clo1313_2314	Methyltransferase type 12	0.1	7.78E-01
Clo1313_2315	Pseudo gene	0.2	6.92E-01
Clo1313_2316	Pseudo gene	0.4	1.12E-01
Clo1313_2317	hypothetical protein	-0.1	4.02E-01
Clo1313_2318	flavin reductase domain protein FMN-binding protein	0.0	9.59E-01
Clo1313_2319	methylated-DNA/protein-cysteine methyltransferase	0.4	2.47E-04
Clo1313_2320	Pseudo gene	0.3	4.89E-01
Clo1313_2321	NLPA lipoprotein	0.1	3.44E-01

Clo1313_2322	binding-protein-dependent transport systems inner membrane component	0.5	5.15E-07
Clo1313_2323	ABC transporter related protein	0.4	4.11E-04
Clo1313_2324	transcriptional regulator, BadM/Rrf2 family	0.1	6.54E-01
Clo1313_2325	cystathionine gamma-lyase (EC 4.4.1.1)	0.1	8.71E-01
Clo1313_2326	cystathionine beta-synthase (acetylserine-dependent) (EC 4.2.1.-)	0.6	9.80E-02
Clo1313_2327	hypothetical protein	-0.2	7.14E-01
Clo1313_2328	protein of unknown function DUF214	0.3	3.43E-01
Clo1313_2329	ABC transporter related protein	0.5	1.55E-01
Clo1313_2330	GCN5-related N-acetyltransferase	0.6	1.92E-02
Clo1313_2331	oxidoreductase/nitrogenase component 1	1.0	6.89E-06
Clo1313_2332	oxidoreductase/nitrogenase component 1	1.1	2.25E-06
Clo1313_2333	Dinitrogenase iron-molybdenum cofactor biosynthesis protein	1.1	6.92E-04
Clo1313_2334	Radical SAM domain protein	1.0	2.72E-05
Clo1313_2335	Cysteine synthase	1.2	5.92E-05
Clo1313_2336	extracellular solute-binding protein family 3	2.1	1.88E-35
Clo1313_2337	binding-protein-dependent transport systems inner membrane component	0.9	1.35E-08
Clo1313_2338	ABC transporter related protein	0.8	2.84E-04
Clo1313_2339	nitrogenase iron protein	0.9	4.22E-09
Clo1313_2340	hypothetical protein	0.2	6.44E-02
Clo1313_2341	purine nucleoside phosphorylase	-0.1	8.99E-01
Clo1313_2342	basic membrane lipoprotein	0.2	6.30E-01
Clo1313_2343	hypothetical protein	0.3	2.55E-01
Clo1313_2344	(Formate-C-acetyltransferase)-activating enzyme	0.2	6.08E-01
Clo1313_2345	ABC transporter related protein	0.2	6.70E-01
Clo1313_2346	inner-membrane translocator	0.3	5.08E-01
Clo1313_2347	inner-membrane translocator	0.3	5.70E-01
Clo1313_2348	cytidine deaminase (EC 3.5.4.5)	0.6	1.05E-01
Clo1313_2349	deoxyribose-phosphate aldolase	0.1	7.90E-01
Clo1313_2350	two component transcriptional regulator, AraC family	-0.5	9.09E-06
Clo1313_2351	signal transduction histidine kinase, LytS	-0.7	2.82E-08
Clo1313_2352	binding-protein-dependent transport systems inner membrane component	0.3	2.13E-01
Clo1313_2353	ABC transporter related protein	-0.1	6.74E-01
Clo1313_2354	extracellular solute-binding protein family 1	-0.2	4.64E-01
Clo1313_2355	transcriptional repressor, CopY family	-0.6	5.65E-02
Clo1313_2356	peptidase M56 BlaR1	-0.4	9.80E-04
Clo1313_2357	hypothetical protein	0.1	6.47E-01
Clo1313_2358	hypothetical protein	-0.1	8.64E-01
Clo1313_2359	glycerate kinase	-0.2	2.69E-01
Clo1313_2360	Pseudo gene	-0.1	7.88E-01
Clo1313_2361	hypothetical protein	-0.1	8.15E-01
Clo1313_2362	Pseudo gene	-0.4	3.38E-01
Clo1313_2363	Pseudo gene	-0.5	5.86E-02
Clo1313_2364	Pseudo gene	-0.6	5.86E-03
Clo1313_2365	PglZ domain protein	-0.5	1.38E-02
Clo1313_2366	SNF2-related protein	0.1	5.84E-01
Clo1313_2367	DNA methylase N-4/N-6 domain protein	0.2	3.79E-01
Clo1313_2368	Pseudo gene	0.1	4.22E-01
Clo1313_2369	Integrase catalytic region	-0.3	3.96E-01
Clo1313_2370	Protein of unknown function DUF2971	-0.4	6.11E-03
Clo1313_2371	HhH-GPD family protein	-0.1	7.71E-01
Clo1313_2372	hypothetical protein	-0.1	7.70E-01
Clo1313_2373	HNH endonuclease	-0.4	6.44E-02
Clo1313_2374	NUDIX hydrolase	0.0	9.82E-01
Clo1313_2375	Integrase catalytic region	-0.3	8.45E-02
Clo1313_2376	Resolvase domain protein	0.1	7.86E-01
Clo1313_2377	Recombinase	0.1	7.55E-01
Clo1313_2378	Resolvase domain protein	-0.1	7.66E-01
Clo1313_2379	hypothetical protein	NA	NA
Clo1313_2380	N-acetylmuramoyl-L-alanine amidase family 2	0.0	9.66E-01
Clo1313_2381	toxin secretion/phage lysis holin	0.0	8.24E-01
Clo1313_2382	hypothetical protein	-0.1	8.11E-01
Clo1313_2383	hypothetical protein	0.0	9.15E-01
Clo1313_2384	hypothetical protein	0.0	7.05E-01
Clo1313_2385	phage minor structural protein	0.1	8.39E-01
Clo1313_2386	phage tail component	0.1	6.31E-01
Clo1313_2387	hypothetical protein	0.1	8.55E-01
Clo1313_2388	hypothetical protein	NA	NA
Clo1313_2389	hypothetical protein	0.1	7.97E-01
Clo1313_2390	phage major tail protein, phi13 family	0.1	7.91E-01
Clo1313_2391	hypothetical protein	0.2	5.73E-01
Clo1313_2392	phage protein, HK97 gp10 family	-0.5	1.57E-01
Clo1313_2393	head-tail joining family protein	-0.3	3.79E-01
Clo1313_2394	Bacteriophage QLRG family, putative DNA packaging	-0.1	6.27E-01
Clo1313_2395	phage major capsid protein, HK97 family	-0.1	4.94E-01
Clo1313_2396	peptidase S14 ClpP	0.0	9.04E-01

Clo1313_2397	phage portal protein, HK97 family	0.1	5.22E-01
Clo1313_2398	Terminase	0.3	4.52E-01
Clo1313_2399	hypothetical protein	-0.1	6.69E-01
Clo1313_2400	AIG2 family protein	0.3	4.54E-01
Clo1313_2401	Putative amidoligase enzyme	0.0	9.54E-01
Clo1313_2402	hypothetical protein	NA	NA
Clo1313_2403	hypothetical protein	NA	NA
Clo1313_2404	hypothetical protein	-0.4	1.22E-01
Clo1313_2405	DNA methylase N-4/N-6 domain protein	-0.2	7.17E-01
Clo1313_2406	DNA methylase N-4/N-6 domain protein	0.0	9.65E-01
Clo1313_2407	hypothetical protein	-0.1	7.83E-01
Clo1313_2408	HNH endonuclease	0.1	6.92E-01
Clo1313_2409	hypothetical protein	-0.1	6.61E-01
Clo1313_2410	hypothetical protein	-0.1	8.68E-01
Clo1313_2411	VRR-NUC domain-containing protein	0.0	9.29E-01
Clo1313_2412	hypothetical protein	0.3	3.08E-01
Clo1313_2413	Pseudo gene	NA	NA
Clo1313_2414	ABC transporter related protein	-0.5	1.12E-01
Clo1313_2415	protein of unknown function DUF214	0.2	4.24E-01
Clo1313_2416	integral membrane sensor signal transduction histidine kinase	0.5	1.10E-02
Clo1313_2417	two component transcriptional regulator, winged helix family	0.4	6.94E-02
Clo1313_2418	hypothetical protein	0.5	3.00E-04
Clo1313_2419	Pseudo gene	0.0	7.92E-01
Clo1313_2420	Pseudo gene	0.1	7.54E-01
Clo1313_2421	hypothetical protein	-0.2	2.76E-01
Clo1313_2422	hypothetical protein	-0.3	2.76E-02
Clo1313_2423	hypothetical protein	0.1	5.82E-01
Clo1313_2424	hypothetical protein	NA	NA
Clo1313_2425	23S rRNA m(5)U-1939 methyltransferase (EC 2.1.1.190)	0.1	6.26E-01
Clo1313_2426	ABC transporter related protein	-0.1	6.02E-01
Clo1313_2427	transport system permease protein	-0.1	8.17E-01
Clo1313_2428	periplasmic binding protein	0.2	3.08E-01
Clo1313_2429	hypothetical protein	-0.1	3.17E-01
Clo1313_2430	Peptidase M23	-0.4	2.65E-05
Clo1313_2431	protein of unknown function DUF583	-0.8	2.23E-07
Clo1313_2432	sporulation protein YunB	0.1	8.37E-01
Clo1313_2433	penicillin-binding protein, 1A family	-0.3	2.23E-04
Clo1313_2434	protein of unknown function DUF214	0.2	1.31E-01
Clo1313_2435	efflux transporter, RND family, MFP subunit	-0.3	1.29E-04
Clo1313_2436	ABC transporter related protein	-0.2	7.21E-02
Clo1313_2437	outer membrane efflux protein	0.0	9.15E-01
Clo1313_2438	hypothetical protein	0.1	3.30E-01
Clo1313_2439	glutamate 5-kinase (EC 2.7.2.11)	0.2	5.83E-02
Clo1313_2440	hypothetical protein	-0.5	1.65E-08
Clo1313_2441	NifU-like domain-containing protein	-0.9	6.55E-21
Clo1313_2442	hypothetical protein	0.3	5.22E-01
Clo1313_2443	hypothetical protein	-0.2	3.39E-01
Clo1313_2444	Rubrerythrin	0.2	8.55E-02
Clo1313_2445	protein of unknown function DUF218	-0.1	5.79E-01
Clo1313_2446	peptidase S16, lon-like protein	0.0	9.05E-01
Clo1313_2447	[SSU ribosomal protein S18P]-alanine acetyltransferase (EC 2.3.1.128)	0.0	9.60E-01
Clo1313_2448	peptidase M22 glycoprotease	-0.4	2.03E-02
Clo1313_2449	Uncharacterized protein family UPF0079, ATPase	-0.4	1.75E-02
Clo1313_2450	amidohydrolase	-0.2	1.73E-01
Clo1313_2451	copper amine oxidase-like domain-containing protein	-0.3	1.82E-03
Clo1313_2452	hypothetical protein	0.4	8.50E-02
Clo1313_2453	hypothetical protein	0.1	7.46E-01
Clo1313_2454	hypothetical protein	0.1	8.04E-01
Clo1313_2455	SSU ribosomal protein S9P	-0.1	3.10E-01
Clo1313_2456	LSU ribosomal protein L13P	-0.2	6.34E-02
Clo1313_2457	hypothetical protein	-0.1	5.34E-01
Clo1313_2458	DNA integrity scanning, DisA, linker region	0.0	8.93E-01
Clo1313_2459	DNA repair protein RadA	0.1	5.76E-01
Clo1313_2460	glycoside hydrolase 15-related protein	0.0	8.20E-01
Clo1313_2461	glycosyl transferase family 2	-0.1	8.29E-01
Clo1313_2462	ATPase AAA-2 domain protein	-0.2	9.46E-02
Clo1313_2463	ATP:guanido phosphotransferase	0.1	6.19E-01
Clo1313_2464	UvrB/UvrC protein	0.1	4.89E-01
Clo1313_2465	transcriptional repressor, CtsR	-0.6	2.78E-09
Clo1313_2466	Pseudo gene	-0.3	3.02E-02
Clo1313_2467	translation elongation factor G	0.6	9.08E-08
Clo1313_2468	3-deoxy-D-arabinoheptulosonate-7-phosphate synthase (EC 2.5.1.54)	0.1	2.76E-01
Clo1313_2469	Prephenate dehydrogenase	0.1	5.14E-01
Clo1313_2470	3-phosphoshikimate 1-carboxyvinyltransferase (EC 2.5.1.19)	0.2	5.58E-02
Clo1313_2471	CoA-binding domain protein	-1.3	9.64E-41
Clo1313_2472	ABC transporter related protein	-0.5	3.87E-09

Clo1313_2473	Peptidoglycan-binding lysin domain	0.6	1.91E-02
Clo1313_2474	ABC transporter related protein	0.3	1.36E-01
Clo1313_2475	cobalt ABC transporter, inner membrane subunit CbiQ	0.0	9.95E-01
Clo1313_2476	cobalamin (vitamin B12) biosynthesis CbiM protein	-0.4	1.10E-03
Clo1313_2477	cell wall hydrolase/autolysin	0.2	1.55E-01
Clo1313_2478	response regulator receiver modulated diguanylate cyclase	0.3	6.02E-03
Clo1313_2479	Ig domain protein group 2 domain protein	0.1	1.76E-01
Clo1313_2480	Pseudo gene	0.4	7.46E-04
Clo1313_2481	hypothetical protein	-0.2	4.72E-01
Clo1313_2482	hypothetical protein	-0.1	8.92E-01
Clo1313_2483	RNA polymerase, sigma-24 subunit, ECF subfamily	-0.1	8.92E-01
Clo1313_2484	peptidase M56 BlaR1	-0.2	4.00E-01
Clo1313_2485	transcriptional repressor, CopY family	0.0	9.65E-01
Clo1313_2486	Urease accessory protein UreD	0.0	9.27E-01
Clo1313_2487	urease accessory protein UreG	0.6	9.03E-02
Clo1313_2488	Urease accessory protein UreF	0.3	4.72E-01
Clo1313_2489	Pseudo gene	0.5	2.47E-01
Clo1313_2490	urease, alpha subunit	0.3	4.75E-01
Clo1313_2491	urease, beta subunit	0.3	5.02E-01
Clo1313_2492	urease, gamma subunit	0.1	8.91E-01
Clo1313_2493	urea ABC transporter, ATP-binding protein UrtE	0.4	2.99E-01
Clo1313_2494	urea ABC transporter, ATP-binding protein UrtD	0.3	4.13E-01
Clo1313_2495	urea ABC transporter membrane protein	0.5	2.10E-01
Clo1313_2496	urea ABC transporter, permease protein UrtB	-0.1	8.90E-01
Clo1313_2497	urea-binding protein	-0.1	8.38E-01
Clo1313_2498	two component transcriptional regulator, AraC family	0.1	8.30E-01
Clo1313_2499	multi-sensor hybrid histidine kinase	0.2	4.38E-01
Clo1313_2500	response regulator receiver sensor signal transduction histidine kinase	0.0	8.97E-01
Clo1313_2501	Pseudo gene	0.2	6.49E-01
Clo1313_2502	copper amine oxidase-like domain-containing protein	0.1	6.43E-01
Clo1313_2503	hypothetical protein	0.0	9.65E-01
Clo1313_2504	hypothetical protein	0.4	8.53E-02
Clo1313_2505	Pseudo gene	-0.2	4.11E-01
Clo1313_2506	hypothetical protein	NA	NA
Clo1313_2507	hypothetical protein	-0.6	6.15E-02
Clo1313_2508	transposase mutator type	NA	NA
Clo1313_2509	hypothetical protein	0.0	9.05E-01
Clo1313_2510	copper amine oxidase-like domain-containing protein	-0.1	3.93E-01
Clo1313_2511	hypothetical protein	0.0	9.97E-01
Clo1313_2512	Pseudo gene	0.0	9.52E-01
Clo1313_2515	hypothetical protein	-0.3	7.88E-02
Clo1313_2516	hypothetical protein	-0.5	9.09E-03
Clo1313_2517	Pseudo gene	0.2	6.57E-01
Clo1313_2518	hypothetical protein	0.1	4.89E-01
Clo1313_2519	copper amine oxidase-like domain-containing protein	0.1	4.03E-01
Clo1313_2520	hypothetical protein	0.5	2.64E-04
Clo1313_2521	hypothetical protein	0.7	1.68E-10
Clo1313_2522	transposase IS4 family protein	-0.5	4.24E-05
Clo1313_2523	hypothetical protein	0.0	7.05E-01
Clo1313_2524	hypothetical protein	-0.4	2.03E-04
Clo1313_2525	copper amine oxidase-like domain-containing protein	-0.2	1.59E-01
Clo1313_2526	hypothetical protein	-0.2	2.28E-01
Clo1313_2527	Pseudo gene	-0.1	7.43E-01
Clo1313_2528	hypothetical protein	0.2	7.32E-01
Clo1313_2529	hypothetical protein	-0.1	5.43E-01
Clo1313_2530	glycoside hydrolase family 10	0.1	6.61E-01
Clo1313_2531	iron-only hydrogenase maturation protein HydE	-0.8	1.55E-06
Clo1313_2532	cysteine synthase A	0.0	9.97E-01
Clo1313_2533	PP-loop domain protein	0.1	8.22E-01
Clo1313_2534	O-acetylhomoserine sulfhydrylase (EC 2.5.1.49)	0.2	6.15E-01
Clo1313_2535	transcriptional regulator, BadM/Rrf2 family	0.0	9.34E-01
Clo1313_2536	transcriptional regulator, BadM/Rrf2 family	0.0	9.68E-01
Clo1313_2537	homoserine O-succinyltransferase (EC 2.3.1.46)	-0.1	6.63E-01
Clo1313_2538	integral membrane sensor signal transduction histidine kinase	0.1	5.62E-01
Clo1313_2539	two component transcriptional regulator, winged helix family	-0.4	4.06E-02
Clo1313_2540	copper-translocating P-type ATPase	-0.6	1.47E-15
Clo1313_2541	Heavy metal transport/detoxification protein	-0.5	1.22E-02
Clo1313_2542	hypothetical protein	-0.8	1.87E-04
Clo1313_2543	protein of unknown function DUF1113	-0.2	3.19E-01
Clo1313_2544	Hsp33 protein	-0.4	2.30E-04
Clo1313_2545	cold-shock DNA-binding protein family	-0.4	4.31E-02
Clo1313_2546	methylated-DNA/protein-cysteine methyltransferase	-0.4	7.92E-02
Clo1313_2547	Methyltransferase type 11	0.0	9.35E-01
Clo1313_2548	small acid-soluble spore protein beta	-0.1	7.66E-01
Clo1313_2549	carboxyl-terminal protease	-0.3	3.55E-03
Clo1313_2550	Peptidase M23	-0.6	1.20E-11
Clo1313_2551	protein of unknown function DUF214	-0.2	9.75E-02

Clo1313_2552	cell division ATP-binding protein FtsE	-0.3	3.32E-03
Clo1313_2553	transcriptional regulator, CdaR family	-0.2	9.44E-03
Clo1313_2554	carbohydrate ABC transporter ATP-binding protein, CUT1 family (TC 3.A.1.1.-)	0.2	1.02E-01
Clo1313_2555	N-acetyl-gamma-glutamyl-phosphate reductase (EC 1.2.1.38)	-0.3	2.51E-02
Clo1313_2556	N-acetylglutamate kinase (EC 2.7.2.8)	-0.2	2.63E-02
Clo1313_2557	type IV pilus assembly PilZ	-0.3	1.97E-02
Clo1313_2558	acetylornithine aminotransferase apoenzyme (EC 2.6.1.11)	-0.3	1.53E-03
Clo1313_2559	carbamoyl-phosphate synthase small subunit	-0.5	2.26E-05
Clo1313_2560	carbamoyl-phosphate synthase, large subunit	-0.7	3.10E-10
Clo1313_2561	ornithine carbamoyltransferase	-0.9	3.77E-27
Clo1313_2562	GCN5-related N-acetyltransferase	-0.8	6.34E-14
Clo1313_2563	transposase IS4 family protein	-0.5	1.05E-03
Clo1313_2564	Dockerin type 1	0.8	4.91E-07
Clo1313_2565	hypothetical protein	0.4	8.30E-03
Clo1313_2566	Pseudo gene	0.4	1.59E-04
Clo1313_2567	cell division membrane protein-like protein	0.5	1.01E-03
Clo1313_2568	transcriptional regulator, PadR family	0.3	2.72E-02
Clo1313_2569	metallophosphoesterase	0.1	6.47E-01
Clo1313_2570	hypothetical protein	0.1	7.82E-01
Clo1313_2571	Pseudo gene	0.4	1.75E-01
Clo1313_2572	Serine-type D-Ala-D-Ala carboxypeptidase	-0.2	1.35E-01
Clo1313_2573	Pseudo gene	-0.1	7.57E-01
Clo1313_2574	transposase mutator type	NA	NA
Clo1313_2575	Pyridoxal-5'-phosphate-dependent protein beta subunit	-0.2	6.27E-01
Clo1313_2576	polysaccharide deacetylase	-0.2	6.99E-01
Clo1313_2577	amino acid adenylation domain protein	-0.3	3.66E-02
Clo1313_2578	glycosyl transferase family 28	-0.2	4.18E-01
Clo1313_2579	glycosyl transferase family 28	-0.1	7.91E-01
Clo1313_2580	amino acid adenylation domain protein	-0.6	5.05E-09
Clo1313_2581	copper amine oxidase-like domain-containing protein	-0.5	2.01E-08
Clo1313_2582	copper amine oxidase-like domain-containing protein	-0.1	7.05E-01
Clo1313_2583	hypothetical protein	-0.1	7.10E-01
Clo1313_2584	Carbohydrate binding family 6	-0.7	6.52E-18
Clo1313_2585	transposase IS200-family protein	NA	NA
Clo1313_2586	copper amine oxidase-like domain-containing protein	-0.3	3.39E-03
Clo1313_2587	hypothetical protein	0.2	1.90E-01
Clo1313_2588	Tetratricopeptide TPR_1 repeat-containing protein	-0.1	3.07E-01
Clo1313_2589	integral membrane sensor signal transduction histidine kinase	0.3	7.10E-02
Clo1313_2590	two component transcriptional regulator, winged helix family	0.7	3.62E-07
Clo1313_2591	ATPase, P-type (transporting), HAD superfamily, subfamily IC	0.3	2.83E-02
Clo1313_2592	arginine decarboxylase (EC 4.1.1.19)	-0.3	3.72E-03
Clo1313_2593	MgtC/SapB transporter	0.4	1.01E-01
Clo1313_2594	stage II sporulation protein R	0.3	5.29E-01
Clo1313_2595	transcriptional regulator, PadR-like family	-0.1	7.39E-01
Clo1313_2596	hypothetical protein	0.1	7.70E-01
Clo1313_2597	CTP synthase (EC 6.3.4.2)	-0.3	1.40E-02
Clo1313_2598	Pseudo gene	0.2	4.06E-01
Clo1313_2599	hypothetical protein	0.0	9.09E-01
Clo1313_2600	Hedgehog/intein hint domain protein	0.3	1.57E-01
Clo1313_2601	carboxyl-terminal protease	0.0	8.10E-01
Clo1313_2602	S-layer domain-containing protein	-0.1	3.95E-01
Clo1313_2603	S-layer domain-containing protein	1.0	1.11E-11
Clo1313_2604	Zn-dependent hydrolase of the beta-lactamase fold-like protein	-0.4	7.76E-05
Clo1313_2605	Abortive infection protein	0.2	4.10E-01
Clo1313_2606	arginyl-tRNA synthetase (EC 6.1.1.19)	-0.2	1.10E-02
Clo1313_2607	Domain of unknown function DUF1934	-0.3	1.33E-01
Clo1313_2608	glutamate racemase (EC 5.1.1.3)	-0.2	5.22E-02
Clo1313_2609	D-alanine/D-alanine ligase	-0.2	4.31E-02
Clo1313_2610	magnesium transporter	-0.2	8.83E-02
Clo1313_2611	hypothetical protein	0.2	7.04E-01
Clo1313_2612	RDD domain containing protein	-0.2	3.25E-02
Clo1313_2613	regulatory protein, FmdB family	-0.3	1.77E-01
Clo1313_2614	Lipoprotein LpqB, GerMN domain	-0.5	2.83E-04
Clo1313_2615	deoxyribose-phosphate aldolase	-0.6	2.37E-13
Clo1313_2616	protein of unknown function DUF458	-1.1	7.20E-22
Clo1313_2617	FAD-dependent pyridine nucleotide-disulfide oxidoreductase	0.3	1.83E-02
Clo1313_2618	FAD-dependent pyridine nucleotide-disulfide oxidoreductase	0.0	8.58E-01
Clo1313_2619	alkyl hydroperoxide reductase/ Thiol specific antioxidant/ Mal allergen	-0.2	2.33E-02
Clo1313_2620	cytochrome c biogenesis protein transmembrane region	-0.2	1.51E-01
Clo1313_2621	hypothetical protein	-0.1	8.79E-01
Clo1313_2622	hypothetical protein	0.1	7.32E-01
Clo1313_2623	hypothetical protein	0.3	2.60E-01
Clo1313_2624	spore cortex-lytic enzyme	-0.1	7.01E-01
Clo1313_2625	hypothetical protein	0.0	9.44E-01
Clo1313_2626	Tex-like protein	-0.4	8.86E-04
Clo1313_2627	1-phosphofructokinase	0.0	8.15E-01

Clo1313_2628	carbohydrate ABC transporter substrate-binding protein, CUT1 family (TC 3.A.1.1.-)	0.0	8.39E-01
Clo1313_2629	hypothetical protein	0.2	6.84E-01
Clo1313_2630	UV-damage endonuclease	-0.5	2.54E-02
Clo1313_2631	Methyltransferase type 11	-0.3	2.19E-01
Clo1313_2632	Peptidoglycan-binding domain 1 protein	-0.5	1.38E-05
Clo1313_2633	nucleotidyltransferase	-0.1	1.75E-01
Clo1313_2634	pseudouridine synthase, RluA family	0.2	1.12E-01
Clo1313_2635	glycoside hydrolase family 10	0.0	9.88E-01
Clo1313_2636	Pseudo gene	0.0	9.41E-01
Clo1313_2637	alkyl hydroperoxide reductase, F subunit	-0.3	9.64E-06
Clo1313_2638	peroxiredoxin	-0.3	1.12E-02
Clo1313_2639	hypothetical protein	-0.1	6.47E-01
Clo1313_2640	hypothetical protein	0.0	9.90E-01
Clo1313_2641	hypothetical protein	-0.1	7.14E-01
Clo1313_2642	cell division protein FtsK/SpoIIIE	0.0	7.97E-01
Clo1313_2643	hypothetical protein	0.0	9.49E-01
Clo1313_2644	hypothetical protein	0.3	1.16E-01
Clo1313_2645	hypothetical protein	0.3	1.42E-01
Clo1313_2646	hypothetical protein	-0.1	7.62E-01
Clo1313_2647	Tetratricopeptide TPR_1 repeat-containing protein	-0.1	7.35E-01
Clo1313_2648	hypothetical protein	0.1	7.03E-01
Clo1313_2649	hypothetical protein	0.5	7.01E-02
Clo1313_2650	hypothetical protein	0.1	7.86E-01
Clo1313_2651	Hedgehog/intein hint domain protein	-0.1	4.89E-01
Clo1313_2652	Pseudo gene	0.0	9.97E-01
Clo1313_2653	hypothetical protein	0.1	6.89E-01
Clo1313_2654	hypothetical protein	-0.6	9.68E-03
Clo1313_2655	hypothetical protein	-0.1	7.05E-01
Clo1313_2656	Pseudo gene	-0.3	5.04E-01
Clo1313_2657	hypothetical protein	0.2	6.57E-01
Clo1313_2658	hypothetical protein	-0.3	5.50E-01
Clo1313_2659	transposase IS116/IS110/IS902 family protein	-0.6	1.92E-04
Clo1313_2660	hypothetical protein	-0.1	7.66E-01
Clo1313_2661	hypothetical protein	0.3	3.05E-01
Clo1313_2662	Pseudo gene	0.3	5.60E-01
Clo1313_2663	transposase IS3/IS911 family protein	NA	NA
Clo1313_2664	transposase, IS4 family	0.0	8.96E-01
Clo1313_2665	hypothetical protein	-0.2	4.63E-01
Clo1313_2666	Hedgehog/intein hint domain protein	-0.1	7.76E-01
Clo1313_2667	Bifunctional DNA primase/polymerase	-0.1	8.90E-01
Clo1313_2668	Pseudo gene	0.1	9.04E-01
Clo1313_2669	hypothetical protein	0.1	5.14E-01
Clo1313_2670	hypothetical protein	0.1	7.06E-01
Clo1313_2671	Hedgehog/intein hint domain protein	0.0	9.27E-01
Clo1313_2672	hypothetical protein	0.4	8.05E-02
Clo1313_2673	Pseudo gene	-0.1	9.08E-01
Clo1313_2674	Pseudo gene	-0.3	2.74E-02
Clo1313_2675	hypothetical protein	0.3	6.26E-02
Clo1313_2676	Pseudo gene	0.2	5.79E-01
Clo1313_2677	Domain of unknown function DUF1910	0.0	9.62E-01
Clo1313_2678	Hedgehog/intein hint domain protein	0.0	9.94E-01
Clo1313_2679	Pseudo gene	0.2	5.26E-01
Clo1313_2680	hypothetical protein	0.0	8.90E-01
Clo1313_2681	Pseudo gene	-0.4	9.07E-05
Clo1313_2682	hypothetical protein	-0.3	4.32E-02
Clo1313_2683	protein of unknown function DUF1557	-0.1	4.81E-01
Clo1313_2684	hypothetical protein	0.3	1.78E-01
Clo1313_2685	Pseudo gene	0.1	6.02E-01
Clo1313_2686	transposase IS200-family protein	-0.7	7.82E-05
Clo1313_2687	hypothetical protein	0.1	4.10E-01
Clo1313_2688	hypothetical protein	0.6	9.45E-02
Clo1313_2689	Serine/threonine-protein kinase-like domain	0.4	1.43E-02
Clo1313_2690	Pseudo gene	-0.2	4.00E-01
Clo1313_2691	amidohydrolase 2	-0.4	4.97E-02
Clo1313_2692	Pseudo gene	0.3	2.63E-01
Clo1313_2693	Dockerin type 1	0.3	1.10E-02
Clo1313_2694	DNA helicase/exodeoxyribonuclease V, subunit A (EC 3.1.11.5)	0.0	8.68E-01
Clo1313_2695	DNA helicase/exodeoxyribonuclease V, subunit B (EC 3.1.11.5)	-0.2	3.31E-01
Clo1313_2696	hypothetical protein	0.0	9.31E-01
Clo1313_2697	Pseudo gene	-0.1	8.60E-01
Clo1313_2698	hypothetical protein	0.3	4.11E-01
Clo1313_2699	Pseudo gene	-0.5	2.89E-02
Clo1313_2700	Integrase catalytic region	NA	NA
Clo1313_2701	Pseudo gene	-0.1	6.37E-01
Clo1313_2702	hypothetical protein	0.0	9.00E-01
Clo1313_2703	hypothetical protein	0.0	9.39E-01
Clo1313_2704	Hedgehog/intein hint domain protein	0.1	5.69E-01

Clo1313_2705	hypothetical protein	0.3	3.11E-01
Clo1313_2706	protein of unknown function DUF324	0.1	8.37E-01
Clo1313_2707	CRISPR-associated protein, Csx7 family	-0.1	5.56E-01
Clo1313_2708	CRISPR-associated protein, Csx7 family	0.1	7.23E-01
Clo1313_2709	hypothetical protein	0.1	7.90E-01
Clo1313_2710	hypothetical protein	0.0	9.80E-01
Clo1313_2711	protein of unknown function DUF324	-0.1	7.42E-01
Clo1313_2712	hypothetical protein	-0.1	3.55E-01
Clo1313_2713	hypothetical protein	0.3	1.76E-01
Clo1313_2714	CRISPR-associated protein, TM1812 family	-0.2	1.97E-01
Clo1313_2715	Appr-1-p processing domain protein	0.0	9.94E-01
Clo1313_2716	RNA polymerase, sigma 30 subunit, SigH	0.4	1.19E-03
Clo1313_2717	hypothetical protein	NA	NA
Clo1313_2718	RNA methyltransferase, TrmH family, group 3	-0.3	2.74E-02
Clo1313_2719	ribonuclease III	0.2	3.61E-01
Clo1313_2720	GerA spore germination protein	-0.4	3.74E-01
Clo1313_2721	spore germination protein	-0.1	8.55E-01
Clo1313_2722	germination protein, Ger(x)C family	-0.2	6.16E-01
Clo1313_2723	cysteinyl-tRNA synthetase (EC 6.1.1.16)	-0.3	1.36E-02
Clo1313_2724	serine O-acetyltransferase	-0.2	1.23E-01
Clo1313_2725	membrane protein of unknown function	0.1	8.20E-01
Clo1313_2726	DNA-(apurinic or apyrimidinic site) lyase	-0.7	6.60E-20
Clo1313_2727	hypothetical protein	0.1	5.98E-01
Clo1313_2728	hypothetical protein	0.1	7.46E-01
Clo1313_2729	sporulation protein YtxC	-0.4	4.93E-02
Clo1313_2730	hypothetical protein	-0.1	8.75E-01
Clo1313_2731	amidohydrolase	0.2	2.87E-01
Clo1313_2732	protein of unknown function DUF378	0.0	9.54E-01
Clo1313_2733	hypothetical protein	-0.3	2.47E-01
Clo1313_2734	protein of unknown function DUF28	-0.2	2.23E-01
Clo1313_2735	integral membrane sensor signal transduction histidine kinase	0.1	5.37E-01
Clo1313_2736	CoA-binding domain protein	-0.2	2.51E-01
Clo1313_2737	Uncharacterized protein family UPF0029, Impact, N-terminal protein	-0.6	1.29E-08
Clo1313_2738	signal peptidase I	0.0	9.65E-01
Clo1313_2739	NUDIX hydrolase	0.4	2.50E-02
Clo1313_2740	GTP-binding proten HflX	0.2	2.15E-01
Clo1313_2741	hypothetical protein	0.4	2.79E-01
Clo1313_2742	DNA-directed DNA polymerase	0.8	5.64E-06
Clo1313_2743	deoxyuridine 5'-triphosphate nucleotidohydrolase Dut	-0.4	2.23E-02
Clo1313_2744	peptidase U32	-0.1	4.01E-01
Clo1313_2745	phosphoglycerate mutase (EC 5.4.2.1)	0.3	1.98E-02
Clo1313_2746	cell division protein ZapA	0.3	2.40E-02
Clo1313_2747	cellulose 1,4-beta-cellobiosidase (EC 3.2.1.91)	0.3	5.21E-05
Clo1313_2748	hypothetical protein	0.1	7.37E-01
Clo1313_2749	hypothetical protein	-0.1	8.93E-01
Clo1313_2750	dimethyladenosine transferase (EC 2.1.1.-)	-0.6	7.77E-05
Clo1313_2751	3D domain-containing protein	-0.8	9.17E-15
Clo1313_2752	hypothetical protein	0.4	1.60E-01
Clo1313_2753	hydrolase, TatD family	-0.4	8.54E-04
Clo1313_2754	methionyl-tRNA synthetase (EC 6.1.1.10)	-0.3	1.55E-03
Clo1313_2755	Protein of unknown function DUF2600	-0.2	2.16E-01
Clo1313_2756	nucleoside recognition domain protein	0.1	6.91E-01
Clo1313_2757	nucleoside recognition domain protein	0.5	1.12E-01
Clo1313_2758	transcriptional regulator, AbrB family	0.1	7.14E-01
Clo1313_2759	Uroporphyrin-III C/tetrapyrrole (Corrin/Porphyrin) methyltransferase	-0.5	8.85E-07
Clo1313_2760	Methyltransferase type 11	-0.4	6.84E-04
Clo1313_2761	4Fe-4S ferredoxin iron-sulfur binding domain protein	0.5	5.99E-02
Clo1313_2762	PSP1 domain protein	-0.6	8.57E-15
Clo1313_2763	AAA ATPase	-0.7	3.13E-19
Clo1313_2764	protein of unknown function DUF327	-0.5	6.73E-04
Clo1313_2765	thymidylate kinase	-0.6	2.00E-05
Clo1313_2766	arginine decarboxylase (EC 4.1.1.19)	-0.1	3.65E-01
Clo1313_2767	hypothetical protein	0.2	2.78E-01
Clo1313_2768	Beta propeller domain	-0.7	1.42E-20
Clo1313_2769	ABC transporter related protein	-0.1	7.95E-01
Clo1313_2770	protein of unknown function DUF214	-0.2	5.45E-01
Clo1313_2771	two component transcriptional regulator, winged helix family	-0.5	8.58E-03
Clo1313_2772	integral membrane sensor signal transduction histidine kinase	-0.3	9.94E-02
Clo1313_2773	protein of unknown function DUF77	-0.9	9.24E-09
Clo1313_2774	binding-protein-dependent transport systems inner membrane component	-0.4	5.41E-04
Clo1313_2775	NMT1/THI5 like domain protein	-0.3	2.15E-02
Clo1313_2776	ABC transporter related protein	-0.2	2.44E-01
Clo1313_2777	glycoside hydrolase family 10	-0.2	4.39E-02
Clo1313_2778	RNA polymerase sigma-I factor	-0.1	7.83E-01
Clo1313_2779	hypothetical protein	-0.3	6.31E-02

Clo1313_2780	hypothetical protein	0.1	7.72E-01
Clo1313_2781	Trypanosome RHS	0.5	8.50E-02
Clo1313_2782	hypothetical protein	0.3	2.92E-01
Clo1313_2783	binding-protein-dependent transport systems inner membrane component	0.5	6.26E-02
Clo1313_2784	binding-protein-dependent transport systems inner membrane component	0.3	3.48E-01
Clo1313_2785	ATPase associated with various cellular activities AAA_3	0.7	1.14E-03
Clo1313_2786	extracellular solute-binding protein family 1	-0.3	5.42E-02
Clo1313_2787	Pseudo gene	0.1	5.22E-01
Clo1313_2788	hypothetical protein	0.1	3.46E-01
Clo1313_2789	Protein of unknown function DUF2089	0.3	2.34E-01
Clo1313_2790	hypothetical protein	0.7	2.03E-07
Clo1313_2791	hypothetical protein	0.3	2.08E-01
Clo1313_2792	Pseudo gene	0.3	5.30E-01
Clo1313_2793	Carbohydrate binding family 6	0.1	4.90E-01
Clo1313_2794	glycoside hydrolase family 43	0.3	5.47E-01
Clo1313_2795	alpha-L-arabinofuranosidase B	-0.4	1.30E-01
Clo1313_2796	hypothetical protein	0.1	4.75E-01
Clo1313_2797	hypothetical protein	0.3	4.05E-01
Clo1313_2798	metal-dependent phosphohydrolase HD sub domain	0.2	3.89E-01
Clo1313_2799	Pseudo gene	0.1	8.04E-01
Clo1313_2800	DNA replication and repair protein RecR	-0.6	3.59E-10
Clo1313_2801	Uncharacterized protein family UPF0133	-0.5	2.19E-05
Clo1313_2802	DNA polymerase III, subunits gamma and tau	0.3	8.68E-03
Clo1313_2803	Pseudo gene	0.3	3.96E-01
Clo1313_2804	copper amine oxidase-like domain-containing protein	-0.1	7.47E-01
Clo1313_2805	glycoside hydrolase family 5	0.4	3.68E-03
Clo1313_2806	Carbohydrate-binding CenC domain protein	-0.5	2.73E-14
Clo1313_2807	amine oxidase	-0.4	2.06E-03
Clo1313_2808	hypothetical protein	-0.2	1.60E-01
Clo1313_2809	hypothetical protein	-0.2	7.49E-02
Clo1313_2810	GtrA family protein	0.0	9.66E-01
Clo1313_2811	Methyltransferase type 11	0.7	1.64E-02
Clo1313_2812	S-layer domain-containing protein	-0.4	6.56E-02
Clo1313_2813	hypothetical protein	0.1	6.77E-01
Clo1313_2814	hypothetical protein	0.3	5.55E-01
Clo1313_2815	hypothetical protein	0.1	7.55E-01
Clo1313_2816	Spore coat protein CotH	0.2	1.54E-01
Clo1313_2817	hypothetical protein	-0.4	3.39E-01
Clo1313_2818	putative autoinducer prepeptide	1.2	7.15E-06
Clo1313_2819	hypothetical protein	-0.3	4.14E-02
Clo1313_2820	peptidase C26	-0.2	2.08E-01
Clo1313_2821	anti-sigma-factor antagonist	-0.2	2.79E-01
Clo1313_2822	hypothetical protein	-0.4	2.20E-01
Clo1313_2823	Rubredoxin-type Fe(Cys)4 protein	-0.5	6.05E-02
Clo1313_2824	sporulation protein YyaC	-0.3	4.61E-01
Clo1313_2825	putative PAS/PAC sensor protein	-0.2	4.24E-02
Clo1313_2826	glucosyl hydrolase family protein	0.0	9.47E-01
Clo1313_2827	Propeptide PepSY amd peptidase M4	-0.5	9.94E-08
Clo1313_2828	metallophosphoesterase	0.3	3.38E-01
Clo1313_2829	hypothetical protein	0.5	2.33E-02
Clo1313_2830	type III restriction protein res subunit	0.3	1.04E-03
Clo1313_2831	LSU ribosomal protein L31P	-0.6	8.67E-06
Clo1313_2832	transcription termination factor Rho	-0.1	1.78E-01
Clo1313_2833	hypothetical protein	1.0	2.07E-04
Clo1313_2834	hypothetical protein	-0.1	5.22E-01
Clo1313_2835	Abortive infection protein	-0.6	2.43E-03
Clo1313_2836	hypothetical protein	-0.5	2.22E-01
Clo1313_2837	hypothetical protein	-0.1	8.91E-01
Clo1313_2838	hypothetical protein	0.1	7.59E-01
Clo1313_2839	hypothetical protein	-0.1	8.93E-01
Clo1313_2840	acyltransferase 3	0.5	5.90E-02
Clo1313_2841	hypothetical protein	0.6	2.12E-02
Clo1313_2842	pectate disaccharide-lyase	0.4	2.33E-02
Clo1313_2843	Pectate lyase/Amb allergen	0.8	5.33E-08
Clo1313_2844	Cl- channel voltage-gated family protein	0.3	1.19E-02
Clo1313_2845	PP-loop domain protein	1.4	6.73E-27
Clo1313_2846	Ig domain protein group 2 domain protein	1.0	6.49E-24
Clo1313_2847	UTP-glucose-1-phosphate uridylyltransferase	-0.2	5.58E-02
Clo1313_2848	ribonuclease BN	-0.3	5.20E-01
Clo1313_2849	SSU ribosomal protein S18P	-0.7	1.23E-11
Clo1313_2850	single-strand binding protein	-0.5	6.51E-07
Clo1313_2851	SSU ribosomal protein S6P	-0.5	1.77E-11
Clo1313_2852	diguanylate cyclase with GAF sensor	0.3	3.95E-03
Clo1313_2853	N-acetylglucosamine 6-phosphate deacetylase (EC 3.5.1.25)	0.7	2.60E-03
Clo1313_2854	1,4-alpha-glucan branching enzyme	0.1	3.15E-01
Clo1313_2855	hypothetical protein	-0.7	1.47E-04

Clo1313_2856	Carbohydrate binding family 6	-0.1	4.04E-01
Clo1313_2857	glycoside hydrolase family 43	-0.2	3.08E-01
Clo1313_2858	Carbohydrate binding family 6	-0.1	4.89E-01
Clo1313_2859	Carbohydrate binding family 6	-0.2	6.03E-01
Clo1313_2860	glycoside hydrolase family 43	0.1	8.09E-01
Clo1313_2861	glycoside hydrolase family 2 sugar binding protein	0.5	2.40E-01
Clo1313_2862	HNH nuclease	-0.4	1.49E-01
Clo1313_2863	hypothetical protein	-0.2	5.01E-01
Clo1313_2864	transcriptional regulator, MerR family	0.7	1.72E-02
Clo1313_2865	protein of unknown function DUF214	-0.1	8.30E-01
Clo1313_2866	ABC transporter related protein	0.3	4.72E-01
Clo1313_2867	histidine kinase (EC 2.7.13.3)	0.1	6.55E-01
Clo1313_2868	two component transcriptional regulator, winged helix family	-0.1	7.86E-01
Clo1313_2869	hypothetical protein	0.0	9.95E-01
Clo1313_2870	hypothetical protein	-0.6	7.06E-04
Clo1313_2871	hypothetical protein	-0.5	5.01E-05
Clo1313_2872	hypothetical protein	-0.3	1.96E-01
Clo1313_2873	transposase (DDE domain)	0.1	7.59E-01
Clo1313_2874	hypothetical protein	-0.2	4.97E-01
Clo1313_2875	hypothetical protein	-0.3	1.62E-02
Clo1313_2876	GTP cyclohydrolase I	-0.4	2.49E-03
Clo1313_2877	cyanophycin synthetase	0.0	8.79E-01
Clo1313_2878	cyanophycinase	-0.4	3.21E-01
Clo1313_2879	hypothetical protein	0.1	8.95E-01
Clo1313_2880	isoaspartyl dipeptidase	-0.1	7.35E-01
Clo1313_2881	transcriptional repressor, LexA family	0.0	7.91E-01
Clo1313_2882	3-isopropylmalate dehydrogenase	-0.3	1.91E-03
Clo1313_2883	3-isopropylmalate dehydratase, small subunit	-0.2	3.41E-02
Clo1313_2884	3-isopropylmalate dehydratase, large subunit	-0.6	3.67E-10
Clo1313_2885	transcriptional regulator, AraC family	-0.7	5.79E-13
Clo1313_2886	Protein of unknown function DUF2179	-0.4	1.13E-02
Clo1313_2887	hypothetical protein	-0.2	3.09E-01
Clo1313_2888	Mg2 transporter protein CorA family protein	-0.8	6.42E-11
Clo1313_2889	FlgN family protein	-0.2	6.92E-02
Clo1313_2890	flagellar protein FliS	-0.2	2.69E-02
Clo1313_2891	flagellar hook-associated 2 domain-containing protein	-0.8	2.22E-30
Clo1313_2892	flagellar protein FlaG protein	0.2	2.75E-01
Clo1313_2893	NAD-dependent epimerase/dehydratase	0.3	6.54E-02
Clo1313_2894	UDP-4-keto-6-deoxy-N-acetylglucosamine 4-aminotransferase	0.6	6.70E-05
Clo1313_2895	pseudaminic acid biosynthesis-associated protein PseG	0.6	1.49E-05
Clo1313_2896	LmbE family protein	0.4	9.09E-03
Clo1313_2897	methionyl-tRNA formyltransferase	0.2	3.20E-01
Clo1313_2898	methylmalonyl-CoA epimerase (EC 5.1.99.1)	0.1	7.78E-01
Clo1313_2899	FkbH like protein	-0.3	5.61E-02
Clo1313_2900	phosphopantetheine-binding protein	-0.3	4.59E-02
Clo1313_2901	pseudaminic acid biosynthesis N-acetyl transferase	-0.5	4.67E-05
Clo1313_2902	N-acetylneuraminate synthase (EC 2.5.1.56)	-0.8	1.38E-19
Clo1313_2903	acylneuraminate cytidylyltransferase	0.0	8.75E-01
Clo1313_2904	pseudaminic acid biosynthesis-associated methylase	-0.1	7.47E-01
Clo1313_2905	polysaccharide biosynthesis protein CapD	0.1	6.72E-01
Clo1313_2906	protein of unknown function DUF115	0.4	5.02E-02
Clo1313_2907	hypothetical protein	0.3	1.81E-01
Clo1313_2908	protein of unknown function DUF115	0.5	1.50E-03
Clo1313_2909	flagellin domain protein	0.6	6.72E-10
Clo1313_2910	flagellin domain protein	0.5	6.24E-07
Clo1313_2911	Aldehyde Dehydrogenase	0.0	9.42E-01
Clo1313_2912	carbon storage regulator, CsrA	0.2	1.10E-01
Clo1313_2913	protein of unknown function DUF180	-0.3	3.50E-02
Clo1313_2914	hypothetical protein	-0.8	3.75E-12
Clo1313_2915	flagellar hook-associated protein 3	0.5	2.39E-03
Clo1313_2916	flagellar hook-associated protein FlgK	0.3	1.33E-02
Clo1313_2917	flagellar hook-associated protein FlgK	0.3	2.50E-02
Clo1313_2918	FlgN family protein	0.2	2.94E-01
Clo1313_2919	anti-sigma-28 factor, FlgM family	0.1	5.37E-01
Clo1313_2920	regulatory protein, MerR	0.0	8.49E-01
Clo1313_2921	phosphoribosyltransferase	-0.4	8.46E-02
Clo1313_2922	helicase, RecD/TraA family	0.1	7.38E-01
Clo1313_2923	hypothetical protein	0.6	9.78E-02
Clo1313_2924	methionine adenosyltransferase (EC 2.5.1.6)	0.3	2.24E-03
Clo1313_2925	Thioesterase superfamily	-0.2	1.51E-01
Clo1313_2926	ATP-dependent metalloprotease FtsH	-0.5	5.48E-15
Clo1313_2927	hypoxanthine phosphoribosyltransferase	0.2	3.81E-01
Clo1313_2928	tRNA(Ile)-lysidine synthetase	-0.3	4.11E-02
Clo1313_2929	replicative DNA helicase	-0.5	1.20E-04
Clo1313_2930	LSU ribosomal protein L9P	-0.5	1.55E-05
Clo1313_2931	phosphoesterase RecJ domain protein	-0.6	7.76E-17
Clo1313_2932	hypothetical protein	-0.2	6.54E-02
Clo1313_2933	Prephenate dehydratase	-0.3	3.81E-02

Clo1313_2934	hypothetical protein	-0.2	1.85E-01
Clo1313_2935	V-type ATPase 116 kDa subunit	-0.3	4.05E-03
Clo1313_2936	H+transporting two-sector ATPase C subunit	-0.5	1.89E-03
Clo1313_2937	H+transporting two-sector ATPase E subunit	-0.8	3.67E-13
Clo1313_2938	H+transporting two-sector ATPase C (AC39) subunit	-0.6	6.19E-08
Clo1313_2939	Vacuolar H+transporting two-sector ATPase F subunit	-0.3	1.91E-01
Clo1313_2940	H+transporting two-sector ATPase alpha/beta subunit central region	-0.2	5.61E-02
Clo1313_2941	H+transporting two-sector ATPase alpha/beta subunit central region	-0.2	6.73E-02
Clo1313_2942	V-type ATPase, D subunit	-0.3	3.34E-02
Clo1313_2943	ABC transporter related protein	0.0	9.94E-01
Clo1313_2944	Dockerin type 1	0.4	3.24E-01
Clo1313_2945	hypothetical protein	0.1	8.39E-01
Clo1313_2946	hypothetical protein	0.0	9.52E-01
Clo1313_2947	hypothetical protein	0.2	3.91E-01
Clo1313_2948	Pseudo gene	0.1	7.05E-01
Clo1313_2949	hypothetical protein	-0.3	2.42E-01
Clo1313_2950	AAA ATPase central domain protein	-0.1	4.89E-01
Clo1313_2951	hypothetical protein	-0.3	4.88E-02
Clo1313_2952	amino acid ABC transporter substrate-binding protein, PAAT family (TC 3.A.1.3.-)	-0.4	1.93E-04
Clo1313_2953	amino acid ABC transporter membrane protein, PAAT family (TC 3.A.1.3.-)	-0.4	1.18E-03
Clo1313_2954	amino acid ABC transporter ATP-binding protein, PAAT family (TC 3.A.1.3.-)	-0.5	1.88E-05
Clo1313_2955	response regulator receiver modulated CheB methylesterase	-0.1	4.76E-01
Clo1313_2956	MCP methyltransferase, CheR-type	0.0	8.57E-01
Clo1313_2957	methyl-accepting chemotaxis sensory transducer	0.2	2.01E-01
Clo1313_2958	CheA signal transduction histidine kinase	0.2	1.33E-01
Clo1313_2959	CheW protein	0.1	5.64E-01
Clo1313_2960	Pseudo gene	0.1	8.90E-01
Clo1313_2961	integrase family protein	0.1	7.69E-01
Clo1313_2962	transcriptional regulator, AraC family	0.3	1.09E-01
Clo1313_2963	PEBP family protein	0.1	8.34E-01
Clo1313_2964	hypothetical protein	-0.1	8.65E-01
Clo1313_2965	ABC transporter related protein	0.0	9.62E-01
Clo1313_2966	hypothetical protein	0.0	9.54E-01
Clo1313_2967	hypothetical protein	0.3	2.60E-01
Clo1313_2968	hypothetical protein	0.0	9.74E-01
Clo1313_2969	CRISPR-associated protein Cas2	-0.2	6.22E-01
Clo1313_2970	CRISPR-associated protein Cas1	-0.1	8.60E-01
Clo1313_2971	CRISPR-associated exonuclease, Cas4 family	-0.6	1.07E-01
Clo1313_2972	CRISPR-associated helicase, Cas3 family	-0.6	1.09E-01
Clo1313_2973	CRISPR-associated protein, Cas5 family	0.3	3.15E-01
Clo1313_2974	CRISPR-associated autoregulator, Cst2 family	0.4	1.95E-01
Clo1313_2975	hypothetical protein	-0.1	8.54E-01
Clo1313_2976	CRISPR-associated protein, Cas6 family	0.3	4.75E-01
Clo1313_2977	hypothetical protein	-0.3	6.72E-02
Clo1313_2978	Undecaprenyl-diphosphatase (EC 3.6.1.27)	-0.2	4.29E-02
Clo1313_2979	MATE efflux family protein	-0.2	9.34E-02
Clo1313_2980	hemerythrin-like metal-binding protein	0.9	4.91E-06
Clo1313_2981	CheC domain protein	0.6	1.24E-02
Clo1313_2982	Nitrilase/cyanide hydratase and apolipoprotein N-acyltransferase	-1.0	9.50E-24
Clo1313_2983	copper amine oxidase-like domain-containing protein	0.3	4.74E-01
Clo1313_2984	hypothetical protein	-0.2	2.56E-02
Clo1313_2985	FMN-binding domain protein	-0.2	1.12E-01
Clo1313_2986	protein of unknown function DUF218	0.5	9.36E-04
Clo1313_2987	protein of unknown function DUF163	0.0	8.75E-01
Clo1313_2988	beta-lactamase domain protein	-0.2	2.28E-01
Clo1313_2989	UDP-N-acetylglucosamine 1-carboxyvinyltransferase	0.1	3.22E-01
Clo1313_2990	Protein of unknown function YycH	0.1	6.11E-01
Clo1313_2991	hypothetical protein	0.2	7.19E-02
Clo1313_2992	hypothetical protein	-0.3	4.32E-02
Clo1313_2993	multi-sensor signal transduction histidine kinase	-0.4	1.55E-04
Clo1313_2994	two component transcriptional regulator, winged helix family	-0.5	1.11E-05
Clo1313_2995	hypothetical protein	0.0	9.31E-01
Clo1313_2996	putative transcriptional regulator, TetR family	0.0	9.95E-01
Clo1313_2997	polysaccharide biosynthesis protein CapD	-0.2	3.18E-02
Clo1313_2998	polysaccharide biosynthesis protein	0.0	9.56E-01
Clo1313_2999	glycosyl transferase group 1	-0.2	3.39E-01
Clo1313_3000	hypothetical protein	0.0	8.15E-01
Clo1313_3001	glycosyl transferase group 1	-0.4	6.63E-04
Clo1313_3002	glycosyl transferase group 1	-0.8	4.47E-17
Clo1313_3003	nucleotide sugar dehydrogenase	-0.4	3.61E-05
Clo1313_3004	glycosyl transferase family 2	-0.6	4.01E-11
Clo1313_3005	hypothetical protein	-0.6	4.37E-07

Clo1313_3006	Acetyltransferase, GNAT family	-0.6	2.27E-05
Clo1313_3007	Undecaprenyl-phosphate galactose phosphotransferase	-1.0	3.41E-16
Clo1313_3008	Glutamine--scyllo-inositol transaminase	-0.6	9.02E-14
Clo1313_3009	O-antigen polymerase	-0.5	2.17E-07
Clo1313_3010	lipopolysaccharide biosynthesis protein	-0.2	9.00E-02
Clo1313_3011	Ig domain protein	-0.2	3.51E-02
Clo1313_3012	hypothetical protein	-0.1	7.75E-01
Clo1313_3013	cell wall hydrolase SleB	0.4	3.56E-01
Clo1313_3014	hypothetical protein	0.2	6.59E-01
Clo1313_3015	putative signal transduction protein with CBS domains	-0.6	4.92E-10
Clo1313_3016	Ig domain protein group 1 domain protein	0.1	7.33E-01
Clo1313_3017	nicotinate-nucleotide pyrophosphorylase [carboxylating] (EC 2.4.2.19)	-0.3	1.51E-02
Clo1313_3018	L-aspartate oxidase (EC 1.4.3.16)	-0.2	2.84E-01
Clo1313_3019	quinolinate synthetase (EC 2.5.1.72)	0.0	8.57E-01
Clo1313_3020	hypothetical protein	-0.2	2.07E-01
Clo1313_3021	Accessory gene regulator B	0.0	9.38E-01
Clo1313_3022	metal dependent phosphohydrolase	-0.3	1.46E-01
Clo1313_3023	endoglucanase Cel9U	0.1	3.05E-01
Clo1313_3024	DNA gyrase subunit A (EC 5.99.1.3)	-0.2	4.53E-02
Clo1313_3025	parB-like partition protein	0.1	6.53E-01
Clo1313_3026	16S rRNA m(7)G-527 methyltransferase (EC 2.1.1.170)	-0.2	2.75E-01
Clo1313_3027	glucose inhibited division protein A	-0.3	9.95E-04
Clo1313_3028	tRNA modification GTPase trmE	-0.4	7.23E-04
Clo1313_3029	single-stranded nucleic acid binding R3H domain-containing protein	-0.5	1.88E-04
Clo1313_3030	membrane protein insertase, YidC/Oxa1 family	-0.5	9.35E-12
Clo1313_3031	protein of unknown function DUF37	0.1	7.56E-01
Clo1313_3032	ribonuclease P protein component (EC 3.1.26.5)	-0.4	7.95E-05
Clo1313_3033	LSU ribosomal protein L34P	-0.5	2.83E-04
Clo1313_R0001	(tRNA)	-0.4	2.60E-01
Clo1313_R0002	(tRNA)	-0.1	7.27E-01
Clo1313_R0003	(tRNA)	-0.1	6.27E-01
Clo1313_R0004	(tRNA)	-0.1	4.72E-01
Clo1313_R0005	(tRNA)	NA	NA
Clo1313_R0006	(tRNA)	0.0	9.28E-01
Clo1313_R0007	(tRNA)	0.1	8.54E-01
Clo1313_R0008	(tRNA)	NA	NA
Clo1313_R0009	(tRNA)	-0.1	8.85E-01
Clo1313_R0010	(tRNA)	0.1	6.92E-01
Clo1313_R0011	(tRNA)	NA	NA
Clo1313_R0013	(rRNA 16S)	NA	NA
Clo1313_R0014	(tRNA)	-0.1	5.62E-01
Clo1313_R0015	(rRNA 23S)	0.0	7.05E-01
Clo1313_R0016	(rRNA 5S)	NA	NA
Clo1313_R0017	(rRNA 16S)	-0.1	7.75E-01
Clo1313_R0018	(rRNA 23S)	0.5	1.96E-01
Clo1313_R0019	(rRNA 5S)	NA	NA
Clo1313_R0020	(tRNA)	0.0	9.27E-01
Clo1313_R0021	(tRNA)	0.0	9.27E-01
Clo1313_R0022	(tRNA)	0.2	7.50E-01
Clo1313_R0023	(tRNA)	0.3	5.45E-01
Clo1313_R0024	(tRNA)	0.1	7.58E-01
Clo1313_R0025	(rRNA 16S)	0.2	6.15E-01
Clo1313_R0026	(tRNA)	NA	NA
Clo1313_R0027	(rRNA 23S)	0.2	6.02E-01
Clo1313_R0028	(rRNA 5S)	NA	NA
Clo1313_R0029	(tRNA)	NA	NA
Clo1313_R0030	(tRNA)	0.0	7.05E-01
Clo1313_R0031	(tRNA)	0.1	6.89E-01
Clo1313_R0032	(tRNA)	0.1	6.63E-01
Clo1313_R0033	(tRNA)	0.1	5.91E-01
Clo1313_R0034	(tRNA)	NA	NA
Clo1313_R0035	(tRNA)	0.0	9.78E-01
Clo1313_R0036	(tRNA)	NA	NA
Clo1313_R0037	(tRNA)	0.1	5.40E-01
Clo1313_R0038	(tRNA)	-0.1	6.47E-01
Clo1313_R0039	(tRNA)	NA	NA
Clo1313_R0040	rnpB; RNA component of RNaseP	0.4	3.86E-02
Clo1313_R0041	(tRNA)	-0.1	6.27E-01
Clo1313_R0042	(tRNA)	NA	NA
Clo1313_R0043	(tRNA)	NA	NA
Clo1313_R0044	(tRNA)	0.3	3.28E-01
Clo1313_R0045	(tRNA)	0.1	6.80E-01
Clo1313_R0046	(tRNA)	NA	NA
Clo1313_R0047	(tRNA)	0.0	9.60E-01
Clo1313_R0048	(tRNA)	-0.1	7.31E-01
Clo1313_R0049	(tRNA)	0.1	7.76E-01

Clo1313_R0050	(tRNA)	0.1	7.09E-01
Clo1313_R0051	(tRNA)	-0.3	4.62E-01
Clo1313_R0052	(tRNA)	0.0	8.24E-01
Clo1313_R0053	(tRNA)	NA	NA
Clo1313_R0054	(tRNA)	NA	NA
Clo1313_R0055	(tRNA)	NA	NA
Clo1313_R0056	(tRNA)	0.0	7.70E-01
Clo1313_R0057	(tRNA)	-0.1	4.81E-01
Clo1313_R0058	(tRNA)	-0.1	6.47E-01
Clo1313_R0059	(tRNA)	0.4	2.22E-01
Clo1313_R0060	(tRNA)	-0.2	5.34E-01
Clo1313_R0061	(tRNA)	0.0	9.68E-01
Clo1313_R0062	(tRNA)	0.1	5.40E-01
Clo1313_R0063	(ncRNA)	0.6	2.06E-06
Clo1313_R0064	ncRNA	-0.2	5.75E-01
Clo1313_R0065	(tRNA)	0.0	9.38E-01
Clo1313_R0066	(tRNA)	-0.1	6.69E-01
Clo1313_R0067	(tRNA)	0.1	6.71E-01
Clo1313_R0068	(tRNA)	0.1	7.64E-01
Clo1313_R0069	(rRNA 5S)	NA	NA
Clo1313_R0070	(rRNA 23S)	0.2	6.70E-01
Clo1313_R0071	(tRNA)	NA	NA
Clo1313_R0072	(rRNA 16S)	0.1	7.23E-01
